# The impact of fire on the Late Paleozoic Earth system

**DOI:** 10.3389/fpls.2015.00756

**Published:** 2015-09-23

**Authors:** Ian J. Glasspool, Andrew C. Scott, David Waltham, Natalia Pronina, Longyi Shao

**Affiliations:** ^1^Department of Geology, Colby CollegeWaterville, ME, USA; ^2^Science and Education, Field Museum of Natural HistoryChicago, IL, USA; ^3^Department of Earth Sciences, Royal Holloway University of LondonEgham, Surrey, UK; ^4^Faculty of Geology, Moscow State UniversityMoscow, Russia; ^5^State Key Laboratory of Coal Resources and Safe Mining, and School of Geosciences and Survey Engineering, China University of Mining and TechnologyBeijing, China

**Keywords:** fire, inertinite, charcoal, oxygen, climate, Paleozoic, flammability

## Abstract

Analyses of bulk petrographic data indicate that during the Late Paleozoic wildfires were more prevalent than at present. We propose that the development of fire systems through this interval was controlled predominantly by the elevated atmospheric oxygen concentration (p(O_2_)) that mass balance models predict prevailed. At higher levels of p(O_2_)_,_ increased fire activity would have rendered vegetation with high-moisture contents more susceptible to ignition and would have facilitated continued combustion. We argue that coal petrographic data indicate that p(O_2_) rather than global temperatures or climate, resulted in the increased levels of wildfire activity observed during the Late Paleozoic and can, therefore, be used to predict it. These findings are based upon analyses of charcoal volumes in multiple coals distributed across the globe and deposited during this time period, and that were then compared with similarly diverse modern peats and Cenozoic lignites and coals. Herein, we examine the environmental and ecological factors that would have impacted fire activity and we conclude that of these factors p(O_2_) played the largest role in promoting fires in Late Paleozoic peat-forming environments and, by inference, ecosystems generally, when compared with their prevalence in the modern world.

## Introduction

Fire is an important part of the Earth system (Bowman et al., 2009) and its roles in climate feedbacks and forcing are becoming better constrained (Bowman et al., 2009; Belcher, 2013; Scott et al., 2014). To understand the evolution of the Earth system in deep time, the role of ancient fires also needs to be taken into account (Scott, 2000, 2010; Pausas and Keeley, 2009; Belcher et al., 2013; Scott et al., 2014); however, our understanding of this phenomenon is itself still developing.

In investigating ancient fire systems, it is necessary to understand the primary factors controlling combustion. One of these factors, p(O_2_) is generally little considered by those studying modern wildfires as it is effectively a constant (present atmospheric oxygen level (PAL) = ∼21%). However, over geological time mass balance modeling suggests there were periods throughout the Phanerozoic when p(O_2_) differed significantly from the PAL (Berner et al., 2003; Hansen and Wallmann, 2003; Bergman et al., 2004; Berner, 2006, 2009; Kump, 2010; Lenton, 2013). Significantly, it has been recognized for more than 30 years that there is a relationship between the occurrence of fire in the fossil record and p(O_2_) (Cope and Chaloner, 1980). In a series of experiments Watson et al. (1978) demonstrated that as oxygen levels increased so plants with higher moisture contents became liable to combust (see also Watson and Lovelock, 2013) and conversely that as levels fell below PAL so combustion became impossible. This relationship between p(O_2_) and flammability means that these fluctuations in p(O_2_) over the Phanerozoic should have had a profound effect on fire occurrences (Berner et al., 2003; Scott and Glasspool, 2006; Belcher and McElwain, 2008; Glasspool and Scott, 2010; Kump, 2010; Lenton, 2013; Scott et al., 2014). Studies are showing increasingly that this is so, with p(O_2_) highs being increasingly correlated with global ‘high-fire’ conditions (e.g., Brown et al., 2012; Belcher et al., 2013; Scott et al., 2014). In addition to the effects of p(O_2_) on fire, additional data on fluctuations in Late Paleozoic p(O_2_) should help to elucidate potential relationships to changes in climate and faunal evolution, radiation, and size over this interval (e.g., Poulsen et al., 2015).

Fire is an exothermic oxidation reaction dependent on the rapid combination of fuel and oxygen in the presence of heat (Jones and Chaloner, 1991). From this it can be concluded the primary controls on fire are sources of fuel, heat, and a supply of oxygen. To relate wildfire occurrence in deep time to p(O_2_) it is necessary to decouple both sources of heat and fuel from this relationship as limiting factors.

While meteor strikes, volcanic activity, spontaneous combustion, and even rock fall may act as the sources of heat to ignite wildfires, the vast majority of fossil wildfires are considered to have been initiated by lightning strikes (Cope and Chaloner, 1980). At present lightning strikes occur at a rate of 44 ± 5 strikes/s across the globe (Christian et al., 2003). The occurrence of fulgurites in the fossil record demonstrates the occurrence of lightning in deep time, and it is generally considered that a lack of lightning strikes is unlikely to have been a limiting factor on fire ignition (Scott and Jones, 1991, 1994). Perhaps surprisingly, recent research on modern ecosystems indicates that the number of lightning strikes does not even have a direct relationship to the total area burnt, largely due to the extremely skewed nature of fire size, in which extremely large fires only propagate under weather conditions suitable for fuel production and rapid fire spread (Bistinas et al., 2014).

All terrestrial vegetation has the potential to be fuel. As the record of fossil wildfire dates back at least to the latest Silurian (Glasspool et al., 2004) and, with the exception of a few gaps, there is continuous evidence of charcoal from this time onward (Scott and Glasspool, 2006; Diessel, 2010; Glasspool and Scott, 2010; Rimmer et al., 2015), globally there must have been a source of fuel from about 419 million years through to the present. However, in the fossil record the distribution of biomass has varied both spatially and temporally. Peat-forming environments are by definition regions of biomass accumulation and in this environment an absence of fire ignition cannot be attributed to an absence of vegetation (Glasspool and Scott, 2010).

However, while these peat-forming environments may be vegetated this does not presuppose that this vegetation is combustible under the prevailing environmental conditions. Vegetation is heterogeneous in composition, where in terms of flammability the most important heterogeneity is moisture content (Whelan, 1995). For fuel to ignite, it must be heated sufficiently to first drive-off moisture and then to liberate volatiles that can be oxidized to generate a self-supporting exothermic pyrolytic reaction (i.e., fire). The greater the moisture content of a fuel the more energy that must be expended to drive that moisture off before volatiles can be liberated and so the less flammable a fuel is the more moisture it contains (Whelan, 1995). While not immune to fluctuations in moisture content, peat-forming environments do require that “groundwater must remain throughout the whole year, above or close to the ground surface” (Taylor et al., 1998). Therefore, these environments can be viewed as “high-moisture” settings where typical variations in weather and climate are less likely to have an impact on fire occurrence. Glasspool and Scott (2010) presented charcoal data from a range of Modern-Pleistocene aged peats representing divergent ecological settings and vegetation types to support this supposition, concluding that despite profound variations in weather and climate these settings showed consistently low levels of charcoal accumulation and hence wildfire activity and that, therefore, these settings reduced (but did not eliminate) the role fluctuations of moisture play on flammability.

While increasing moisture content reduces fuel flammability there is considerable experimental evidence that indicates this can be greatly off-set by the prevailing p(O_2_). Calculation of fuel flammability at varying oxygen concentrations enables past p(O_2_) to be constrained within the range 16–30% [“fire window” (Cope and Chaloner, 1980; Chaloner, 1989)] whenever charcoal is recovered from the fossil record (Belcher et al., 2010b, 2013; Watson and Lovelock, 2013). These experiments indicate that below, 16% p(O_2_) fires will not propagate no matter how minimal the moisture content of the fuel available. However, at levels above 21% fires will ignite more readily and at levels much above 23% they become highly prevalent (Belcher et al., 2010b, 2013). These findings make clear that as p(O_2_) climbs so the moisture content of fuel has less bearing on whether it is liable to combust, even high-moisture content fuels becoming readily flammable. Therefore, we should expect that the Late Paleozoic, a geological interval widely agreed to have experienced p(O_2_) greatly elevated above present, would have been a “high-fire world”.

Fires are not only directly impacted by atmospheric composition, but also feedback back onto it, in the short-term elevating CO_2_ levels while potentially decreasing them in the long term through carbon sequestration in the form of charcoal burial (Berner et al., 2003; Lehmann et al., 2006; Masek, 2013). However, fires may also impact climate change through other mechanisms, for example through the impact of smoke and black carbon on radiative energy (Bowman et al., 2009). This impact may have been of particular relevance during the latest Paleozoic, an interval that saw extensive southern polar ice accumulation (Rygel et al., 2008), in that in modern settings black carbon deposited on snow has been noted to impact ice cap melt rates (Keegan et al., 2014).

The role of fire on some elements of the latest Paleozoic flora has already been considered (Robinson, 1989, 1990, 1991) but some of her arguments have been shown not to stand up with new data (Rimmer et al., 2015). However, our knowledge of both fire frequency and feedback mechanisms has developed considerably since this work and the subject is worth revisiting as part of an assessment of the impacts of this phenomenon on the latest Paleozoic world.

## Materials and Methods

Coals and lignites are compressed and altered peats (Taylor et al., 1998), and are widely distributed both spatially and temporally throughout the Phanerozoic. Due to their economic importance these deposits have been extensively characterized and reported. One routine method of characterization is optical reflectance microscopy, whereby the organic constituents are described visually in terms of macerals (Taylor et al., 1998). One maceral group (inertinite) is almost exclusively considered the by-product of wildfires and is synonymous with charcoal (Scott and Glasspool, 2007; Glasspool and Scott, 2013). The amount of inertinite in a coal is commonly reported on a percentage by volume basis (either including or excluding the mineral matter content of the coal) and, therefore, provides an extensive record of charcoal abundance (Glasspool and Scott, 2010). To standardize the data for this paper, where mineral matter was included in the volumetric count, the inertinite content of a coal (Inert%) was recalculated and is presented on a mineral matter free (m.m.f.) basis. Much of the bulk data on inertinite in coals, used herein, was first published in Glasspool and Scott (2010). However, these data are augmented by new and previously unincorporated results, expanding the number of seams analyzed by >40% and taking into account the revised stratigraphic framework for the Phanerozoic published by Cohen et al. (2013). These data include >400 new data points for the interval spanning the Famennian to the Early Triassic. Of particular note are new data points from the Permian of Russia, China, and Australia (e.g., Smyth, 1972; Huleatt, 1991; Finkelman et al., 2000; Brownfield et al., 2001; Tewalt et al., 2010; Hudspith et al., 2012; Shao et al., 2012; Supplementary Table S1).

**FIGURE 1:**
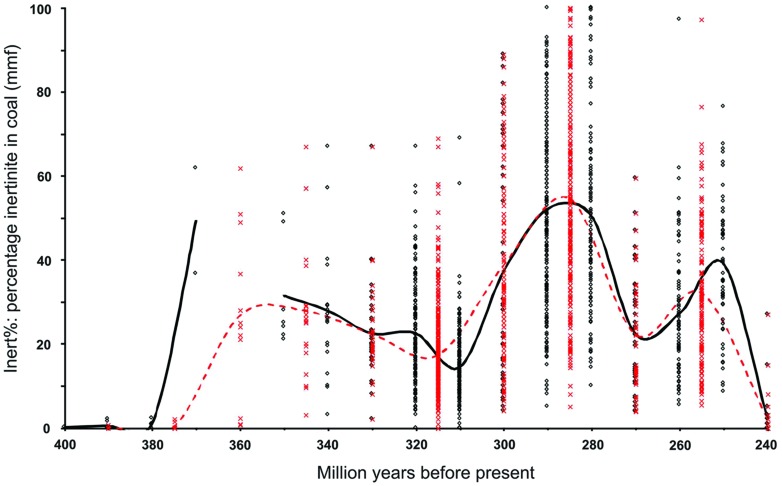
**The distribution of inertinite (charcoal) in coal.** Based on data from Glasspool and Scott (2010) with additional data added. The raw inertinite data are presented up to 240 Mya. Crosses, data binned to 15 million years. Circles, data binned to 10 million years. Dashed red line, average inertinite data binned by 15 million year intervals. Solid black line, average inertinite data binned by 10 million year intervals.

Maceral data from the literature, used to determine Inert% (charcoal in coal) were only included in this analysis where the inclusion/exclusion of mineral matter was clear. These data were then aggregated into both 10- and 15-million year binning intervals and averaged (Supplementary Table S2; **Figure 1**). It should be noted that binning the data can present some apparent anomalies, especially when data are compared graphically with an absolute chronostratigraphic framework, e.g., latest Permian inertinite data bin at 250 million years, an apparently earliest Triassic age. With two exceptions, coals whose stratigraphic resolution was greater than 15 million years were excluded (e.g., Taiyuan Formation = Kasimovian–Sakmarian). The two exceptions included in the database derive from poorly sampled stratigraphic intervals where they represent the only data: Givetian–Frasnian (Weatherall–Hecla Bay–Beverley Inlet formations) and the Anisian–Carnian (Basin Creek and Mungaroo formations). Where not tabulated or stated in the text, data were measured from graphics by pasting the image into Corel-Draw and overlaying guidelines to obtain exact measurements of data point positions. Preference was given to literature citing named seams. Where multiple references provide data from one seam, this data was averaged and all references cited.

To calculate p(O_2_) from Inert%, it was necessary to generate calibration curves. Our curves for converting observed inertinite concentration into estimates of past p(O_2_) are based upon three known points:

(1) Present day p(O_2_) = 21% and is associated with a mean inertinite concentration of 4.27 ± 0.64% (1 SE): (data from Supplementary Tables S3 and S4; based on 21 ecologically, climatically and geographically differing peats of Modern-to-Pleistocene age).
(2) As discussed above, experimental data indicate that wildfires are unsustainable at levels of p(O_2_) = 16% and hence, at this point, inertinite concentration should be 0%.
(3) Prior research indicates that in the Late Paleozoic p(O_2_) exceeded 25% (Wildman et al., 2004), but due to increased plant flammability was less than 30% (Jones and Chaloner, 1991; Lenton and Watson, 2000; Wildman et al., 2004; Belcher and McElwain, 2008; Belcher et al., 2010b, 2013; Watson and Lovelock, 2013). Focusing on the best temporally constrained dataset (10-million year binning), Inert% for the Late Paleozoic reaches an averaged maximum value of 50 ± 2% (1 SE) at 280 Ma (Supplementary Table S2). We make the assumption that, around 280 Ma, the high inertinite concentrations are associated with high p(O_2_). The precise p(O_2_) level is not known but it must be <30% since, otherwise, uncontrolled global wildfires would have resulted and there is no evidence for these. Hence, we assume that p(O_2_) = 28 ± 2% which encompasses a wide range of plausible values and spans the scope outlined above.

**FIGURE 2:**
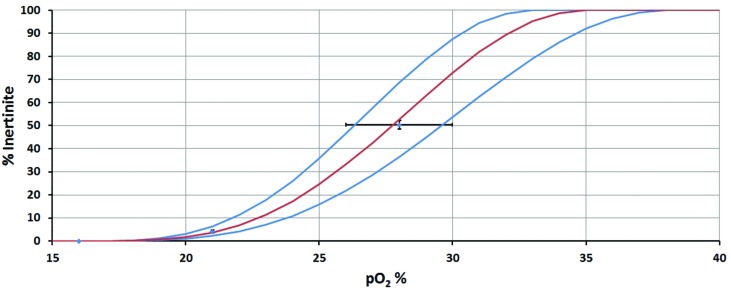
**Inertinite to p(O_2_) calibration curve.** Points, and associated error bars, show the data constraints. S-shaped curves are assumed, to ensure smooth transition from 0% inertinite to 100% inertinite.

The fixed points and error bars are plotted in **Figure 2**.

The fitted curves in **Figure 2** are assumed to be S-shaped. This ensures a smooth transition from 0% inertinite at low oxygen levels to 100% inertinite at high oxygen levels. In reality it is not known whether the maximum inertinite could indeed be 100% as it may peak at some lower level (and perhaps even fall thereafter). However, the precise details of the calibration curve above p(O_2_) = 30% are relatively unimportant as this region of the plot is not used in practice. The curves used here are of the form:

(1) $$\begin{array}{lll} I & ={\left(0.5-0.5\cos\left[\pi (o-o_{\min})/\left(o_{\max}-o_{\min}\right)\right]\right)}^{n} & o_{\min}<o<o_{\max}\\ & =0\% & o\leq o_{\min}\\ & =100\% & o\geq o_{\max} \end{array}\,$$

where *I* is the inertinite concentration, o is the oxygen level, *o*_min_ is the oxygen level for no inertinite, *o*_max_ is the oxygen level when inertinite reaches 100% and *n* controls the maximum steepness of the S-curve. The chosen values of these parameters are given in **Table 1**.

**Table 1:** Parameters used in Eq. (1) to produce the curves shown in **Figure 2**.

	Best	Max	Min
*o*_min_ (%)	16	16	16
*o*_max_ (%)	35	33	38
*n*	1.8	1.7	1.8


The final curves shown in **Figure 2** are then used to produce a best estimate and uncertainty for p(O_2_) as follows. The mean inertinite concentration, *Ī*, and its SE, *s*, are calculated within any given age-bin. This mean is then inserted into Eq. 1 along with the best-fit parameters from **Table 1** to give our best estimate of *o*. The minimum estimate is produced by inserting parameters from the maximum column of **Table 1** [N.B. the upper curve in **Figure 2** gives the minimum p(O_2_)] along with an inertinite concentration given by *I* = *Ī* - *s*. Similarly, the maximum estimate is given by the minimum parameters in **Table 1** together with *I* = *Ī* + *s*.

While these are significant assumptions, they appear to be supported by mass balance, biogeochemical, and carbon isotopic fractionation models independent of fire data. These models predict maximal Phanerozoic p(O_2_) during the Permian at ∼30–35% (e.g., Berner and Canfield, 1989; Beerling et al., 1998, 2002; Berner, 2006, 2009). The timing of these maximal p(O_2_) data corresponds well with the timing of maximal inertinite abundance [i.e., Early Permian (280- and 285-million year bins)].

## Results

Despite adding numerous new data points on Late Paleozoic inertinite in coal, including from intervals previously unrepresented, the basic predictions made in Glasspool and Scott (2010) remain unchanged. These data show that throughout the Middle Devonian charcoal occurrences were rare. This observation is supported by data from Kennedy et al. (2013) not included in the final analysis, the samples reported not being “coals”. These authors categorized two “coaly shales” from the Pragian and Emsian of New Brunswick, the former from the Val d’Amour Formation contained 0.8% inertinite, while the latter from the Campbellton Formation contained no inertinite. Had these data been included the former would have binned at 410 and 390 million years and the latter at 400 and 390 million years using the two binning intervals. The 15 million year 390 bin would have been little effected, its mean rising from 0.2% inert to 0.3 ± 0.2% inert (1 SE). However, the 10-million year binned data would have generated an earlier 410-million year bin of 0.8% inert and 400-million year bin of 0.1 ± 0.1% inert (1 SE). From the Middle Devonian to the Late Devonian there was a dramatic rise in wildfire occurrence within a 10-million year interval (see also Rimmer et al., 2015). From this point until the Early Triassic our data predict that p(O_2_) remained above the PAL.

From the latest Devonian–earliest Mississippian high p(O_2_) [the timing of this high is affected by the binning interval used (10 vs. 15 million year), but it is clear that p(O_2_) rose dramatically only in the last 20 million years of the Devonian, probably the last 10–15 million years] is predicted to have declined moderately but steadily throughout the Mississippian and Early–Middle Pennsylvanian before increasing rapidly from that point to a Phanerozoic high point in the middle to Late Cisuralian. However, Inert% predicts a bimodal p(O_2_) distribution in the Permian similar to previous modeling (Bergman et al., 2004) with a low point in the Guadalupian and a rebound in the Changhsingian. However, while these data indicate a Guadalupian decline in p(O_2_) they do not indicate hypoxia as a contributing factor in the end Guadalupian (∼260 Mya) mass extinction event (Retallack et al., 2006), as oxygen levels remained significantly above those experienced at present. Similarly, examination of Changhsingian (254.14–252.17 Mya) age coals indicates abundant charcoal and hence major wildfire activity within the last 2 million years of the Permian. This indicates that in the terrestrial realm p(O_2_) remained high despite widespread and persistent oceanic anoxia (‘superanoxia’) being reported in the Lopingian, with an onset ranging anywhere from the Early Wuchiapingian (Isozaki, 1997; Kato et al., 2002) to the Late Wuchiapingian or Early Changhsingian (Nielsen and Shen, 2004; Wignall et al., 2010; see also Wei et al., 2015). From these data, it also seems probable that p(O_2_) levels did not drive catastrophic terrestrial faunal diversity loss either during the Middle Permian (Capitanian) mass extinction event (Bond et al., 2015) or at the subsequent Permo-Triassic mass extinction event.

## Discussion

### Fire Vegetation and Climate in a High-Fire World

As has been discussed above, oxygen is a prerequisite for the propagation of fire and its level impacts flammability. The result of this is that when the oxygen level is under 16%, even during periods of Earth history where there are extensive dry seasons with large quantities of fuel to burn, there is unlikely to have been more than trivial wildfire activity (Belcher and McElwain, 2008). Equally, experiments have shown (Watson et al., 1978; Wildman et al., 2004) that as p(O_2_) rises wetter plants become liable to burn, and at levels >30% even plants and fuels with high-moisture contents would burn easily, even without a distinct dry season. Under these conditions fires would be widespread, frequent and catastrophic and could even proliferate in everwet ecosystems (Glasspool and Scott, 2010).

**FIGURE 3:**
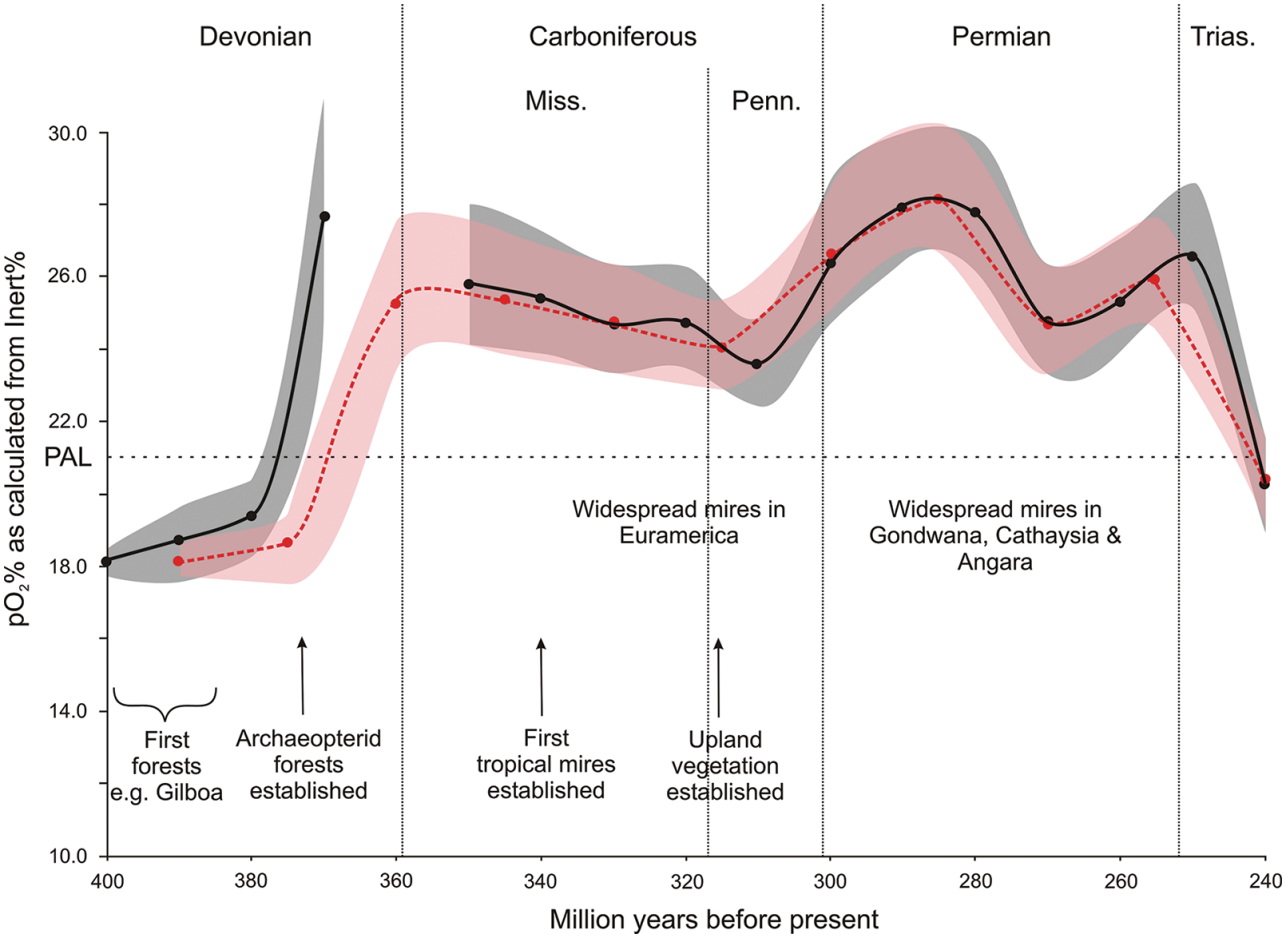
**The evolution of Late Paleozoic fire systems (based partly on data from Scott and Glasspool (2006).** The oxygen curves have been calculated from the inertinite in coal data (see Materials and Methods) and are based on 10 mllion year (solid black line) and 15 million year (dashed red line) binning of the data.

During the Late Paleozoic plants diversified greatly (Stewart and Rothwell, 1993; Taylor et al., 2009). As their growth forms, and range of growth environment evolved so too did the range of landscapes in which fire occurred (Scott and Glasspool, 2006). Of particular note, the authors observed that by the Carboniferous more potential fuel existed, especially through the development of extensive mires and upland vegetation, and that levels of p(O_2_) were elevated well above PAL, and that this combination would have led to the diversification of fire systems through this interval (**Figure 3**).

The nature of the growth, physiology and distribution of plants across these landscapes was not homogenous through the Late Paleozoic and this variation bears some discussion. In the Early Devonian early land plants were small and herbaceous, lacking both secondary tissues and macrophyllous leaves (Edwards, 1996). The reproductive strategies of these plants dictated their growth near to water courses and so their patchiness across the landscape (Algeo and Scheckler, 1998) would have meant they could not have supported extensive fires, although scattered records of charcoal do exist (e.g., Glasspool et al., 2006). The lack of any significant charcoal records in the Middle Devonian (Glasspool and Scott, 2010), despite the growth of the first forests at this time (Stein et al., 2007), has led to this interval being termed a “charcoal gap”, the existence of which has been correlated with low levels of p(O_2_) rather than an absence of fuel (Glasspool and Scott, 2010).

However, it was not until the development of extensive secondary tissues (wood in the progymnosperms and gymnosperms, secondary cortex in the lycopods), which allowed the evolution of trees and tree-like plants (Bateman et al., 1998; Meyer-Berthaud et al., 1999; Meyer-Berthaud and Decombeix, 2009) that the potential for extensive fuel loads developed. These fuels were perhaps for the first time both living and more than just recently senesced, their secondary tissues being more resistant to decay (Robinson, 1989, 1990, 1991; Boyce et al., 2010); however, wood-rotting fungi and bacteria are known and the arguments of Robinson can no longer be considered secure (see Rimmer et al., 2015). Significantly, “the worldwide appearance and rapid spread of *Archaeopteris* was complete” by the upper Frasnian (Scheckler, 2006) and is compatible with the timing of increased charcoal occurrence. Greater fuel build up combined with elevated p(O_2_) would greatly have promoted the potential for extensive fire events. In particular, later in the Carboniferous the rapid rate of growth of up to 50-m tall, 1-m diameter arborescent lycophytes in as little as 10 years with a plant density of 500–1800 plants per hectare (Cleal and Thomas, 2005) provides a huge potential, rapidly cycled, fuel load for combustion.

Plants from the Late Paleozoic onward can be considered: fire susceptible/sensitive; fire tolerant; fire resistant, or require fire. Interestingly, these characteristics are seen to develop through geological time. Differing approaches exist to unravel the relationships between plants and fire: (i) examination of the pattern of the evolution of different plants and their association with fire (Scott, 2000), (ii) examination of the evolution of traits linked to fire (Keeley et al., 2011b), and (iii) consideration of the relationship of modern plants with fire (Bond and van Wilgen, 1996) and their relationships as seen through cladistic analyses (Crisp et al., 2011; He et al., 2012).

Our understanding of fire traits is fraught with controversy (Keeley et al., 2011a). As pointed out by Keeley et al. (2011b) “No species is fire adapted but rather is adapted to a particular fire regime, which, among other things, includes fire frequency, fire intensity and patterns of fuel consumption.” However, a number of traits evolved by plants can be considered advantageous in a fire-prone ecosystem or biome. It is impossible to know from the fossil record whether or not a trait that is useful to a plant in a fire prone setting evolved because of an interaction with fire or simply that such a trait favored a plant in a fire-prone environment. For example, modern eucalypts are well-adapted to a high-frequency fire regime. It has been noted that these plants probably evolved near the transition from the Cretaceous to the Paleogene (Crisp et al., 2011) a time of high fire frequency (Bond and Scott, 2010; Glasspool and Scott, 2010) and that this may not be coincidental (Brown et al., 2012).

The clonal growth habit evolved in the Devonian (Bateman et al., 1998). In modern ecosystems, this trait allows plants to regrow after surface fires. This trait did not evolve as a response to fire but would have allowed plants with this growth form to take advantage of these events as a disturbance factor, e.g., during frequent surface fires of the Early Carboniferous (Scott, 2010; see also Robinson, 1989). Late Paleozoic sphenopsids had a variety of growth habits, from small creeping ground cover vegetation to tree-like forms that grew in thickets (Scott, 1978; Gastaldo, 1992). While the arborescent calamites may well have burned there is relatively little recognizable calamite charcoal. Vegetative reproduction in some ferns is common and is documented by organs such as *Kankakeea grundyi* in the Pennsylvanian (Pfefferkorn, 1973) and many ferns also exhibit clonal growth (Collinson et al., 2000; Collinson, 2001, 2002). They can thrive in disturbed environments, such as in volcanic landscapes and are also associated with fires (Scott and Galtier, 1985). Some of the oldest ferns in the Early Carboniferous are preserved as charcoal (Galtier and Scott, 1985; Scott and Galtier, 1985; Scott et al., 1985). This preservation may have related to volcanism, but some examples at least were charcoalified as a result of fire (Scott and Jones, 1994; Scott, 2010). Ferns with underground rhizomes are well placed to regenerate even if the above ground foliage is destroyed by fire (see for example Scott et al., 2000). Fire-fern relationships have also been reported for the Paleocene (Collinson et al., 2007), but this is less frequently considered in the Late Paleozoic (e.g., Glasspool, 2000; McParland et al., 2007).

Pteridosperms, or seed-ferns, originated in the latest Devonian and then diversified during the Early Carboniferous (Hilton and Bateman, 2006; Decombeix et al., 2011). They too are often found in disturbed settings preserved as charcoal (Scott et al., 1986, 2009; Rex and Scott, 1987). Glasspool (2000) reported the destruction of a glossopterid pteridosperm community as a result of a peat fire, where previous fire events had had little impact on the prevalence of these plants, suggesting that while they were fire tolerant major fire events still had the potential to negatively impact them. It is possible, regular low-intensity fires may have promoted the spread of certain glossopterids. Conversely, some liana-like plants appear to have been particularly susceptible to fire and periods of very high fire activity may have led to their extinction (Robinson, 1989). However, this seems unlikely given the prevalence of the gigantopterids, some of which were climbing plants and are interpreted to have been liana-like (see Seyfullah et al., 2014), during the Permian in Cathaysia an interval and locality with many heavily fire influenced coals.

Cordaites and conifers are frequently found as charcoal in the Late Paleozoic fossil record (Scott, 2000). The wood of cordaites is easily recognizable (Falcon-Lang and Scott, 2000) and even leaves have been found as charcoal (Scott and Collinson, 1978). During the Carboniferous, conifers diversified and spread into upland and extra-basinal environments. Many of the earliest known conifer remains occur as charcoal and demonstrate that fires occurred in these environments (Scott, 1974; Scott and Chaloner, 1983; Scott et al., 2010). The small needle-like leaves of these plants (e.g., Scott et al., 2010), with a large surface are to volume ratio, would have been particularly flammable (c.f. Belcher et al., 2010a). The shedding of lower branches in walchian conifers may also have been a response to frequent fires (Looy, 2013). As many early conifers are considered have grown in drier extra-basinal or even upland settings (Scott, 1974; Falcon-Lang et al., 2009; Scott et al., 2010), it is likely that these early conifer forests were more prone to fires that the better known vegetation thriving in lowland mire settings.

For the first time in the Late Carboniferous and Permian, a continuity of vegetation existed across the world. This combined with elevated p(O_2_) would have given rise to significant fire events across a range of biomes, especially in tropical and temperate mires (Scott and Glasspool, 2006). Were this the case, then fire would be expected to have played a role in the maintenance or change in vegetational structure (Bond and Midgley, 1995; Bond and Keeley, 2005; Bond et al., 2005; Bowman, 2005; Harrison et al., 2010).

Regular fires within open vegetation would have favored fast-growing, perhaps ‘weedy’, plants, particularly those with clonal growth that could tolerate low-temperature ground fires (Bond and Scott, 2010). In forested ecosystems, regular fires would have burned the floor litter and living surface vegetation without necessarily killing the forest trees (e.g., Glasspool, 2000). A build-up of fuel on the surface would have promoted more intense fires and may have initiated crown fires (Scott et al., 2014). This would have resulted in a more open vegetation pattern with a concomitant change in forest dynamics. Over short time scales, fluctuations in fire frequency and intensity would be reflected in the floral composition of successive beds, while over longer time scales the overall vegatational structure would be affected (Scott et al., 2014). Those working on modern fire systems have hypothesized on a super fire regime that incorporates concepts of a longer time scale and stability (Whitlock et al., 2010) and also the concept of pyromes (Archibald et al., 2013) that incorporates aspects of climate and rainfall, but these concepts have yet to be taken up by paleoecologists.

Fuel structure is an important element of fire propagation and spread (Scott et al., 2014). However, it is evident that vegetation and vegetation structure changed through the Late Paleozoic (DiMichele, 2014). The lowland vegetation of Euramerica has been reviewed in detail by DiMichele (2014), the differing plant groups and their differing growth habits and strategies. Most of the arborescent lycopods were cheaply constructed and grew very rapidly (Bateman and DiMichele, 1994; DiMichele, 2014). This rapid growth would potentially have facilitated survival of surface fires; in modern floras a tree height of 1 or 2 meters above ground level greatly reduces mortality (see Scott et al., 2014, for a review of this topic). Immature arborescent lycopsids often had long leaves that protected the growing apex of the plant. As the plant grew these leaves were shed and photosynthesis took place in the trunk surface (Phillips and DiMichele, 1992). Later, and depending on the taxon, the plant would branch (DiMichele, 2014). However, significantly there would have been a large gap between the ground and branched crown. This would have prevented the movement of fire up the trunk through extensive ladder fuels. Charred lycopsids leaves have rarely been reported, and it is possible that following dehiscence they were prevented from becoming fuel either by having been submerged or having rotted very quickly so that they did not form extensive fuel beds. Their needle-like form would otherwise have been highly flammable (see Belcher et al., 2010a). If the fire reached the crown then it is likely that all the leaves would have been fully combusted, leaving no charcoal residue. The evolution of thick bark layers would have afforded arborescent lycopsids significant protection against fire (Robinson, 1989, 1991; Falcon-Lang, 2000). However, the thick periderm of these plants once ignited would have been a significant fuel source and there is ample evidence of charred periderm in the fossil record (Falcon-Lang, 2000). Some charred branches are also reported from permineralized Pennsylvanian peats (DiMichele and Phillips, 1985).

Tree density and fuel connectivity are important considerations in the propagation of fire. An extreme example would be the Saguaro cactus forests of the Southwest United States, where a lightning strike may hit a cactus and cause it to catch fire, but the fire used not to spread due to a lack of surface fuel. In recent years foreign grasses have invaded this habitat and have provided fuel interconnectivity between cacti so that large areas of the vegetation may be destroyed in a single fire, as compared with a single cactus (Scott et al., 2014). As discussed above, during the Pennsylvanian Period peat-forming arborescent lycophytes with a diameter of about 1m grew at a density of between 500 and 1800 plants per hectare (Cleal and Thomas, 2005). Compared with mature angiosperm forests, this is a high tree density, though it’s noteworthy that arborescent lycophytes did not develop a canopy until maturity. However, this density may, in and of itself, have been sufficient to allow fire spread or it may have required additional fuel connectivity.

In parts of the forest floor ferns and pteridosperms were very common though they differed in both their growth strategies (DiMichele and Phillips, 2002; DiMichele et al., 2006) and presentation in the charcoal record. Many ferns were small ground-dwelling or scrambling climbing plants with small, thinly cuticularized, leaves (Phillips and Galtier, 2005, 2011). It is likely fire would have consumed these organs completely leaving a sparse fossil record. The axes of these ferns were more robust and charred examples appear commonly in the Mississippian (Scott, 2010) and can be seen frequently in Pennsylvanian coal ball assemblages from Illinois and Ohio (Glasspool pers. obs). However, many ferns were not small having developed a tree habit (DiMichele, 2014). While not extensively documented, the trunk root mantle of these plants can be found preserved as charcoal in many Late Paleozoic peats (Glasspool, pers. obs.).

The growth and nature of pteridosperms is very different to that of ferns. They produced larger leaves and pinnules with thicker cuticles (DiMichele, 2014), the fronds and fragments of fronds were readily shed and produced a significant litter (DiMichele et al., 2006). This may have facilitated the spread of surface fires. Pteridosperm pinnules and charred fragments are relatively common in a range of settings (Scott, 1978, 1984) including peat-forming environments where they may be the predominant group of plants found as charcoal (Scott, 2000, 2010; DiMichele et al., 2006). Climbing pteridosperms such as *Karinopteris*, *Pseudomariopteris*, and *Gigantonoclea hallei* were climbing plants (DiMichele et al., 1984; Krings and Kerp, 2000; Seyfullah et al., 2014). Such climbers may have acted as ladder fuels facilitating crown fires.

It has been suggested that the regular shedding of the branches of walchian conifers may have been an adaptation to fire, preventing the build-up of ladder fuels (Looy, 2013). However, this shedding would also have promoted more frequent surface fires. Similarly, while the southern hemisphere Permian Gondwanan glossopterids had a range of vegetative strategy, some having been small shrubs while others were large trees (Gould and Delevoryas, 1977; McLoughlin, 2012), all appear to have been deciduous. This characteristic would have built a more extensive litter. This in turn would probably have promoted regular surface fires but without resulting in tree mortality. However, as yet, no charred glossopterid leaves have been reported and most Permian charcoal appears to be from a range of gymnospermous trees (Jasper et al., 2013).

### Fire and the Earth System

As charcoal degrades much more slowly than uncharred wood (Ascough et al., 2011), there has been much recent discussion of using biochar to reduce present day atmospheric CO_2_ levels (Masek, 2013). This refractory phenomenon has been overlooked in deep time where intervals of frequent extensive fire may have had a similar potential to lock down atmospheric CO_2_.

Burning of vegetation in the short term increases the levels of CO_2_ in the atmosphere. However, in general this is balanced by the growth of plants, which takes up this CO_2_ (Lenton, 2013). On a slightly longer period, extensive regular forest combustion will modify the vegetation affecting plant productivity and stimulating global warming through charcoal burial and so CO_2_ draw-down. Extensive burning of peats would rapidly elevate atmospheric CO_2_ levels, a mechanism that has been proposed to explain the rapid temperature rise at the Paleocene–Eocene thermal maximum (PETM; Kurtz et al., 2003; Pancost et al., 2007), but which has never been suggested as a mechanism for global change in the Late Paleozoic. This is strange given the extent of peatlands in the Carboniferous and Permian. Climate drying, raised temperatures and peat cessation toward the end of the Permian could have led to regular and extensive peat fires across Gondwana, Cathaysia, and Angara that would have raised CO_2_ levels and contributed to the greenhouse effect. New evidence suggests that the ice caps melted before the end of the Permian (Rygel et al., 2008; **Figure 3**) and fire may have increased at that time. Shao et al. (2012) showed charcoal in coal levels in China rose through the latest Permian. Emphasis has been placed on the role of volcanicity and methane release, not on the burning of peats (albeit the effect of igneous intrusions in to the peat have been considered (see Benton and Newell, 2014 and references therein).

Various scenarios can be played out around this theme: for example if increased volcanism led to elevated atmospheric CO_2_ levels and the world warmed fire frequency would be expected to increase. This should result in increased charcoal burial, which would be expected to partially offset the CO_2_ level rise. However, plant productivity may decline and community structure change (e.g., Belcher et al., 2010a) again affecting fire systems and charcoal burial. In short, the feedback mechanisms are complex and need better analysis.

While there has been some consideration of charcoal occurrences on land there have been few studies on the contribution of charcoal to oceanic carbon (Smith et al., 1973; Goldberg, 1985). This is surprising given the importance of such a carbon sink in the modern oceans (Forbes et al., 2006). Indeed recent research suggests that remobilized charcoal is significant in reaching the modern ocean (Jaffe et al., 2013).

Vegetation and peat combustion produces smoke and aerosols. Increases in birth defects in the human population have been related to smoke emissions (Johnston et al., 2012), the same may be true for other animals regularly exposed to the effects of fire. However, smoke and aerosols have the potential to affect more than just the fauna, in modern tropical rainforests, aerosols from fires affect cloud formation and can prevent rain (Artaxo et al., 2009; Bowman et al., 2009). Further, fires may raise the levels of NO_x_ in the atmosphere, with plumes spreading into the upper atmosphere (Belcher, 2013; Scott et al., 2014). This mechanism has been little considered when compared with that from volcanoes (e.g., Benton and Newell, 2014).

Like today, the Earth during the Carboniferous and Permian was an icehouse world (Rygel et al., 2008). However, it is now thought that instead of there being a single icecap over the South Pole, there were several that waxed and waned. The effects of orbital cyclicity on Late Paleozoic ice melt and climate change are appreciated and have been discussed extensively (e.g., Jerrett et al., 2011). Meanwhile, the effects of fire on rates of ice melt have not been considered beyond the modern world, where there effect on albedo has been acknowledged (Bowman et al., 2009). This effect can be by blackening vegetation and in some cases changing green vegetation to bare soil. This may have only a short-term effect. However, there is also the effect of fine particulate carbon on snow. It has been shown in the recent icehouse that periods of high fire have coincided with large amounts of black carbon on ice and this has been linked to ice melting (Keegan et al., 2014). If sustained, for example in the southern hemisphere Permian, this would have played a role at least in the short-term contraction and expansion of the southern icecaps. This may have been more exaggerated if there were several smaller rather than one large icecap.

Fire may affect the movement of phosphorous both on land and in the oceans. This topic has been widely discussed (Kump, 1988; Lenton and Watson, 2000; Brown et al., 2012; Lenton, 2013), but not often taken fully into account when modeling the Late Paleozoic Earth system. Indeed, the impact of fire on the ocean system is not negligible. Carbon transport to the oceans is elevated by fires through the effects of post-fire erosion and transport (Jaffe et al., 2013). The organic carbon transported during such events includes both charcoal and un-charred plant matter. Large volumes of organic material can choke river systems [as seen in the Canadian Carboniferous (Falcon-Lang and Scott, 2000)] and make its way into the sea where it may be deposited in near-shore marine sediments (Nichols and Jones, 1992; Scott and Jones, 1994; Falcon-Lang, 1999, 2000; Scott, 2000) but may also be transported out into deeper marine settings (Scott, 2000). However, the volumes of finer black carbon may be large, as in the recent oceans (Smith et al., 1973; Herring, 1985; Forbes et al., 2006). A combination of large amounts of plant material entering the ocean together with enhanced phosphorus content may lead, or at least amplify, ocean anoxia. There have been few studies on the impact of fire in the Late Permian to the widespread anoxia observed in the oceans at this time.

A widely recognized relationship exists between fire, climate and atmosphere (Bowman et al., 2009). Changes in fire frequency and extent play a part in the regulation of atmospheric gasses (Turquety, 2013) but also impact climate (Beerling et al., 1998). Models of the Earth system in the Carboniferous and Permian are beginning to take this in to account (e.g., Beerling et al., 1998, 2002). The Permian-Triassic mass extinction event has been extensively studied (Benton and Newell, 2014). Climate warming is predicted leading up to this event (Benton, 2003; Benton and Newell, 2014), with an ensuing loss of floral ecosystem health. This event would have changed the vegetation structure, with less interconnectivity between plants. This in turn would have made fire spread more difficult. However, were vegetation mortality rising due to rising levels of NO_x_ from volcanic activity then dry fuel should have become more abundant and fire activity should have spiked along with an associated rise in run-off and erosion. Markers suggesting increased wildfire activity have been reported at the Permian-Triassic boundary in China (Shen et al., 2011), but whether this is a global signal remains to be demonstrated. However, while not mentioning fire, massive erosion at the Permian-Triassic boundary has been suggested (Benton and Newell, 2014). Perhaps the role of fire at the boundary, clearly from the data presented herein not a time of low p(O_2_), was greater than has currently been appreciated?

## Conclusion

New data from Kennedy et al. (2013) support the concept of a Middle Devonian “charcoal gap”, but notably hint at higher levels of fire activity during earliest Devonian. Increased fire activity during the latest Silurian to earliest Devonian is in accord with predictions made by Scott and Glasspool (2006) and would fit with elevated levels of p(O_2_) during that interval predicted by Berner (2006).

Data from charcoal abundance in coal indicate a dramatic rise in p(O_2_) levels during the last 10–15 million years of the Devonian, atmospheric oxygen concentration then remained above present day levels, and usually above 23%, until at least end Permian. During this time, fires would have profoundly affected the Earth system, impacting the vegetation and the fauna as well as the carbon, oxygen and even phosphorous cycles. The Late Paleozoic at this time can be characterized as a ‘high-fire’ world, where fires were promoted by elevated levels of p(O_2_) and an ecologically and physiologically diverse vegetation capable of acting as a major and extensive fuel resource.

Levels of p(O_2_) appear to have peaked in the Middle-to-Late Cisuralian at levels of about 28%, before declining modestly into the Guadalupian and then recovering again in the Lopingian. Despite this bimodal distribution in the Permian, p(O_2_) does not appear to have declined to levels that would have induced hypoxia either during the Guadalupian or the latest Changhsingian, despite the predicted onset of widespread and persistent oceanic anoxia in the Lopingian (Wei et al., 2015).

The direct impacts of fire on the Late Paleozoic world are numerous and are largely apparent, e.g., ecosystems subjected to frequent fires, more run-off and erosion following fire, particularly in areas of elevated topography leading to more disturbed environments. However, fires would also have had more subtle and indirect feedbacks. These feedbacks have impacted the Earth system over varied durations, from the short term to some effects that are still being felt today: the exploitation of many Permian charcoal-rich coals is still a major part of the economies of the world’s two most populous nations.

## Conflict of Interest Statement

The authors declare that the research was conducted in the absence of any commercial or financial relationships that could be construed as a potential conflict of interest.
