# Supplementary material for: The impact of fire on the Late Paleozoic Earth system
Source: Front Plant Sci. 2015 Sep 23;6:756. doi: 10.3389/fpls.2015.00756 (PMC4585212; doi:10.3389/fpls.2015.00756)
Supplement: Supplementary file 1 [file Table_1.PDF]

Reference	Age	Locality	Stratigraphy	Notes	No. samples	Average inertinite mmf %	Ma	10 myr bin	15 myr bin
Data from: Glasspool and Scott, 2010	Ladinian-Carnian, 242 to 227 mya	Australia, Jupiter 1 Well	Mungaroo Fm Pro-delta		1	8.0	234.5	230	240
Data from: Glasspool and Scott, 2010	Julian, 236.6 to 234.45 mya	Austria	Lunze Decke	Kleinzell	1	3.0	235.5	240	240
Data from: Glasspool and Scott, 2010	Julian, 236.6 to 234.45 mya	Austria	Lunze Decke	Hallbachtal	1	0.0	235.5	240	240
Data from: Glasspool and Scott, 2010	Julian, 236.6 to 234.45 mya	Austria	Lunze Decke	Wobachtal	1	0.0	235.5	240	240
Data from: Glasspool and Scott, 2010	Julian, 236.6 to 234.45 mya	Austria	Lunze Decke	Meierhofstollen	1	1.0	235.5	240	240
Data from: Glasspool and Scott, 2010	Julian, 236.6 to 234.45 mya	Austria	Lunze Decke	Annastollen	1	3.0	235.5	240	240
Data from: Glasspool and Scott, 2010	Julian, 236.6 to 234.45 mya	Austria	Lunze Decke	Neukarolusstollen	1	0.0	235.5	240	240
Data from: Glasspool and Scott, 2010	Julian, 236.6 to 234.45 mya	Austria	Lunze Decke	Mittereck	1	0.0	235.5	240	240
Data from: Glasspool and Scott, 2010	Julian, 236.6 to 234.45 mya	Austria	Lunze Decke	Korngrub	1	3.0	235.5	240	240
Data from: Glasspool and Scott, 2010	Julian, 236.6 to 234.45 mya	Austria	Lunze Decke	Unterbuchberg	1	1.0	235.5	240	240
Data from: Glasspool and Scott, 2010	Julian, 236.6 to 234.45 mya	Austria	Lunze Decke	Bissiger Hundst.	1	0.0	235.5	240	240
Data from: Glasspool and Scott, 2010	Julian, 236.6 to 234.45 mya	Austria	Lunze Decke	Wenigsthof	1	0.0	235.5	240	240
Data from: Glasspool and Scott, 2010	Julian, 236.6 to 234.45 mya	Austria	Lunze Decke	Prinzbach	1	0.0	235.5	240	240
Data from: Glasspool and Scott, 2010	Julian, 236.6 to 234.45 mya	Austria	Lunze Decke	Schindeleck	1	1.0	235.5	240	240
Data from: Glasspool and Scott, 2010	Julian, 236.6 to 234.45 mya	Austria	Lunze Decke	Rehgraben	1	0.0	235.5	240	240
Data from: Glasspool and Scott, 2010	Julian, 236.6 to 234.45 mya	Austria	Lunze Decke	Loichgraben	1	0.0	235.5	240	240
Data from: Glasspool and Scott, 2010	Julian, 236.6 to 234.45 mya	Austria	Lunze Decke	Guttenhofgegend	1	0.0	235.5	240	240
Data from: Glasspool and Scott, 2010	Julian, 236.6 to 234.45 mya	Austria	Lunze Decke	Loich	1	5.0	235.5	240	240
Data from: Glasspool and Scott, 2010	Julian, 236.6 to 234.45 mya	Austria	Lunze Decke	Oberklaus	1	0.0	235.5	240	240
Data from: Glasspool and Scott, 2010	Julian, 236.6 to 234.45 mya	Austria	Lunze Decke	Kögerl/St. Anton	1	0.0	235.5	240	240
Data from: Glasspool and Scott, 2010	Julian, 236.6 to 234.45 mya	Austria	Lunze Decke	Bichlweber	1	1.0	235.5	240	240
Data from: Glasspool and Scott, 2010	Julian, 236.6 to 234.45 mya	Austria	Lunze Decke	Polzberg	1	1.0	235.5	240	240
Data from: Glasspool and Scott, 2010	Julian, 236.6 to 234.45 mya	Austria	Lunze Decke	Gaming Halde	1	2.0	235.5	240	240
Data from: Glasspool and Scott, 2010	Julian, 236.6 to 234.45 mya	Austria	Lunze Decke	Gaming	1	1.0	235.5	240	240

Data from: Glasspool and Scott, 2010	Julian, 236.6 to 234.45 mya	Austria	Lunze Decke	Gaming	1	2.0	235.5	240	240
Data from: Glasspool and Scott, 2010	Julian, 236.6 to 234.45 mya	Austria	Lunze Decke	Gaming	1	0.0	235.5	240	240
Data from: Glasspool and Scott, 2010	Julian, 236.6 to 234.45 mya	Austria	Lunze Decke	Zürner	1	0.0	235.5	240	240
Data from: Glasspool and Scott, 2010	Julian, 236.6 to 234.45 mya	Austria	Lunze Decke	Moosau	1	0.0	235.5	240	240
Data from: Glasspool and Scott, 2010	Julian, 236.6 to 234.45 mya	Austria	Lunze Decke	Schneibb	1	2.0	235.5	240	240
Data from: Glasspool and Scott, 2010	Julian, 236.6 to 234.45 mya	Austria	Lunze Decke	Weyer/Mühlein	1	3.0	235.5	240	240
Data from: Glasspool and Scott, 2010	Julian, 236.6 to 234.45 mya	Austria	Lunze Decke	Linda	1	0.0	235.5	240	240
Data from: Glasspool and Scott, 2010 (Age: Holmes and Anderson, 2013)	Ladinian, 242 to 237 mya	Australia, Clarence-Moreton Basin	Nymboida Coal Measures, Basin Creek Fm, Farquhars Seam		1	14.9	239.5	240	240
Smyth, 1972	Anisian-Ladinian, 247.2 to 237 mya	South Australia, Pedirka Basin, Poolowanna No. 1 Well	Walkandi Fm	Table 6.5: 9181ft	1	27.0	242.1	240	240
Holdgate et al., 2005: Age: Slater et al., 2015	Changhsingian, 254.14 to 252.17 mya	Antarctica, Lambert Graben	Bainmedart Coal Measures, McKinnon Mbr, 95/14		1	35.2	253.2	250	255
Holdgate et al., 2005: Age: Slater et al., 2015	Changhsingian, 254.14 to 252.17 mya	Antarctica, Lambert Graben	Bainmedart Coal Measures, McKinnon Mbr, 95/15		1	31.4	253.2	250	255
Holdgate et al., 2005: Age: Slater et al., 2015	Changhsingian, 254.14 to 252.17 mya	Antarctica, Lambert Graben	Bainmedart Coal Measures, McKinnon Mbr, 95/9		1	45.9	253.2	250	255
Data from: Glasspool and Scott, 2010	Changhsingian, 254.14 to 252.17 mya	Australia	Baralaba Coal Measures, Baralaba		1	31.9	253.2	250	255
Data from: Glasspool and Scott, 2010	Changhsingian, 254.14 to 252.17 mya	Australia	Baralaba Coal Measures, Moura		1	18.8	253.2	250	255
Data from: Glasspool and Scott, 2010	Changhsingian, 254.14 to 252.17 mya	Australia	Illawarra Coal Measures, Bulli and Balgownie		1	52.1	253.2	250	255
Data from: Glasspool and Scott, 2010 + Huleatt, 1991	Changhsingian, 254.14 to 252.17 mya	Australia	Illawarra Coal Measures, Wongawilli	((23.9*10)+(16.7+23.0 +26.1))/13	13	23.4	253.2	250	255
Data from: Glasspool and Scott, 2010	Changhsingian, 254.14 to 252.17 mya	Australia	Rangal Coal Measures, Blackwater Aries		1	45.8	253.2	250	255
Data from: Glasspool and Scott, 2010	Changhsingian, 254.14 to 252.17 mya	Australia	Rangal Coal Measures, Blackwater Pollux		1	42.5	253.2	250	255
Data from: Glasspool and Scott, 2010	Changhsingian, 254.14 to 252.17 mya	Australia	Rangal Coal Measures, Utah Blackwater		1	46.3	253.2	250	255
Data from: Glasspool and Scott, 2010	Changhsingian, 254.14 to 252.17 mya	Australia	Rangal Coal Measures, Yarrabee	Yarrabee	1	29.2	253.2	250	255
Data from: Glasspool and Scott, 2010	Changhsingian, 254.14 to 252.17 mya	Australia, Bowen Basin	Rangal Coal Measures	Unclear how many seams	23	49.1	253.2	250	255
Data from: Glasspool and Scott, 2010	Changhsingian, 254.14 to 252.17 mya	Australia, Bowen Basin	Rangal Coal Measures, Orion Seam		2	31.4	253.2	250	255
Huleatt, 1991	Changhsingian, 254.14 to 252.17 mya	Australia, Bowen Basin, Baralaba	Baralaba Coal Measures, unspecified seam		1	31.9	253.2	250	255

Huleatt, 1991	Changhsingian, 254.14 to 252.17 mya	Australia, Bowen Basin, Blackwater	Rangal Coal Measures, Aries	Aries	1	45.8	253.2	250	255
Huleatt, 1991	Changhsingian, 254.14 to 252.17 mya	Australia, Bowen Basin, Blackwater	Rangal Coal Measures, Aries I	Aries I	2	36.8	253.2	250	255
Huleatt, 1991	Changhsingian, 254.14 to 252.17 mya	Australia, Bowen Basin, Blackwater	Rangal Coal Measures, Aries II	Aries II	3	63.3	253.2	250	255
Huleatt, 1991	Changhsingian, 254.14 to 252.17 mya	Australia, Bowen Basin, Blackwater	Rangal Coal Measures, Aries II Upper	Aries II Upper	3	48.0	253.2	250	255
Huleatt, 1991	Changhsingian, 254.14 to 252.17 mya	Australia, Bowen Basin, Blackwater	Rangal Coal Measures, Aries-Castor	Aries-Castor	3	62.4	253.2	250	255
Huleatt, 1991	Changhsingian, 254.14 to 252.17 mya	Australia, Bowen Basin, Blackwater	Rangal Coal Measures, Castor Lower	Castor Lower	3	48.4	253.2	250	255
Data from: Glasspool and Scott, 2010 + Huleatt, 1991	Changhsingian, 254.14 to 252.17 mya	Australia, Bowen Basin, Blackwater	Rangal Coal Measures, Castor Seam	((44.4*2)+(60.3*3))/5	5	53.9	253.2	250	255
Huleatt, 1991	Changhsingian, 254.14 to 252.17 mya	Australia, Bowen Basin, Blackwater	Rangal Coal Measures, Castor Upper	Castor Upper	2	62.5	253.2	250	255
Huleatt, 1991	Changhsingian, 254.14 to 252.17 mya	Australia, Bowen Basin, Blackwater	Rangal Coal Measures, Gemini	Gemini	1	65.6	253.2	250	255
Huleatt, 1991	Changhsingian, 254.14 to 252.17 mya	Australia, Bowen Basin, Blackwater	Rangal Coal Measures, Mammoth	Mammoth	2	47.0	253.2	250	255
Huleatt, 1991	Changhsingian, 254.14 to 252.17 mya	Australia, Bowen Basin, Blackwater	Rangal Coal Measures, Pollux Seam	Pollux	3	46.7	253.2	250	255
Data from: Glasspool and Scott, 2010 + Huleatt, 1991	Changhsingian, 254.14 to 252.17 mya	Australia, Bowen Basin, Mackay	Rangal Coal Measures, Elphinstone	((57.9*1)+(57.4*1))/2	2	57.7	253.2	250	255
Data from: Glasspool and Scott, 2010 + Huleatt, 1991	Changhsingian, 254.14 to 252.17 mya	Australia, Bowen Basin, Mackay	Rangal Coal Measures, Hynds		1	48.9	253.2	250	255
Huleatt, 1991	Changhsingian, 254.14 to 252.17 mya	Australia, Bowen Basin, Mackay	Rangal Coal Measures, Leichhardt Seam	Leichhardt	3	67.6	253.2	250	255
Data from: Glasspool and Scott, 2010 + Huleatt, 1991	Changhsingian, 254.14 to 252.17 mya	Australia, Bowen Basin, Mackay	Rangal Coal Measures, Upper Newlands Seam	((33.7*1)+(58.5*1))/2	2	46.1	253.2	250	255
Huleatt, 1991	Changhsingian, 254.14 to 252.17 mya	Australia, Bowen Basin, Mackay	Rangal Coal Measures, Vermont Seam	Vermont	3	76.4	253.2	250	255
Huleatt, 1991	Changhsingian, 254.14 to 252.17 mya	Australia, Bowen Basin, Moura	Baralaba Coal Measures, unspecified seam		1	19.8	253.2	250	255
Huleatt, 1991	Changhsingian, 254.14 to 252.17 mya	Australia, Poitrel Mine	Rangal Coal Measures, Leichart and Vermont Thermal coals		1	54.9	253.2	250	255
Data from: Glasspool and Scott, 2010 + Huleatt, 1991	Changhsingian, 254.14 to 252.17 mya	Australia, Sydney Basin, Newcastle Coalfield	Newcastle Coal Measures (Upper), Moon Island Beach Formation, Great Northern	((46.3*4)+(35.8+43.2+51.0))/7	7	45.0	253.2	250	255
Data from: Glasspool and Scott, 2010 + Huleatt, 1991	Changhsingian, 254.14 to 252.17 mya	Australia, Sydney Basin, Newcastle Coalfield	Newcastle Coal Measures (Upper), Moon Island Beach Formation, Wallarah	(47.4+51.1+55.8)/3	3	51.4	253.2	250	255
Huleatt, 1991	Changhsingian, 254.14 to 252.17 mya	Australia, Sydney Basin, Newcastle Coalfield	Newcastle Coal Measures, Borehole Seam	Borehole	3	15.3	253.2	250	255
Huleatt, 1991	Changhsingian, 254.14 to 252.17 mya	Australia, Sydney Basin, Newcastle Coalfield	Newcastle Coal Measures, Dudley	Dudley	3	17.1	253.2	250	255
Huleatt, 1991	Changhsingian, 254.14 to 252.17 mya	Australia, Sydney Basin, Newcastle Coalfield	Newcastle Coal Measures, Fassifern	Fassifern	1	32.6	253.2	250	255

Huleatt, 1991	Changhsingian, 254.14 to 252.17 mya	Australia, Sydney Basin, Newcastle Coalfield	Newcastle Coal Measures, Nobbys	Nobbys	1	10.6	253.2	250	255
Huleatt, 1991	Changhsingian, 254.14 to 252.17 mya	Australia, Sydney Basin, Newcastle Coalfield	Newcastle Coal Measures, Victoria Tunnel	Victoria Tunnel	3	10.0	253.2	250	255
Huleatt, 1991	Changhsingian, 254.14 to 252.17 mya	Australia, Sydney Basin, Newcastle Coalfield	Newcastle Coal Measures, Wave Hill	Wave Hill	1	8.6	253.2	250	255
Huleatt, 1991	Changhsingian, 254.14 to 252.17 mya	Australia, Sydney Basin, Newcastle Coalfield	Newcastle Coal Measures, Yard	Yard	1	15.5	253.2	250	255
Data from: Glasspool and Scott, 2010 + Huleatt, 1991	Changhsingian, 254.14 to 252.17 mya	Australia, Sydney Basin, Southern Coalfield	Illawarra Coal Measures, Balgownie Seam	(36.5 +43.6)/2	2	40.1	253.2	250	255
Data from: Glasspool and Scott, 2010 + Huleatt, 1991	Changhsingian, 254.14 to 252.17 mya	Australia, Sydney Basin, Southern Coalfield	Illawarra Coal Measures, Bulli Seam	((52.9*24)+(38.5+39.4 +39.4))/27	27	51.4	253.2	250	255
Huleatt, 1991	Changhsingian, 254.14 to 252.17 mya	Australia, Sydney Basin, Southern Coalfield, Burragorang Valley	Illawarra Coal Measures, Bulli Seam	Bulli	3	46.0	253.2	250	255
Huleatt, 1991	Changhsingian, 254.14 to 252.17 mya	Australia, Sydney Basin, Western Coalfield	Illawarra Coal Measures, Katoomba	Katoomba	1	66.3	253.2	250	255
Data from: Glasspool and Scott, 2010	Changhsingian, 254.14 to 252.17 mya	Australia, Theodore North Mine	Baralaba Coal Measures, (Averaged coal)		1	31.8	253.2	250	255
Data from: Glasspool and Scott, 2010	Changhsingian, 254.14 to 252.17 mya	Australia, Theodore South Mine	Baralaba Coal Measures, 3/4 Seam		1	35.1	253.2	250	255
New data from: Shao, this paper	Changhsingian, 254.14 to 252.17 mya	China, Eastern Yunnan Province, Taoshuping of Fuyuan County	Upper Xuanwei Fm, Seam K2	Upper Xuanwei Fm, Seam K2	3	34.9	253.2	250	255
New data from: Shao, this paper	Changhsingian, 254.14 to 252.17 mya	China, Eastern Yunnan Province, Taoshuping of Fuyuan County	Upper Xuanwei Fm, Seam K3	Upper Xuanwei Fm, Seam K3	13	24.7	253.2	250	255
New data from: Shao, this paper	Changhsingian, 254.14 to 252.17 mya	China, Eastern Yunnan Province, Taoshuping of Fuyuan County	Upper Xuanwei Fm, Seam K6	Upper Xuanwei Fm, Seam K6	3	29.4	253.2	250	255
Dai, et al., 2013	Changhsingian, 254.14 to 252.17 mya	China, Eastern Yunnan Province, Xinde Coal Mine	Xuanwei Fm., Seam C2	Xuanwei Fm., Seam C2	3	34.7	253.2	250	255
Dai, et al., 2013	Changhsingian, 254.14 to 252.17 mya	China, Eastern Yunnan Province, Xinde Coal Mine	Xuanwei Fm., Seam C3	Xuanwei Fm., Seam C3	3	25.7	253.2	250	255
New data from: Shao, this paper	Changhsingian, 254.14 to 252.17 mya	China, Eastern Yunnan Province, Yangtan of Xuanwei County	Upper Xuanwei Fm, Seam B2	Upper Xuanwei Fm, Seam B2	19	31.2	253.2	250	255
New data from: Shao, this paper	Changhsingian, 254.14 to 252.17 mya	China, Eastern Yunnan Province, Yangtan of Xuanwei County	Upper Xuanwei Fm, Seam B3	Upper Xuanwei Fm, Seam B3	12	33.4	253.2	250	255
New data from: Shao, this paper	Changhsingian, 254.14 to 252.17 mya	China, Eastern Yunnan Province, Yujinshan of Weixin County	Changhsing Fm, Seam C1	Changhsing Fm, Seam C1	6	39.3	253.2	250	255
Data from: Glasspool and Scott, 2010	Changhsingian, 254.14 to 252.17 mya	China, SW of South China	C3	C3	1	14.0	253.2	250	255
Data from: Glasspool and Scott, 2010	Changhsingian, 254.14 to 252.17 mya	China, SW of South China	C409	C409	1	30.7	253.2	250	255
Data from: Glasspool and Scott, 2010	Changhsingian, 254.14 to 252.17 mya	China, SW of South China	C5	C5	1	23.2	253.2	250	255
Data from: Glasspool and Scott, 2010	Changhsingian, 254.14 to 252.17 mya	China, Zhijin Coalfield	Seam # 5	Seam # 5	1	39.6	253.2	250	255

Holdgate et al., 2005: Age: Slater et al., 2015	Wuchiapingian, 259.8 to 254.14 mya	Antarctica, Lambert Graben	Bainmedart Coal Measures, McKinnon Mbr, 95/21		1	50.5	257.0	260	255
Holdgate et al., 2005: Age: Slater et al., 2015	Wuchiapingian, 259.8 to 254.14 mya	Antarctica, Lambert Graben	Bainmedart Coal Measures, McKinnon Mbr, 95/33		1	46.7	257.0	260	255
Data from: Glasspool and Scott, 2010	Wuchiapingian, 259.8 to 254.14 mya	Australia	Charbon Subgroup, Newnes-Longswamp Fm, Wolgan/Irondale		1	22.1	257.0	260	255
Data from: Glasspool and Scott, 2010 + Huleatt, 1991	Wuchiapingian, 259.8 to 254.14 mya	Australia	Illawarra Coal Measures, Wilton Formation, Tongarra Seam	(44.0 + 22.1)/2	2	33.1	257.0	260	255
Data from: Glasspool and Scott, 2010	Wuchiapingian, 259.8 to 254.14 mya	Australia	Malabar Formation, Westfalen/Redbank		2	26.2	257.0	260	255
Data from: Glasspool and Scott, 2010	Wuchiapingian, 259.8 to 254.14 mya	Australia	Moranbah Coal Measures, Dysart Rider		1	16.1	257.0	260	255
Data from: Glasspool and Scott, 2010	Wuchiapingian, 259.8 to 254.14 mya	Australia	Moranbah Coal Measures, Goonyella		1	44.2	257.0	260	255
Data from: Glasspool and Scott, 2010	Wuchiapingian, 259.8 to 254.14 mya	Australia	Moranbah Coal Measures, North Ipswich		1	14.0	257.0	260	255
Data from: Glasspool and Scott, 2010	Wuchiapingian, 259.8 to 254.14 mya	Australia	Moranbah Coal Measures, Peak Downs		1	25.3	257.0	260	255
Data from: Glasspool and Scott, 2010	Wuchiapingian, 259.8 to 254.14 mya	Australia	Moranbah Coal Measures, Riverside		1	37.2	257.0	260	255
Data from: Glasspool and Scott, 2010 + Huleatt, 1991	Wuchiapingian, 259.8 to 254.14 mya	Australia, Bowen Basin	Moranbah Coal Measures, Dysart Seam	((23.2*1)+(31.6*2))/3	3	28.8	257.0	260	255
Data from: Glasspool and Scott, 2010 + Huleatt, 1991	Wuchiapingian, 259.8 to 254.14 mya	Australia, Bowen Basin	Moranbah Coal Measures, Goonyella Lower Seam	((51.5*2)+(39.7*2))/4	4	45.6	257.0	260	255
Data from: Glasspool and Scott, 2010 + Huleatt, 1991	Wuchiapingian, 259.8 to 254.14 mya	Australia, Bowen Basin	Moranbah Coal Measures, Harrow Creek Seam	((30.2*1)+(28.4*3))/4	4	28.9	257.0	260	255
Data from: Glasspool and Scott, 2010 + Huleatt, 1991	Wuchiapingian, 259.8 to 254.14 mya	Australia, Bowen Basin	Moranbah Coal Measures, P Seam	((23.2*1)+(23.3*3))/4	4	23.3	257.0	260	255
Huleatt, 1991	Wuchiapingian, 259.8 to 254.14 mya	Australia, Bowen Basin, Blackwater	German Creek Fm, German Creek Seam		1	27.1	257.0	260	255
Huleatt, 1991	Wuchiapingian, 259.8 to 254.14 mya	Australia, Bowen Basin, Blackwater	German Creek Fm, Lilyvale Seam		3	23.3	257.0	260	255
Huleatt, 1991	Wuchiapingian, 259.8 to 254.14 mya	Australia, Bowen Basin, Mackay	Moranbah Coal Measures, Goonyella Middle Seam		1	49.5	257.0	260	255
Huleatt, 1991	Wuchiapingian, 259.8 to 254.14 mya	Australia, Bowen Basin, Mackay	Moranbah Coal Measures, Q Seam		3	25.3	257.0	260	255
Data from: Glasspool and Scott, 2010	Wuchiapingian, 259.8 to 254.14 mya	Australia, Goonyella, Riverside, German Creek Mines	Moranbah Coal Measures	Unclear how many seams	17	27.4	257.0	260	255
Data from: Glasspool and Scott, 2010 + Huleatt, 1991	Wuchiapingian, 259.8 to 254.14 mya	Australia, Gunnedah Basin, Gunnedah Coalfield	Black Jack Fm, Coogal Subgroup, Hoskissons Seam	((34*1)+(26.3+29.5+30.2))/4	4	30.0	257.0	260	255
Data from: Glasspool and Scott, 2010	Wuchiapingian, 259.8 to 254.14 mya	Australia, Hunter Valley	Lower Whybrow Seam		1	36.7	257.0	260	255
Data from: Glasspool and Scott, 2010	Wuchiapingian, 259.8 to 254.14 mya	Australia, Peak Downs Mine	Moranbah Coal Measures, Blended Harrow and Dysart Seams		1	24.2	257.0	260	255

Data from: Glasspool and Scott, 2010	Wuchiapingian, 259.8 to 254.14 mya	Australia, Saraji Mine	Moranbah Coal Measures, Blended Dysart J and Harrow Creek Lower Seams		1	24.2	257.0	260	255
Data from: Glasspool and Scott, 2010 + Huleatt, 1991	Wuchiapingian, 259.8 to 254.14 mya	Australia, Sydney Basin, Hunter Valley	Wittingham Coal Measures, Jerry's Plain Subgroup, Malabar Formation, Wambo Seam	(16.8 + 20.8)/2	2	18.8	257.0	260	255
Data from: Glasspool and Scott, 2010 + Huleatt, 1991	Wuchiapingian, 259.8 to 254.14 mya	Australia, Sydney Basin, Hunter Valley	Wittingham Coal Measures, Jerry's Plain Subgroup, Mount Leonard Formation, Whybrow	(12.4 + 11.5)/2	2	12.0	257.0	260	255
Data from: Glasspool and Scott, 2010	Wuchiapingian, 259.8 to 254.14 mya	China, Chongqing mining district, Moxinpo Mine	Longtan Fm, K2		1	12.0	257.0	260	255
Data from: Glasspool and Scott, 2010	Wuchiapingian, 259.8 to 254.14 mya	China, Chongqing mining district, Moxinpo Mine	Longtan Fm, K4		1	13.0	257.0	260	255
Data from: Glasspool and Scott, 2010	Wuchiapingian, 259.8 to 254.14 mya	China, Chongqing mining district, Moxinpo Mine	Longtan Fm, K5		1	15.0	257.0	260	255
Data from: Glasspool and Scott, 2010	Wuchiapingian, 259.8 to 254.14 mya	China, Chongqing mining district, Moxinpo Mine	Longtan Fm, K7		1	9.9	257.0	260	255
Data from: Glasspool and Scott, 2010	Wuchiapingian, 259.8 to 254.14 mya	China, Chongqing mining district, Zhongliangshan Mine	Longtan Fm, K1a		1	14.7	257.0	260	255
Data from: Glasspool and Scott, 2010	Wuchiapingian, 259.8 to 254.14 mya	China, Chongqing mining district, Zhongliangshan Mine	Longtan Fm, K1b		1	8.1	257.0	260	255
Data from: Glasspool and Scott, 2010	Wuchiapingian, 259.8 to 254.14 mya	China, Dafang Coalfield	Seam # 11		1	10.9	257.0	260	255
Data from: Glasspool and Scott, 2010	Wuchiapingian, 259.8 to 254.14 mya	China, Dafang Coalfield	Seam # 3		1	20.4	257.0	260	255
New data from: Shao, this paper	Wuchiapingian, 259.8 to 254.14 mya	China, Eastern Yunnan Province, Taoshuping of Fuyuan County	Lower Xuanwei Fm, Seam K21		2	23.4	257.0	260	255
New data from: Shao, this paper	Wuchiapingian, 259.8 to 254.14 mya	China, Eastern Yunnan Province, Taoshuping of Fuyuan County	Lower Xuanwei Fm, Seam K22		4	37.0	257.0	260	255
New data from: Shao, this paper	Wuchiapingian, 259.8 to 254.14 mya	China, Eastern Yunnan Province, Taoshuping of Fuyuan County	Middle Xuanwei Fm, Seam K15		15	29.1	257.0	260	255
New data from: Shao, this paper	Wuchiapingian, 259.8 to 254.14 mya	China, Eastern Yunnan Province, Yujinshan of Weixin County	Wujiaping Fm, Seam C5		20	24.6	257.0	260	255
New data from: Shao, this paper	Wuchiapingian, 259.8 to 254.14 mya	China, Eastern Yunnan Province, Yujinshan of Weixin County	Wujiaping Fm, Seam C56		3	25.1	257.0	260	255
New data from: Shao, this paper	Wuchiapingian, 259.8 to 254.14 mya	China, Eastern Yunnan Province, Yujinshan of Weixin County	Wujiaping Fm, Seam C6		4	49.0	257.0	260	255
New data from: Shao, this paper	Wuchiapingian, 259.8 to 254.14 mya	China, Eastern Yunnan Province, Yujinshan of Weixin County	Wujiaping Fm, Seam C7		4	34.1	257.0	260	255
Data from: Glasspool and Scott, 2010	Wuchiapingian, 259.8 to 254.14 mya	China, Guangxi, Heshan Coalfield	Heshan Fm, Seam 3A		4	18.9	257.0	260	255
Data from: Glasspool and Scott, 2010	Wuchiapingian, 259.8 to 254.14 mya	China, Guangxi, Heshan Coalfield	Heshan Fm, Seam 3B		1	25.3	257.0	260	255

Data from: Glasspool and Scott, 2010	Wuchiapingian, 259.8 to 254.14 mya	China, Guangxi, Heshan Coalfield	Heshan Fm, Seam 3C		3	19.4	257.0	260	255
Data from: Glasspool and Scott, 2010	Wuchiapingian, 259.8 to 254.14 mya	China, Guangxi, Heshan Coalfield	Heshan Fm, Seam 4A		1	30.5	257.0	260	255
Data from: Glasspool and Scott, 2010	Wuchiapingian, 259.8 to 254.14 mya	China, Guangxi, Heshan Coalfield	Heshan Fm, Seam 4B		3	15.0	257.0	260	255
Data from: Glasspool and Scott, 2010	Wuchiapingian, 259.8 to 254.14 mya	China, Hubei Province, southwest Hubei mining district, Huangtoulao Mine	Longtan Fm, Main Coal Seam		1	10.7	257.0	260	255
Data from: Glasspool and Scott, 2010	Wuchiapingian, 259.8 to 254.14 mya	China, Jiangxi Province	Leping Fm, Laoshan Mbr, Seam B3		4	9.1	257.0	260	255
Data from: Glasspool and Scott, 2010	Wuchiapingian, 259.8 to 254.14 mya	China, Jiangxi Province	Leping Fm, Wangpanli Mbr, Seam C13		4	31.2	257.0	260	255
Data from: Glasspool and Scott, 2010	Wuchiapingian, 259.8 to 254.14 mya	China, Zhijin Coalfield	Seam # 16		1	19.7	257.0	260	255
Data from: Glasspool and Scott, 2010	Wuchiapingian, 259.8 to 254.14 mya	China, Zhijin Coalfield	Seam # 23		1	18.2	257.0	260	255
Data from: Glasspool and Scott, 2010	Wuchiapingian, 259.8 to 254.14 mya	China, Zhijin Coalfield	Seam # 26		1	19.9	257.0	260	255
Data from: Glasspool and Scott, 2010	Wuchiapingian, 259.8 to 254.14 mya	China, Zhijin Coalfield	Seam # 27		1	14.2	257.0	260	255
Data from: Glasspool and Scott, 2010	Wuchiapingian, 259.8 to 254.14 mya	China, Zhijin Coalfield	Seam # 28		1	19.7	257.0	260	255
Data from: Glasspool and Scott, 2010	Wuchiapingian, 259.8 to 254.14 mya	China, Zhijin Coalfield	Seam # 29		1	5.5	257.0	260	255
Data from: Glasspool and Scott, 2010	Wuchiapingian, 259.8 to 254.14 mya	China, Zhijin Coalfield	Seam # 30		1	6.4	257.0	260	255
Data from: Glasspool and Scott, 2010	Wuchiapingian, 259.8 to 254.14 mya	China, Zhijin Coalfield	Seam # 32		1	34.8	257.0	260	255
Data from: Glasspool and Scott, 2010	Wuchiapingian, 259.8 to 254.14 mya	China, Zhijin Coalfield	Seam # 34		1	26.7	257.0	260	255
Data from: Glasspool and Scott, 2010	Wuchiapingian, 259.8 to 254.14 mya	Germany, Lower Rhine Basin	Kupferschiefer	Unclear how many seams	12	8.8	257.0	260	255
Holdgate et al., 2005: Age: Slater et al., 2015	Capitanian, 265.1 to 259.8 mya	Antarctica, Lambert Graben	Bainmedart Coal Measures, Glossopteris Gully Mbr, 95/160		1	18.5	262.5	260	255
Holdgate et al., 2005: Age: Slater et al., 2015	Capitanian, 265.1 to 259.8 mya	Antarctica, Lambert Graben	Bainmedart Coal Measures, Glossopteris Gully Mbr, 95/46		1	45.9	262.5	260	255
Holdgate et al., 2005: Age: Slater et al., 2015	Capitanian, 265.1 to 259.8 mya	Antarctica, Lambert Graben	Bainmedart Coal Measures, Glossopteris Gully Mbr, 95/52		1	48.4	262.5	260	255
Holdgate et al., 2005: Age: Slater et al., 2015	Capitanian, 265.1 to 259.8 mya	Antarctica, Lambert Graben	Bainmedart Coal Measures, Glossopteris Gully Mbr, 95/55		1	51.2	262.5	260	255
Holdgate et al., 2005: Age: Slater et al., 2015	Capitanian, 265.1 to 259.8 mya	Antarctica, Lambert Graben	Bainmedart Coal Measures, Glossopteris Gully Mbr, 95/67		1	33.5	262.5	260	255
Holdgate et al., 2005: Age: Slater et al., 2015	Capitanian, 265.1 to 259.8 mya	Antarctica, Lambert Graben	Bainmedart Coal Measures, Glossopteris Gully Mbr, 95/99		1	41.1	262.5	260	255

Data from: Glasspool and Scott, 2010	Capitanian, 265.1 to 259.8 mya	Australia	Foybrook Fm, Barrett		1	24.0	262.5	260	255
Data from: Glasspool and Scott, 2010	Capitanian, 265.1 to 259.8 mya	Australia	Foybrook Fm, Upper Wynn Seam		1	59.3	262.5	260	255
Data from: Glasspool and Scott, 2010	Capitanian, 265.1 to 259.8 mya	Australia, Hunter Valley	Foybrook Fm, Lower Liddell Seam	(Table 6)	1	13.0	262.5	260	255
Data from: Glasspool and Scott, 2010	Capitanian, 265.1 to 259.8 mya	Australia, Hunter Valley	Foybrook Fm, Upper Liddell Seam	(Table 6)	1	14.7	262.5	260	255
Data from: Glasspool and Scott, 2010	Capitanian, 265.1 to 259.8 mya	Australia, Sydney Basin	Foybrook Fm, Upper Wynn		1	97.3	262.5	260	255
Data from: Glasspool and Scott, 2010 + Huleatt, 1991	Capitanian, 265.1 to 259.8 mya	Australia, Sydney Basin, Hunter Coalfield	Wittingham Coal Measures, Jerry's Plain Subgroup, Burnhamwood Fm, Mount Arthur Seam	((36.4*2)+(10.4+30.2+26.3))/5	5	27.9	262.5	260	255
Data from: Glasspool and Scott, 2010 + Huleatt, 1991	Capitanian, 265.1 to 259.8 mya	Australia, Sydney Basin, Hunter Coalfield	Wittingham Coal Measures, Jerry's Plain Subgroup, Burnhamwood Formation, Bayswater	(53.8+55.4)/2	2	54.6	262.5	260	255
Data from: Glasspool and Scott, 2010 + Huleatt, 1991	Capitanian, 265.1 to 259.8 mya	Australia, Sydney Basin, Hunter Coalfield	Wittingham Coal Measures, Jerry's Plain Subgroup, Burnhamwood Formation, Ravensworth	(36.5+42.6)/2	2	39.6	262.5	260	255
Data from: Glasspool and Scott, 2010 + Huleatt, 1991	Capitanian, 265.1 to 259.8 mya	Australia, Sydney Basin, Hunter Coalfield	Wittingham Coal Measures, Vane Subgroup, Foybrook Formation, Liddell	((15.1*4)+(11.8+15.8+15.8))/7	7	14.8	262.5	260	255
Data from: Glasspool and Scott, 2010 + Huleatt, 1991	Capitanian, 265.1 to 259.8 mya	Australia, Sydney Basin, Newcastle Coalfield	Tomago Coal Measures, Wallis Creek Formation, Rathluba		1	9.6	262.5	260	255
Data from: Glasspool and Scott, 2010 + Huleatt, 1991	Capitanian, 265.1 to 259.8 mya	Australia, Sydney Basin, Western Coalfield	Illawarra Coal Measures, Cullen Bullen Subgroup, Lithgow	((52.6*8)+(37.5+50.5+51.6))/11	11	50.9	262.5	260	255
Data from: Glasspool and Scott, 2010 + Huleatt, 1991	Capitanian, 265.1 to 259.8 mya	Australia, Sydney Basin, Western Coalfield	Illawarra Coal Measures, Ulan	(60.6 + 63.1))/2	2	61.9	262.5	260	255
Huleatt, 1991	Capitanian, 265.1 to 259.8 mya	Australia, Sydney Basin, Hunter Coalfield	Wittingham Coal Measures, Jerry's Plain Subgroup	Blakefield	1	13.3	262.5	260	255
Huleatt, 1991	Capitanian, 265.1 to 259.8 mya	Australia, Sydney Basin, Hunter Coalfield	Wittingham Coal Measures, Vane Subgroup	Pikes Gully	1	19.1	262.5	260	255
Huleatt, 1991	Capitanian, 265.1 to 259.8 mya	Australia, Sydney Basin, Newcastle Coalfield	Tomago Coal Measures	Elwells Creek	1	30.2	262.5	260	255
Huleatt, 1991	Capitanian, 265.1 to 259.8 mya	Australia, Sydney Basin, Newcastle Coalfield	Tomago Coal Measures	Whites Creek	1	27.1	262.5	260	255
Huleatt, 1991	Capitanian, 265.1 to 259.8 mya	Australia, Sydney Basin, Newcastle Coalfield	Tomago Coal Measures	Big Ben	2	20.5	262.5	260	255
Huleatt, 1991	Capitanian, 265.1 to 259.8 mya	Australia, Sydney Basin, Western Coalfield	Illawarra Coal Measures	Irondale	2	21.4	262.5	260	255
Huleatt, 1991	Guadalupian, 272.3 to 259.8 mya	Australia, Oaklands Basin, Southern NSW	Coorabin Coal Measures	Lanes Shaft	1	59.6	266.1	270	270
Data from: Glasspool and Scott, 2010	Guadalupian, 272.3 to 259.8 mya	China, SW Fujian Province	Tongziyan Fm, "Ore Layer 20"		3	7.5	266.1	270	270
Data from: Glasspool and Scott, 2010	Guadalupian, 272.3 to 259.8 mya	China, SW Fujian Province	Tongziyan Fm, "Ore Layer 39"		4	3.9	266.1	270	270

Data from: Glasspool and Scott, 2010	Guadalupian, 272.3 to 259.8 mya	China, SW Fujian Province	Tongziyan Fm, "Ore Layer 5"		9	6.0	266.1	270	270
Data from: Glasspool and Scott, 2010	Guadalupian, 272.3 to 259.8 mya	China, SW of South China			1	10.8	266.1	270	270
Data from: Glasspool and Scott, 2010	Guadalupian, 272.3 to 259.8 mya	China, SW of South China	C12		1	19.5	266.1	270	270
Data from: Glasspool and Scott, 2010	Guadalupian, 272.3 to 259.8 mya	China, SW of South China	C17		1	32.6	266.1	270	270
Data from: Glasspool and Scott, 2010	Guadalupian, 272.3 to 259.8 mya	China, SW of South China	C4		1	29.8	266.1	270	270
Data from: Glasspool and Scott, 2010	Guadalupian, 272.3 to 259.8 mya	China, SW of South China	C407		1	31.8	266.1	270	270
Data from: Glasspool and Scott, 2010	Guadalupian, 272.3 to 259.8 mya	China, SW of South China	C7		1	20.2	266.1	270	270
Data from: Glasspool and Scott, 2010	Guadalupian, 272.3 to 259.8 mya	China, SW of South China	C9		1	30.4	266.1	270	270
Data from: Glasspool and Scott, 2010	Guadalupian, 272.3 to 259.8 mya	Mozambique, Borehole A3	Seam 1		1	13.3	266.1	270	270
Data from: Glasspool and Scott, 2010	Guadalupian, 272.3 to 259.8 mya	Mozambique, Borehole A3	Seam 2		1	13.4	266.1	270	270
Data from: Glasspool and Scott, 2010	Guadalupian, 272.3 to 259.8 mya	Mozambique, Borehole C3	Seam 2		1	13.4	266.1	270	270
Data from: Glasspool and Scott, 2010	Guadalupian, 272.3 to 259.8 mya	Russia, Kemerovskaya, Aralichevskoye, Kuznets Basin	Usaytsk Fm, Seam IV		1	40.0	266.1	270	270
Data from: Glasspool and Scott, 2010	Guadalupian, 272.3 to 259.8 mya	Russia, Kemerovskaya, Baidayevsky, Kuznets Basin	Leninsk Fm, Seam 30		1	14.4	266.1	270	270
Data from: Glasspool and Scott, 2010	Guadalupian, 272.3 to 259.8 mya	Russia, Kemerovskaya, Baidayevsky, Kuznets Basin	Uskatsk Fm, Seam 14		1	7.0	266.1	270	270
Data from: Glasspool and Scott, 2010	Guadalupian, 272.3 to 259.8 mya	Russia, Kemerovskaya, Belovskoye, Kuznets Basin	Kazankovo-Markinsk Fm, Seam 5		1	13.6	266.1	270	270
Brownfield et al., 2001	Guadalupian, 272.3 to 259.8 mya	Russia, Kemerovskaya, Belovsky, Kuznetsky, Belovskoye, Pionerka 9	Permian, Guadalupian, Kol`chuginskaya Series,Erunakovskaya (P2er) Subseries, Gramoteinskaya P2gr		1	15.0	266.1	270	270
Data from: Glasspool and Scott, 2010	Guadalupian, 272.3 to 259.8 mya	Russia, Kemerovskaya, Bunguro-Chumyshsky, Kuznets Basin	Kazankovo-Markinsk Fm, Seam II		1	27.0	266.1	270	270
Data from: Glasspool and Scott, 2010	Guadalupian, 272.3 to 259.8 mya	Russia, Kemerovskaya, Chernokaltanskoye, Kuznets Basin	Kazankovo-Markinsk Fm, Seams 11-12		1	47.0	266.1	270	270
Data from: Glasspool and Scott, 2010	Guadalupian, 272.3 to 259.8 mya	Russia, Kemerovskaya, Kiseliovskoye, IV Vnutrenny District, Kuznets Basin	Usaytsk Fm		1	25.0	266.1	270	270
Brownfield et al., 2001	Guadalupian, 272.3 to 259.8 mya	Russia, Kemerovskaya, Kuznetsky, Aralichevskoye, Ordzhonikidze IV	Permian, Guadalupian, Balakhonskaya Series, Upper-Balakhonskaya (P1 bl2) Subseries, Usiatskaya P1us		1	40.0	266.1	270	270

Brownfield et al., 2001	Guadalupian, 272.3 to 259.8 mya	Russia, Kemerovskaya, Kuznetsky, Baidayevsky, Abashevskaya 14	Permian, Guadalupian, Kol`chuginskaya Series, Il`yinskaya (P2il) Subseries, Kuznetskaya P2ks		1	7.0	266.1	270	270
Brownfield et al., 2001	Guadalupian, 272.3 to 259.8 mya	Russia, Kemerovskaya, Kuznetsky, Baidayevsky, Baidayevskaya 30	Permian, Guadalupian, Kol`chuginskaya Series,Erunakovskaya (P2er) Subseries, Leninskaya P2 ln		1	14.4	266.1	270	270
Brownfield et al., 2001	Guadalupian, 272.3 to 259.8 mya	Russia, Kemerovskaya, Kuznetsky, Belovskoye, Chertinskaya 5	Permian, Guadalupian, Kol`chuginskaya Series, Il`yinskaya (P2il) Subseries, Kazankovo-Markinskaya P2km		1	13.6	266.1	270	270
Brownfield et al., 2001	Guadalupian, 272.3 to 259.8 mya	Russia, Kemerovskaya, Kuznetsky, Kiseliovskoye, Cherkasovskaya IV Vnutrenny	Permian, Guadalupian, Balakhonskaya Series, Upper-Balakhonskaya (P1 bl2) Subseries, Usiatskaya P1us		1	25.0	266.1	270	270
Brownfield et al., 2001	Guadalupian, 272.3 to 259.8 mya	Russia, Kemerovskaya, Kuznetsky, Leninskoye, 7 noyabrya Nadbaikaimsky, Baikaimsky	Permian, Guadalupian, Kol`chuginskaya Series,Erunakovskaya (P2er) Subseries, Gramoteinskaya P2gr		1	12.5	266.1	270	270
Brownfield et al., 2001	Guadalupian, 272.3 to 259.8 mya	Russia, Kemerovskaya, Kuznetsky, Leninskoye, Gramoteinsky Krasnogorsky	Permian, Guadalupian, Kol`chuginskaya Series,Erunakovskaya (P2er) Subseries, Gramoteinskaya P2gr		1	15.0	266.1	270	270
Brownfield et al., 2001	Guadalupian, 272.3 to 259.8 mya	Russia, Kemerovskaya, Kuznetsky, Leninskoye, Kirova Tolmachiovsky	Permian, Guadalupian, Kol`chuginskaya Series,Erunakovskaya (P2er) Subseries, Gramoteinskaya P2gr		1	4.0	266.1	270	270
Brownfield et al., 2001	Guadalupian, 272.3 to 259.8 mya	Russia, Kemerovskaya, Kuznetsky, Leninskoye, Polysayevskaya Nadbaikaimsky	Permian, Guadalupian, Kol`chuginskaya Series,Erunakovskaya (P2er) Subseries, Leninskaya P2 ln		1	11.0	266.1	270	270
Brownfield et al., 2001	Guadalupian, 272.3 to 259.8 mya	Russia, Kemerovskaya, Kuznetsky, Osinovsky, Vysokaya Elbansky I	Permian, Guadalupian, Kol`chuginskaya Series, Il`yinskaya (P2il) Subseries, Kazankovo-Markinskaya P2km		1	5.0	266.1	270	270
Brownfield et al., 2001	Guadalupian, 272.3 to 259.8 mya	Russia, Kemerovskaya, Kuznetsky, Tomusinskoye, Ol`zherassky IV - V	Permian, Guadalupian, Balakhonskaya Series, Upper-Balakhonskaya (P1 bl2) Subseries, Usiatskaya P1us		1	34.0	266.1	270	270
Brownfield et al., 2001	Guadalupian, 272.3 to 259.8 mya	Russia, Kemerovskaya, Kuznetsky, Tomusinskoye, Raspadskaya VI - VIa	Permian, Guadalupian, Kol`chuginskaya Series, Il`yinskaya (P2il) Subseries, Kazankovo-Markinskaya P2km		1	12.0	266.1	270	270
Brownfield et al., 2001	Guadalupian, 272.3 to 259.8 mya	Russia, Kemerovskaya, Kuznetsky,	Permian, Guadalupian, Balakhonskaya		1	43.0	266.1	270	270

		Tomusinskoye, Tomusinsky III	Series, Upper-Balakhonskaya (P1 bl2) Subseries, Usiatskaya P1us						
Brownfield et al., 2001	Guadalupian, 272.3 to 259.8 mya	Russia, Kemerovskaya, Kuznetsky, Uskatsky, Karagailinskaya Sergeyevsky	Permian, Guadalupian, Kol`chuginskaya Series, Il`yinskaya (P2il) Subseries, Kazankovo-Markinskaya P2km		1	14.0	266.1	270	270
Data from: Glasspool and Scott, 2010	Guadalupian, 272.3 to 259.8 mya	Russia, Kemerovskaya, Leninskoye, Nadbaikaimsky District, Kuznets Basin	Leninsk Fm		1	11.0	266.1	270	270
Brownfield et al., 2001	Guadalupian, 272.3 to 259.8 mya	Russia, Kemerovskaya, Leninsky, Kuznetsky, Yegozovo-Krasnoyarskoye, Yaroslavskogo Gorely	Permian, Guadalupian, Kol`chuginskaya Series,Erunakovskaya (P2er) Subseries, Gramoteinskaya P2gr		1	10.5	266.1	270	270
Data from: Glasspool and Scott, 2010	Guadalupian, 272.3 to 259.8 mya	Russia, Kemerovskaya, Osinovsky, Elbansky, Kuznets Basin	Kazankovo-Markinsk Fm, Seam I		1	5.0	266.1	270	270
Data from: Glasspool and Scott, 2010	Guadalupian, 272.3 to 259.8 mya	Russia, Kemerovskaya, Tomusinskoye, Kuznets Basin	Kazankovo-Markinsk Fm, Seams VI-VIa		1	12.0	266.1	270	270
Data from: Glasspool and Scott, 2010	Guadalupian, 272.3 to 259.8 mya	Russia, Kemerovskaya, Tomusinskoye, Kuznets Basin	Usaytsk Fm, Seam III		1	43.0	266.1	270	270
Data from: Glasspool and Scott, 2010	Guadalupian, 272.3 to 259.8 mya	Russia, Kemerovskaya, Tomusinskoye, Kuznets Basin	Usaytsk Fm, Seams IV-V		1	34.0	266.1	270	270
Data from: Glasspool and Scott, 2010	Guadalupian, 272.3 to 259.8 mya	Russia, Kemerovskaya, Various Districts, Kuznets Basin	Kazankovo-Markinsk Fm	Unclear how many seams	9	51.1	266.1	270	270
Data from: Glasspool and Scott, 2010	Guadalupian, 272.3 to 259.8 mya	Russia, Komi Republic, Pechorsky, Intinskoye	Inta Fm, Seam I1		1	14.0	266.1	270	270
Data from: Glasspool and Scott, 2010	Guadalupian, 272.3 to 259.8 mya	Russia, Kuznets Basin		Unclear how many seams	9	22.2	266.1	270	270
Holdgate et al., 2005: Age: Slater et al., 2015	Wordian, 268.8-265.1 mya	Antarctica, Lambert Graben	Bainmedart Coal Measures, Dragons Teeth Mbr, 95/133		1	34.2	267.0	270	270
Slater et al., 2015	Wordian, 268.8-265.1 mya	Antarctica, Lambert Graben	Bainmedart Coal Measures, Toploje Mbr, 95/silicified peat		1	40.8	267.0	270	270
Huleatt, 1991	Wordian, 268.8-265.1 mya	Australia, Gunnedah Basin, Gunnedah Coalfield	Black Jack Fm, Melvilles Seam		1	23.7	267.0	270	270
Holdgate et al., 2005: Age: Slater et al., 2015	Roadian, 272.3 to 268.8 mya	Antarctica, Lambert Graben	Bainmedart Coal Measures, Toploje Mbr, 95/124		1	34.2	270.6	270	270
New data from: ProNina, this paper	Roadian, 272.3 to 268.8 mya	Russia, Tungusskiy Basin, central part	Peljatkinskaya Group, Seam 1 (2.2m)		10	32.0	270.6	270	270
New data from: ProNina, this paper	Roadian, 272.3 to 268.8 mya	Russia, Tungusskiy Basin, central part	Peljatkinskaya Group, Seam 7 (1.7m)		10	13.0	270.6	270	270
New data from: ProNina, this paper	Roadian, 272.3 to 268.8 mya	Russia, Tungusskiy Basin, N-W part	Shmidtovskaya Group, Seam S3		50	41.0	270.6	270	270
New data from: ProNina, this paper	Roadian, 272.3 to 268.8 mya	Russia, Tungusskiy Basin, N-W part	Shmidtovskaya Group, Seam S4-5		50	22.0	270.6	270	270
New data from: ProNina, this paper	Roadian, 272.3 to 268.8 mya	Russia, Tungusskiy Basin, N-W part	Shmidtovskaya Group, Seam S6-7		50	29.0	270.6	270	270

New data from: ProNina, this paper	Roadian, 272.3 to 268.8 mya	Russia, Tungusskiy Basin, western part	Peljatkinskaya Group, Seam 24 (1.15m)		50	24.0	270.6	270	270
Data from: Glasspool and Scott, 2010	Kungurian, 283.5 to 272.3 mya	Australia	Lower Aldebaran Sandstone (APP3.2), Theresa and Carbine Seams		1	44.8	277.9	280	285
http://dbforms.ga.gov.au/www/npm.well.search	Kungurian, 283.5 to 272.3 mya	Australia, Northern Territories, Bonaparte Basin/Petrel Sub-basin, Well, Flat Top 1	Fm 6, Kungurian		1	49.0	277.9	280	285
http://dbforms.ga.gov.au/www/npm.well.search	Kungurian, 283.5 to 272.3 mya	Australia, Northern Territories, Bonaparte Basin/Petrel Sub-basin, Well, Flat Top 1	Fm 6, Kungurian		1	70.0	277.9	280	285
Smyth, 1972	Kungurian, 283.5 to 272.3 mya	Australia, South Australia, Mudrangie No. 1 Well, Cooper Basin, Patchawarra Trough	Epsilon Fm	Table 4.3: 2751.5-2764.5m	1	66.0	277.9	280	285
Smyth, 1972	Kungurian, 283.5 to 272.3 mya	Australia, South Australia, Mudrangie No. 1 Well, Cooper Basin, Patchawarra Trough	Epsilon Fm	Table 4.6: 2761.5-2767.6m	1	60.0	277.9	280	285
Smyth, 1972	Kungurian, 283.5 to 272.3 mya	Australia, South Australia, Mudrangie No. 1 Well, Cooper Basin, Patchawarra Trough	Epsilon Fm	Table 4.3: 2764.5-2767.6m	1	54.0	277.9	280	285
Smyth, 1972	Kungurian, 283.5 to 272.3 mya	Australia, South Australia, Mudrangie No. 1 Well, Cooper Basin, Patchawarra Trough	Epsilon Fm	Table 4.6: 2761.5-2767.6m: Epsilon 1 Coal Seam	1	60.0	277.9	280	285
Smyth, 1972	Kungurian, 283.5 to 272.3 mya	Australia, South Australia, Tindilpie No. 1 Well, Cooper Basin, Patchawarra Trough	Epsilon Fm	Table 4.13: 2660.9-2664.0m	1	49.0	277.9	280	285
Smyth, 1972	Kungurian, 283.5 to 272.3 mya	Australia, South Australia, Tindilpie No. 1 Well, Cooper Basin, Patchawarra Trough	Epsilon Fm	Table 4.13: 2667.0-2670.0m	1	55.0	277.9	280	285
Smyth, 1972	Kungurian, 283.5 to 272.3 mya	Australia, South Australia, Tindilpie No. 1 Well, Cooper Basin, Patchawarra Trough	Epsilon Fm	Table 4.13: 2670.0-2673.1m	1	46.0	277.9	280	285
Smyth, 1972	Kungurian, 283.5 to 272.3 mya	Australia, South Australia, Tindilpie No. 1 Well, Cooper Basin, Patchawarra Trough	Epsilon Fm	Table 4.13: 2679.2-2682.2m	1	56.0	277.9	280	285
Smyth, 1972	Kungurian, 283.5 to 272.3 mya	Australia, South Australia, Tindilpie No. 1 Well, Cooper Basin, Patchawarra Trough	Epsilon Fm	Table 4.13: 2682.2-2685.3m	1	64.0	277.9	280	285
Smyth, 1972	Kungurian, 283.5 to 272.3 mya	Australia, South Australia, Tindilpie No. 1 Well, Cooper Basin, Patchawarra Trough	Epsilon Fm	Table 4.13: 2685.3-2688.3m	1	56.0	277.9	280	285
Smyth, 1972	Kungurian, 283.5 to 272.3 mya	Australia, South Australia, Tindilpie No. 1 Well, Cooper Basin, Patchawarra Trough	Epsilon Fm	Table 4.13: 2688.3-2691.4m	1	59.0	277.9	280	285
Smyth, 1972	Kungurian, 283.5 to 272.3 mya	Australia, South Australia, Tindilpie No. 1 Well, Cooper	Epsilon Fm	Table 4.13: 2694.4-2697.5m	1	43.0	277.9	280	285

		Basin, Patchawarra Trough							
Smyth, 1972	Kungurian, 283.5 to 272.3 mya	Australia, South Australia, Tindilpie No. 1 Well, Cooper Basin, Patchawarra Trough	Epsilon Fm	Table 4.13: 2697.5-2700.5m	1	54.0	277.9	280	285
Smyth, 1972	Kungurian, 283.5 to 272.3 mya	Australia, South Australia, Tindilpie No. 1 Well, Cooper Basin, Patchawarra Trough	Epsilon Fm	Table 4.13: 2700.5-2703.6m	1	42.0	277.9	280	285
Smyth, 1972	Kungurian, 283.5 to 272.3 mya	Australia, South Australia, Tindilpie No. 1 Well, Cooper Basin, Patchawarra Trough	Epsilon Fm	Table 4.13: 2703.6-2706.6m	1	44.0	277.9	280	285
Smyth, 1972	Kungurian, 283.5 to 272.3 mya	Australia, South Australia, Tindilpie No. 1 Well, Cooper Basin, Patchawarra Trough	Epsilon Fm	Table 4.13: 2706.6-2709.7m	1	46.0	277.9	280	285
Smyth, 1972	Kungurian, 283.5 to 272.3 mya	Australia, South Australia, Tindilpie No. 1 Well, Cooper Basin, Patchawarra Trough	Epsilon Fm	Table 4.13: 2712.7-2715.8m	1	52.0	277.9	280	285
Smyth, 1972	Kungurian, 283.5 to 272.3 mya	Australia, South Australia, Tindilpie No. 1 Well, Cooper Basin, Patchawarra Trough	Roseneath Shale	Table 4.12: 8620-8630ft	1	59.0	277.9	280	285
Smyth, 1972	Kungurian, 283.5 to 272.3 mya	Australia, South Australia, Tindilpie No. 1 Well, Cooper Basin, Patchawarra Trough	Roseneath Shale	Table 4.12: 8630-8640ft	1	54.0	277.9	280	285
Smyth, 1972	Kungurian, 283.5 to 272.3 mya	Australia, South Australia, Tindilpie No. 1 Well, Cooper Basin, Patchawarra Trough	Roseneath Shale	Table 4.12: 8650-8660ft	1	43.0	277.9	280	285
Smyth, 1972	Kungurian, 283.5 to 272.3 mya	Australia, South Australia, Tindilpie No. 1 Well, Cooper Basin, Patchawarra Trough	Roseneath Shale	Table 4.12: 8660-8670ft	1	39.0	277.9	280	285
Smyth, 1972	Kungurian, 283.5 to 272.3 mya	Australia, South Australia, Tindilpie No. 1 Well, Cooper Basin, Patchawarra Trough	Roseneath Shale	Table 4.12: 8670-8680ft	1	40.0	277.9	280	285
Smyth, 1972	Kungurian, 283.5 to 272.3 mya	Australia, South Australia, Tindilpie No. 1 Well, Cooper Basin, Patchawarra Trough	Roseneath Shale	Table 4.12: 8690-8700ft	1	50.0	277.9	280	285
Smyth, 1972	Kungurian, 283.5 to 272.3 mya	Australia, South Australia, Tindilpie No. 1 Well, Cooper Basin, Patchawarra Trough	Roseneath Shale	Table 4.12: 8700-8710ft	1	47.0	277.9	280	285
Data from: Glasspool and Scott, 2010	Kungurian, 283.5 to 272.3 mya	Russia, Kemerovskaya, Dvoinoy and Podsporny Districts	Ishanovsk Fm	Unclear how many seams	2	54.5	277.9	280	285
Brownfield et al., 2001	Kungurian, 283.5 to 272.3 mya	Russia, Kemerovskaya, Gorlovsky, Listvianskaya Dvoinoy	Permian, Kungurian, Balakhonskaya Series, Upper-Balakhonskaya (P1 bl2) Subseries, Ishanovskaya P1i		1	68.0	277.9	280	285
Brownfield et al., 2001	Kungurian, 283.5 to 272.3 mya	Russia, Kemerovskaya, Kuznetsky, Prokop`yevskoye,	Permian, Kungurian, Balakhonskaya Series, Upper-		1	41.0	277.9	280	285

		Zenkovskaya Podsporny	Balakhonskaya (P1 bl2) Subseries, Ishanovskaya P1i						
Data from: Glasspool and Scott, 2010	Kungurian, 283.5 to 272.3 mya	Russia, Komi Republic, Pechorsky, Khal`mer-Yuskoy	Lek-Vorkuta Fm, Seam K5		1	20.1	277.9	280	285
Data from: Glasspool and Scott, 2010	Kungurian, 283.5 to 272.3 mya	Russia, Komi Republic, Pechorsky, Moschny Troinoy Districts	Lek-Vorkuta Fm	Unclear how many seams	2	21.0	277.9	280	285
Data from: Glasspool and Scott, 2010	Kungurian, 283.5 to 272.3 mya	Russia, Komi Republic, Pechorsky, Vorkutskoye	Lek-Vorkuta Fm, Seam I4		1	22.0	277.9	280	285
Data from: Glasspool and Scott, 2010	Kungurian, 283.5 to 272.3 mya	Russia, Komi Republic, Pechorsky, Yun`-Yaginskoye	Lek-Vorkuta Fm, Seam n14		1	20.0	277.9	280	285
Data from: Glasspool and Scott, 2010 + Huleatt, 1991	Artinskian to Kungurian, 290.1 to 272.3 mya	Australia, Bowen Basin	Ashford Coal Measures		1	44.0	281.2	280	285
Data from: Glasspool and Scott, 2010 + Huleatt, 1991	Artinskian to Kungurian, 290.1 to 272.3 mya	Australia, Bowen Basin	Collinsville Coal Measures (lower), Blake	((58.6)+(12.8+20.7))/3	3	30.7	281.2	280	285
Data from: Glasspool and Scott, 2010 + Huleatt, 1991	Artinskian to Kungurian, 290.1 to 272.3 mya	Australia, Bowen Basin	Collinsville Coal Measures (lower), Bowen	(54.8+59.4)/2	2	57.1	281.2	280	285
Huleatt, 1991	Artinskian to Kungurian, 290.1 to 272.3 mya	Australia, Bowen Basin	Collinsville Coal Measures, Denison		1	25.0	281.2	280	285
Huleatt, 1991	Artinskian to Kungurian, 290.1 to 272.3 mya	Australia, Bowen Basin	Collinsville Coal Measures, Garrick		2	60.4	281.2	280	285
Data from: Glasspool and Scott, 2010	Artinskian to Kungurian, 290.1 to 272.3 mya	Australia, Gunnedah Basin, Gunnedah Coalfield	Maules Creek Fm, Coal at Depth 944.40m		1	17.9	281.2	280	285
Data from: Glasspool and Scott, 2010	Artinskian to Kungurian, 290.1 to 272.3 mya	Australia, Gunnedah Basin, Gunnedah Coalfield	Maules Creek Fm, Coal at Depth 971.00m		1	35.0	281.2	280	285
Huleatt, 1991	Artinskian to Kungurian, 290.1 to 272.3 mya	Australia, Gunnedah Basin, Gunnedah Coalfield	Maules Creek Fm, Gundawarra		1	42.9	281.2	280	285
Data from: Glasspool and Scott, 2010 + Huleatt, 1991	Artinskian to Kungurian, 290.1 to 272.3 mya	Australia, Queensland, Blair Athol Basin, Clermont	Blair Athol #3	((67*1)+(60*2))/3	3	62.3	281.2	280	285
Smyth, 1972	Artinskian to Kungurian, 290.1 to 272.3 mya	Australia, South Australia, Mudrangie No. 1 Well, Cooper Basin, Patchawarra Trough	Murteree Fm	Table 4.3: 2798.1-2801.1m	1	66.0	281.2	280	285
Smyth, 1972	Artinskian to Kungurian, 290.1 to 272.3 mya	Australia, South Australia, Tindilpie No. 1 Well, Cooper Basin, Patchawarra Trough	Murteree Fm	Table 4.14: 8910-8920ft	1	51.0	281.2	280	285
Smyth, 1972	Artinskian to Kungurian, 290.1 to 272.3 mya	Australia, South Australia, Tindilpie No. 1 Well, Cooper Basin, Patchawarra Trough	Murteree Fm	Table 4.14: 8940-8950ft	1	29.0	281.2	280	285
Smyth, 1972	Artinskian to Kungurian, 290.1 to 272.3 mya	Australia, South Australia, Tindilpie No. 1 Well, Cooper Basin, Patchawarra Trough	Murteree Fm	Table 4.14: 8960-8970ft	1	33.0	281.2	280	285
Smyth, 1972	Artinskian to Kungurian, 290.1 to 272.3 mya	Australia, South Australia, Tindilpie No. 1 Well, Cooper Basin, Patchawarra Trough	Murteree Fm	Table 4.14: 8980-8990ft	1	50.0	281.2	280	285
Smyth, 1972	Artinskian to Kungurian, 290.1 to 272.3 mya	Australia, South Australia, Tindilpie No. 1 Well, Cooper Basin, Patchawarra Trough	Murteree Fm	Table 4.14: 9000-9010ft	1	30.0	281.2	280	285

Data from: Glasspool and Scott, 2010	Artinskian to Kungurian, 290.1 to 272.3 mya	China, W. Hunan Province	Liangshan Fm	Unclear how many seams	2	10.0	281.2	280	285
Data from: Glasspool and Scott, 2010	Artinskian to Kungurian, 290.1 to 272.3 mya	India, East Bokaro Coalfield	Barakar Fm, Jarangdi Bottom Seam		2	39.8	281.2	280	285
Data from: Glasspool and Scott, 2010	Artinskian to Kungurian, 290.1 to 272.3 mya	India, East Bokaro Coalfield	Barakar Fm, Jarangdi Seam		8	29.0	281.2	280	285
Data from: Glasspool and Scott, 2010	Artinskian to Kungurian, 290.1 to 272.3 mya	India, East Bokaro Coalfield	Barakar Fm, Jarangdi Top Seam		3	39.3	281.2	280	285
Data from: Glasspool and Scott, 2010	Artinskian to Kungurian, 290.1 to 272.3 mya	India, East Bokaro Coalfield	Barakar Fm, Kargali Bottom Seam		6	45.1	281.2	280	285
Data from: Glasspool and Scott, 2010	Artinskian to Kungurian, 290.1 to 272.3 mya	India, East Bokaro Coalfield	Barakar Fm, Kargali Seam		16	27.7	281.2	280	285
Data from: Glasspool and Scott, 2010	Artinskian to Kungurian, 290.1 to 272.3 mya	India, East Bokaro Coalfield	Barakar Fm, Kargali Top Seam		12	31.9	281.2	280	285
Data from: Glasspool and Scott, 2010	Artinskian to Kungurian, 290.1 to 272.3 mya	India, East Bokaro Coalfield	Barakar Fm, Kathara Seam		8	31.0	281.2	280	285
Data from: Glasspool and Scott, 2010	Artinskian to Kungurian, 290.1 to 272.3 mya	India, East Bokaro Coalfield	Barakar Fm, Uchitdih Seam		9	20.7	281.2	280	285
Data from: Glasspool and Scott, 2010	Artinskian to Kungurian, 290.1 to 272.3 mya	India, Jharia Coalfield	Karharbari Fm, Seam I		1	37.9	281.2	280	285
Data from: Glasspool and Scott, 2010	Artinskian to Kungurian, 290.1 to 272.3 mya	India, Jharia Coalfield	Middle Barakar Fm, Seam XI-XVI		1	53.9	281.2	280	285
Data from: Glasspool and Scott, 2010	Artinskian to Kungurian, 290.1 to 272.3 mya	India, Jharia Coalfield	Upper Barakar Fm, Seam XVII-XVIII		1	44.4	281.2	280	285
Data from: Glasspool and Scott, 2010	Artinskian to Kungurian, 290.1 to 272.3 mya	India, Jharkand, Sawang Colliery	Bermo		1	31.0	281.2	280	285
Data from: Glasspool and Scott, 2010	Artinskian to Kungurian, 290.1 to 272.3 mya	India, Jharkand, Sawang Colliery	Jarangdih 6 feet		1	33.9	281.2	280	285
Data from: Glasspool and Scott, 2010	Artinskian to Kungurian, 290.1 to 272.3 mya	India, Jharkand, Sawang Colliery	Jarangdih new		1	35.3	281.2	280	285
Data from: Glasspool and Scott, 2010	Artinskian to Kungurian, 290.1 to 272.3 mya	India, Jharkand, Sawang Colliery	Jarangdih-IV		1	18.1	281.2	280	285
Data from: Glasspool and Scott, 2010	Artinskian to Kungurian, 290.1 to 272.3 mya	India, Jharkand, Sawang Colliery	Karo-VIII		1	43.1	281.2	280	285
Data from: Glasspool and Scott, 2010	Artinskian to Kungurian, 290.1 to 272.3 mya	India, Palamau, Bihar, Auranga Coalfield	Upper Barakar Fm, Bagdagga Nala Seam		2	61.7	281.2	280	285
Data from: Glasspool and Scott, 2010	Artinskian to Kungurian, 290.1 to 272.3 mya	India, Palamau, Bihar, Auranga Coalfield	Upper Barakar Fm, Jagaldagga Seam		4	61.1	281.2	280	285
Data from: Glasspool and Scott, 2010	Artinskian to Kungurian, 290.1 to 272.3 mya	India, Palamau, Bihar, Auranga Coalfield	Upper Barakar Fm, Seam II		3	50.8	281.2	280	285
Data from: Glasspool and Scott, 2010	Artinskian to Kungurian, 290.1 to 272.3 mya	India, Palamau, Bihar, Auranga Coalfield	Upper Barakar Fm, Seam III		4	55.6	281.2	280	285
Data from: Glasspool and Scott, 2010	Artinskian to Kungurian, 290.1 to 272.3 mya	India, Palamau, Bihar, Auranga Coalfield	Upper Barakar Fm, Seam IV		2	63.8	281.2	280	285
Data from: Glasspool and Scott, 2010	Artinskian to Kungurian, 290.1 to 272.3 mya	India, Satpura Basin, Pench Valley, Kanhan Valley and Tawa Valley Coalfields & Sohagpur Coalfield	Barakar Fm	Seams indivisible or unclear how many seams	137	39.6	281.2	280	285
Data from: Glasspool and Scott, 2010	Artinskian to Kungurian, 290.1 to 272.3 mya	India, Singrauli Coalfield	Purewa Bottom seam		11	31.9	281.2	280	285

Data from: Glasspool and Scott, 2010	Artinskian to Kungurian, 290.1 to 272.3 mya	India, Singrauli Coalfield	Purewa Merged seam		12	25.3	281.2	280	285
Data from: Glasspool and Scott, 2010	Artinskian to Kungurian, 290.1 to 272.3 mya	India, Singrauli Coalfield	Purewa Top seam		9	41.8	281.2	280	285
Data from: Glasspool and Scott, 2010	Artinskian to Kungurian, 290.1 to 272.3 mya	India, Singrauli Coalfield	Turra seam		22	36.4	281.2	280	285
Data from: Glasspool and Scott, 2010	Artinskian to Kungurian, 290.1 to 272.3 mya	India, West Bokaro Coalfield	Barakar Fm, Seam I		2	65.5	281.2	280	285
Data from: Glasspool and Scott, 2010	Artinskian to Kungurian, 290.1 to 272.3 mya	India, West Bokaro Coalfield	Barakar Fm, Seam II		1	69.0	281.2	280	285
Data from: Glasspool and Scott, 2010	Artinskian to Kungurian, 290.1 to 272.3 mya	India, West Bokaro Coalfield	Barakar Fm, Seam II bottom		1	82.0	281.2	280	285
Data from: Glasspool and Scott, 2010	Artinskian to Kungurian, 290.1 to 272.3 mya	India, West Bokaro Coalfield	Barakar Fm, Seam II upper		1	56.0	281.2	280	285
Data from: Glasspool and Scott, 2010	Artinskian to Kungurian, 290.1 to 272.3 mya	India, West Bokaro Coalfield	Barakar Fm, Seam III		2	38.0	281.2	280	285
Data from: Glasspool and Scott, 2010	Artinskian to Kungurian, 290.1 to 272.3 mya	India, West Bokaro Coalfield	Barakar Fm, Seam IV		3	30.0	281.2	280	285
Data from: Glasspool and Scott, 2010	Artinskian to Kungurian, 290.1 to 272.3 mya	India, West Bokaro Coalfield	Barakar Fm, Seam V		2	15.5	281.2	280	285
Data from: Glasspool and Scott, 2010	Artinskian to Kungurian, 290.1 to 272.3 mya	India, West Bokaro Coalfield	Barakar Fm, Seam VA		2	51.5	281.2	280	285
Data from: Glasspool and Scott, 2010	Artinskian to Kungurian, 290.1 to 272.3 mya	India, West Bokaro Coalfield	Barakar Fm, Seam VI		1	57.0	281.2	280	285
Data from: Glasspool and Scott, 2010	Artinskian to Kungurian, 290.1 to 272.3 mya	India, West Bokaro Coalfield	Barakar Fm, Seam VI top		1	44.0	281.2	280	285
Data from: Glasspool and Scott, 2010	Artinskian to Kungurian, 290.1 to 272.3 mya	India, West Bokaro Coalfield	Barakar Fm, Seam VII		2	36.0	281.2	280	285
Data from: Glasspool and Scott, 2010	Artinskian to Kungurian, 290.1 to 272.3 mya	India, West Bokaro Coalfield	Barakar Fm, Seam VIIA		1	43.0	281.2	280	285
Data from: Glasspool and Scott, 2010	Artinskian to Kungurian, 290.1 to 272.3 mya	India, West Bokaro Coalfield	Barakar Fm, Seam VIIB		1	57.0	281.2	280	285
Data from: Glasspool and Scott, 2010	Artinskian to Kungurian, 290.1 to 272.3 mya	India, West Bokaro Coalfield	Barakar Fm, Seam VIII		1	30.0	281.2	280	285
Data from: Glasspool and Scott, 2010	Artinskian to Kungurian, 290.1 to 272.3 mya	Mozambique, Borehole A3	Seam 3		13	16.3	281.2	280	285
Data from: Glasspool and Scott, 2010	Artinskian to Kungurian, 290.1 to 272.3 mya	Mozambique, Borehole A3	Seam 3		2	18.5	281.2	280	285
Data from: Glasspool and Scott, 2010	Artinskian to Kungurian, 290.1 to 272.3 mya	Mozambique, Borehole C3	Seam 4		2	14.4	281.2	280	285
Data from: Glasspool and Scott, 2010	Artinskian to Kungurian, 290.1 to 272.3 mya	South Africa, Highveld Coalfield		Unclear how many seams	9	71.5	281.2	280	285
Data from: Glasspool and Scott, 2010	Artinskian to Kungurian, 290.1 to 272.3 mya	South Africa, Karoo Basin	Undifferentiated coals of Ecca age	(multiple mines, each mine extracts multiple seams)	53	56.8	281.2	280	285
Data from: Glasspool and Scott, 2010	Artinskian to Kungurian, 290.1 to 272.3 mya	South Africa, Karro Basin	Witbank No. 2 Seam		11	66.2	281.2	280	285
Data from: Glasspool and Scott, 2010	Artinskian to Kungurian, 290.1 to 272.3 mya	South Africa, Karro Basin	Witbank No. 4 Seam		9	56.7	281.2	280	285
Data from: Glasspool and Scott, 2010	Artinskian to Kungurian, 290.1 to 272.3 mya	Tanzania, Galula Coalfield		Unclear how many seams	2	81.0	281.2	280	285

Data from: Glasspool and Scott, 2010	Artinskian to Kungurian, 290.1 to 272.3 mya	Tanzania, Kiwira Mine	Seam 3B		1	33.0	281.2	280	285
Data from: Glasspool and Scott, 2010	Artinskian to Kungurian, 290.1 to 272.3 mya	Tanzania, Kiwira Mine	Seam 3B		1	51.0	281.2	280	285
Data from: Glasspool and Scott, 2010	Artinskian to Kungurian, 290.1 to 272.3 mya	Tanzania, Kiwira Mine	Seam 5		1	77.0	281.2	280	285
Data from: Glasspool and Scott, 2010	Artinskian to Kungurian, 290.1 to 272.3 mya	Tanzania, Kiwira Mine	Seam 6		1	83.0	281.2	280	285
Data from: Glasspool and Scott, 2010	Artinskian to Kungurian, 290.1 to 272.3 mya	Tanzania, Lema River	Seam 1		1	51.0	281.2	280	285
Data from: Glasspool and Scott, 2010	Artinskian to Kungurian, 290.1 to 272.3 mya	Tanzania, Lema River	Seam 2u		1	41.0	281.2	280	285
Data from: Glasspool and Scott, 2010	Artinskian to Kungurian, 290.1 to 272.3 mya	Tanzania, Llima Mine	Seam 3A		1	27.0	281.2	280	285
Data from: Glasspool and Scott, 2010	Artinskian to Kungurian, 290.1 to 272.3 mya	Tanzania, Llima Mine	Seam 3B		1	29.0	281.2	280	285
Data from: Glasspool and Scott, 2010	Artinskian to Kungurian, 290.1 to 272.3 mya	Tanzania, Muze Coalfield		Unclear how many seams	10	53.9	281.2	280	285
Data from: Glasspool and Scott, 2010	Artinskian to Kungurian, 290.1 to 272.3 mya	Tanzania, Mwalesi River	Seam 2l		1	26.0	281.2	280	285
Data from: Glasspool and Scott, 2010	Artinskian to Kungurian, 290.1 to 272.3 mya	Tanzania, Mwalesi River	Seam 2u		1	42.0	281.2	280	285
Data from: Glasspool and Scott, 2010	Artinskian to Kungurian, 290.1 to 272.3 mya	Tanzania, Mwalesi River	Seam 3A		2	48.0	281.2	280	285
Data from: Glasspool and Scott, 2010	Artinskian to Kungurian, 290.1 to 272.3 mya	Tanzania, Mwalesi River	Seam 6		1	58.0	281.2	280	285
Data from: Glasspool and Scott, 2010	Artinskian to Kungurian, 290.1 to 272.3 mya	Tanzania, Mwalesi River	Seam 9		1	83.0	281.2	280	285
Data from: Glasspool and Scott, 2010	Artinskian to Kungurian, 290.1 to 272.3 mya	Tanzania, Namwele-Mkomolo Coalfield		Unclear how many seams	4	36.8	281.2	280	285
Tewalt et al., 2010	Artinskian to Kungurian, 290.1 to 272.3 mya	Zambia, Maamba Coal Basin/Coal Field, Izuma Mine	Permian, Artinskian-Kungurian, Sample # Izuma Mudala Pit 1-0.0m + Zambia2-8		1	95.9	281.2	280	285
Tewalt et al., 2010	Artinskian to Kungurian, 290.1 to 272.3 mya	Zambia, Maamba Coal Basin/Coal Field, Izuma Mine	Permian, Artinskian-Kungurian, Sample # Izuma Mudala Pit 2-1.0m + Zambia2-8		1	97.7	281.2	280	285
Tewalt et al., 2010	Artinskian to Kungurian, 290.1 to 272.3 mya	Zambia, Maamba Coal Basin/Coal Field, Izuma Mine	Permian, Artinskian-Kungurian, Sample # Izuma Mudala Pit 3-2.0m + Zambia2-8		1	100.0	281.2	280	285
Tewalt et al., 2010	Artinskian to Kungurian, 290.1 to 272.3 mya	Zambia, Maamba Coal Basin/Coal Field, Izuma Mine	Permian, Artinskian-Kungurian, Sample # Izuma Mudala Pit 4-3.2m + Zambia2-8		1	99.8	281.2	280	285
Tewalt et al., 2010	Artinskian to Kungurian, 290.1 to 272.3 mya	Zambia, Maamba Coal Basin/Coal Field, Izuma Mine	Permian, Artinskian-Kungurian, Sample # Izuma Mudala Pit 5-1.5m + Zambia2-8		1	88.2	281.2	280	285

Tewalt et al., 2010	Artinskian to Kungurian, 290.1 to 272.3 mya	Zambia, Maamba Coal Basin/Coal Field, Izuma Mine	Permian, Artinskian-Kungurian, Sample # Izuma Mudala Pit 6-4.0m + Zambia2-8		1	100.0	281.2	280	285
Tewalt et al., 2010	Artinskian to Kungurian, 290.1 to 272.3 mya	Zambia, Maamba Coal Basin/Coal Field, Izuma Mine	Permian, Artinskian-Kungurian, Sample # Izuma Pit 1- 0.0m + Zambia2-7		1	92.4	281.2	280	285
Tewalt et al., 2010	Artinskian to Kungurian, 290.1 to 272.3 mya	Zambia, Maamba Coal Basin/Coal Field, Izuma Mine	Permian, Artinskian-Kungurian, Sample # Izuma Pit 2- 0.5m + Zambia2-7		1	89.6	281.2	280	285
Tewalt et al., 2010	Artinskian to Kungurian, 290.1 to 272.3 mya	Zambia, Maamba Coal Basin/Coal Field, Izuma Mine	Permian, Artinskian-Kungurian, Sample # Izuma Pit 3-1.0m + Zambia2-7		1	99.4	281.2	280	285
Tewalt et al., 2010	Artinskian to Kungurian, 290.1 to 272.3 mya	Zambia, Maamba Coal Basin/Coal Field, Izuma Mine	Permian, Artinskian-Kungurian, Sample # Izuma Pit 4-1.5m + Zambia2-7		1	97.3	281.2	280	285
Tewalt et al., 2010	Artinskian to Kungurian, 290.1 to 272.3 mya	Zambia, Maamba Coal Basin/Coal Field, Izuma Mine	Permian, Artinskian-Kungurian, Sample # Izuma Pit 5-2.0m + Zambia2-7		1	86.6	281.2	280	285
Tewalt et al., 2010	Artinskian to Kungurian, 290.1 to 272.3 mya	Zambia, Maamba Coal Basin/Coal Field, Izuma Mine	Permian, Artinskian-Kungurian, Sample # Izuma Pit 6-2.5m + Zambia2-7		1	87.6	281.2	280	285
Tewalt et al., 2010	Artinskian to Kungurian, 290.1 to 272.3 mya	Zambia, Maamba Coal Basin/Coal Field, Izuma Mine	Permian, Artinskian-Kungurian, Sample # Izuma Pit 7-3.0m + Zambia2-7		1	93.2	281.2	280	285
Data from: Glasspool and Scott, 2010	Artinskian, 290.1 to 283.5 mya	Australia	Greta Coal Measures		7	20.3	286.8	290	285
Data from: Glasspool and Scott, 2010	Artinskian, 290.1 to 283.5 mya	Australia	Greta Coal Measures	Unclear how many seams	2	25.2	286.8	290	285
Data from: Glasspool and Scott, 2010	Artinskian, 290.1 to 283.5 mya	Australia	Irwin River Coal Measures, Seam G		1	42.4	286.8	290	285
Data from: Glasspool and Scott, 2010	Artinskian, 290.1 to 283.5 mya	Australia, Collie Basin, Premier Subbasin	Seam E10		3	53.5	286.8	290	285
Data from: Glasspool and Scott, 2010	Artinskian, 290.1 to 283.5 mya	Australia, Collie Basin, Premier Subbasin	Seam E15		1	63.4	286.8	290	285
Data from: Glasspool and Scott, 2010	Artinskian, 290.1 to 283.5 mya	Australia, Collie Basin, Premier Subbasin	Seam E20		3	54.8	286.8	290	285
Data from: Glasspool and Scott, 2010	Artinskian, 290.1 to 283.5 mya	Australia, Collie Basin, Premier Subbasin	Seam E22		2	59.0	286.8	290	285
Data from: Glasspool and Scott, 2010	Artinskian, 290.1 to 283.5 mya	Australia, Collie Basin, Premier Subbasin	Seam E25		2	64.0	286.8	290	285
Data from: Glasspool and Scott, 2010	Artinskian, 290.1 to 283.5 mya	Australia, Collie Basin, Premier Subbasin	Seam E30		4	50.8	286.8	290	285
Data from: Glasspool and Scott, 2010	Artinskian, 290.1 to 283.5 mya	Australia, Collie Basin, Premier Subbasin	Seam E35		1	67.5	286.8	290	285
Data from: Glasspool and Scott, 2010	Artinskian, 290.1 to 283.5 mya	Australia, Collie Basin, Premier Subbasin	Seam E40		1	46.8	286.8	290	285
Data from: Glasspool and Scott, 2010	Artinskian, 290.1 to 283.5 mya	Australia, Collie Basin, Premier Subbasin	Seam E50		2	48.7	286.8	290	285

Data from: Glasspool and Scott, 2010	Artinskian, 290.1 to 283.5 mya	Australia, Collie Basin, Premier Subbasin	Seam E60		2	46.4	286.8	290	285
Data from: Glasspool and Scott, 2010	Artinskian, 290.1 to 283.5 mya	Australia, Collie Basin, Premier Subbasin	Seam E70		1	43.5	286.8	290	285
Data from: Glasspool and Scott, 2010	Artinskian, 290.1 to 283.5 mya	Australia, Collie Basin, Premier Subbasin	Seam E80		2	48.0	286.8	290	285
Data from: Glasspool and Scott, 2010	Artinskian, 290.1 to 283.5 mya	Australia, Collie Basin, Premier Subbasin	Seam E9		2	48.8	286.8	290	285
Data from: Glasspool and Scott, 2010	Artinskian, 290.1 to 283.5 mya	Australia, Collie Basin, Premier Subbasin	Seam E90		1	47.0	286.8	290	285
Data from: Glasspool and Scott, 2010	Artinskian, 290.1 to 283.5 mya	Australia, Irwin River Basin	H Seam		1	65.7	286.8	290	285
Huleatt, 1991	Artinskian, 290.1 to 283.5 mya	Australia, Sydney Basin, Hunter Coalfield	Greta Coal Measures	Puxtrees	1	20.2	286.8	290	285
Data from: Glasspool and Scott, 2010 + Huleatt, 1991	Artinskian, 290.1 to 283.5 mya	Australia, Sydney Basin, Hunter Coalfield	Greta Coal Measures, Skeletar Formation, Balmoral		3	35.7	286.8	290	285
Data from: Glasspool and Scott, 2010 + Huleatt, 1991	Artinskian, 290.1 to 283.5 mya	Australia, Sydney Basin, Hunter Coalfield	Greta Coal Measures, Skeletar Formation, Lewis		2	43.3	286.8	290	285
Huleatt, 1991	Artinskian, 290.1 to 283.5 mya	Australia, Sydney Basin, Newcastle Coalfield	Greta Coal Measures	Greta	3	20.0	286.8	290	285
Data from: Glasspool and Scott, 2010 + Huleatt, 1991	Artinskian, 290.1 to 283.5 mya	Australia, Sydney Basin, Newcastle Coalfield	Greta Coal Measures, Homeville		1	25.8	286.8	290	285
Data from: Glasspool and Scott, 2010 + Huleatt, 1991	Artinskian, 290.1 to 283.5 mya	Australia, Sydney Basin, Cranky Corner Basin, Newcastle Coalfield	Greta Coal Measures, Tangorin		1	26.0	286.8	290	285
Data from: Glasspool and Scott, 2010	Artinskian, 290.1 to 283.5 mya	Australia, Tasmania	Catos Creek Lower		1	31.3	286.8	290	285
Data from: Glasspool and Scott, 2010	Artinskian, 290.1 to 283.5 mya	Australia, Tasmania	Catos Creek Upper		1	32.7	286.8	290	285
Data from: Glasspool and Scott, 2010	Artinskian, 290.1 to 283.5 mya	Australia, Tasmania	Hunstmans Creek		1	40.9	286.8	290	285
Data from: Glasspool and Scott, 2010	Artinskian, 290.1 to 283.5 mya	Australia, Vasse Shelf	Sue Coal Measures, Seam A		1	48.7	286.8	290	285
Data from: Glasspool and Scott, 2010	Artinskian, 290.1 to 283.5 mya	Australia, Vasse Shelf	Sue Coal Measures, Seam B		1	48.7	286.8	290	285
Data from: Glasspool and Scott, 2010	Artinskian, 290.1 to 283.5 mya	Australia, Vasse Shelf	Sue Coal Measures, Seam C		1	54.4	286.8	290	285
Data from: Glasspool and Scott, 2010	Artinskian, 290.1 to 283.5 mya	Australia, Vasse Shelf	Sue Coal Measures, Seam D		1	43.0	286.8	290	285
Data from: Glasspool and Scott, 2010	Artinskian, 290.1 to 283.5 mya	Australia, Vasse Shelf	Sue Coal Measures, Seam E		1	54.2	286.8	290	285
Data from: Glasspool and Scott, 2010	Artinskian, 290.1 to 283.5 mya	Australia, Vasse Shelf	Sue Coal Measures, Seam F		1	46.7	286.8	290	285
Data from: Glasspool and Scott, 2010	Artinskian, 290.1 to 283.5 mya	Australia, Vasse Shelf	Sue Coal Measures, Seam G		1	49.4	286.8	290	285
Data from: Glasspool and Scott, 2010	Artinskian, 290.1 to 283.5 mya	Australia, Vasse Shelf	Sue Coal Measures, Seam H		1	51.2	286.8	290	285
Data from: Glasspool and Scott, 2010	Artinskian, 290.1 to 283.5 mya	Australia, Vasse Shelf	Sue Coal Measures, Seam I		1	46.7	286.8	290	285

Data from: Glasspool and Scott, 2010	Artinskian, 290.1 to 283.5 mya	Australia, Vasse Shelf	Sue Coal Measures, Seam J		1	48.4	286.8	290	285
Data from: Glasspool and Scott, 2010	Artinskian, 290.1 to 283.5 mya	Australia, Vasse Shelf	Sue Coal Measures, Seam K		1	45.7	286.8	290	285
Data from: Glasspool and Scott, 2010	Artinskian, 290.1 to 283.5 mya	Australia, Vasse Shelf	Sue Coal Measures, Seam L		1	51.5	286.8	290	285
Data from: Glasspool and Scott, 2010	Artinskian, 290.1 to 283.5 mya	Australia, Vasse Shelf	Sue Coal Measures, Seam M		1	40.9	286.8	290	285
Data from: Glasspool and Scott, 2010	Artinskian, 290.1 to 283.5 mya	Australia, Vasse Shelf	Sue Coal Measures, Seam N		1	47.3	286.8	290	285
Data from: Glasspool and Scott, 2010	Artinskian, 290.1 to 283.5 mya	Australia, Vasse Shelf	Sue Coal Measures, Seam O		1	41.8	286.8	290	285
Data from: Glasspool and Scott, 2010	Artinskian, 290.1 to 283.5 mya	Australia, Vasse Shelf	Sue Coal Measures, Seam P		1	58.0	286.8	290	285
Tewalt et al., 2010	Artinskian, 290.1 to 283.5 mya	Botswana, Morupule Coal Basin/Coal Field	Permian, Artinskian, Karoo Super Group, Morupule Fm, Sample # Morupule 0.00-0.95 CH		1	89.7	286.8	290	285
Tewalt et al., 2010	Artinskian, 290.1 to 283.5 mya	Botswana, Morupule Coal Basin/Coal Field	Permian, Artinskian, Karoo Super Group, Morupule Fm, Sample # Morupule 0.95-1.55 CH		1	88.2	286.8	290	285
Tewalt et al., 2010	Artinskian, 290.1 to 283.5 mya	Botswana, Morupule Coal Basin/Coal Field	Permian, Artinskian, Karoo Super Group, Morupule Fm, Sample # Morupule 1.55-2.10 CH		1	91.5	286.8	290	285
Tewalt et al., 2010	Artinskian, 290.1 to 283.5 mya	Botswana, Morupule Coal Basin/Coal Field	Permian, Artinskian, Karoo Super Group, Morupule Fm, Sample # Morupule 2.10-2.85 CH		1	84.2	286.8	290	285
Tewalt et al., 2010	Artinskian, 290.1 to 283.5 mya	Botswana, Morupule Coal Basin/Coal Field	Permian, Artinskian, Karoo Super Group, Morupule Fm, Sample # Morupule ROM 1A		1	86.9	286.8	290	285
Tewalt et al., 2010	Artinskian, 290.1 to 283.5 mya	Botswana, Morupule Coal Basin/Coal Field	Permian, Artinskian, Karoo Super Group, Morupule Fm, Sample # Morupule ROM 1B		1	84.0	286.8	290	285
Tewalt et al., 2010	Artinskian, 290.1 to 283.5 mya	Botswana, Morupule Coal Basin/Coal Field	Permian, Artinskian, Karoo Super Group, Morupule Fm, Sample # Morupule ROM 2A		1	89.1	286.8	290	285
Tewalt et al., 2010	Artinskian, 290.1 to 283.5 mya	Botswana, Morupule Coal Basin/Coal Field	Permian, Artinskian, Karoo Super Group, Morupule Fm, Sample # Morupule ROM 2B		1	89.1	286.8	290	285
Tewalt et al., 2010	Artinskian, 290.1 to 283.5 mya	Botswana, Morupule Coal Basin/Coal Field	Permian, Artinskian, Karoo Super Group, Morupule Fm, Sample # Morupule ROM 2C		1	88.5	286.8	290	285

Tewalt et al., 2010	Artinskian, 290.1 to 283.5 mya	Botswana, Morupule Coal Basin/Coal Field	Permian, Artinskian, Karoo Super Group, Morupule Fm, Sample # Morupule ROM 3A		1	86.3	286.8	290	285
Tewalt et al., 2010	Artinskian, 290.1 to 283.5 mya	Botswana, Morupule Coal Basin/Coal Field	Permian, Artinskian, Karoo Super Group, Morupule Fm, Sample # Morupule ROM 3B		1	91.6	286.8	290	285
Data from: Glasspool and Scott, 2010	Artinskian, 290.1 to 283.5 mya	China	Shanxi Fm	Unclear how many seams	66	36.1	286.8	290	285
Data from: Glasspool and Scott, 2010	Artinskian, 290.1 to 283.5 mya	China	Shanxi Fm, Seam 3		2	23.8	286.8	290	285
Data from: Glasspool and Scott, 2010	Artinskian, 290.1 to 283.5 mya	China	Shanxi Fm, Seam 4		2	18.4	286.8	290	285
Data from: Glasspool and Scott, 2010	Artinskian, 290.1 to 283.5 mya	China, Shandong Province, Yanzhou Mining Area	Shanxi Fm, Seam 3/1		1	26.5	286.8	290	285
Data from: Glasspool and Scott, 2010	Artinskian, 290.1 to 283.5 mya	China, Shandong Province, Yanzhou Mining Area	Shanxi Fm, Seam 3/2		1	18.8	286.8	290	285
Data from: Glasspool and Scott, 2010	Artinskian, 290.1 to 283.5 mya	China, West Shandong Province	Shanxi Fm, Seam 2		2	19.4	286.8	290	285
Data from: Glasspool and Scott, 2010	Artinskian, 290.1 to 283.5 mya	China, Xingtai, Dongpang Mine	Shanxi Fm, Seam 2		1	45.0	286.8	290	285
Tewalt et al., 2010	Artinskian, 290.1 to 283.5 mya	Mozambique, Tete, Moatize Coal Basin/Coal Field, Chipanga XI Mine	Permian, Artinskian, Karoo Super Group, Sample # WOCQI MZ-1 + coal from Moatize		1	24.6	286.8	290	285
Data from: Glasspool and Scott, 2010	Artinskian, 290.1 to 283.5 mya	South Africa, Waterberg	Waterberg Fm	Middle Ecca	1	72.0	286.8	290	285
Smyth, 1972	Sakmarian-Artinskian, 295.0 to 283.5	Australia, Brolga No. 1 Well Cooper Basin	Patchawarra Fm	Table 3.8: 2724.0m		8.0	289.3	290	285
Smyth, 1972	Sakmarian-Artinskian, 295.0 to 283.5	Australia, Brolga No. 1 Well Cooper Basin	Patchawarra Fm	Table 3.8: 2817.3-2819.7m		73.0	289.3	290	285
Smyth, 1972	Sakmarian-Artinskian, 295.0 to 283.5	Australia, Brolga No. 1 Well Cooper Basin	Patchawarra Fm	Table 3.8: 2821.8m		64.0	289.3	290	285
Smyth, 1972	Sakmarian-Artinskian, 295.0 to 283.5	Australia, Brolga No. 1 Well Cooper Basin	Patchawarra Fm	Table 3.8: 2826.7-2830.4m		77.0	289.3	290	285
Smyth, 1972	Sakmarian-Artinskian, 295.0 to 283.5	Australia, Brolga No. 1 Well Cooper Basin	Patchawarra Fm	Table 3.8: 2831.7m		37.0	289.3	290	285
Smyth, 1972	Sakmarian-Artinskian, 295.0 to 283.5	Australia, Brolga No. 1 Well Cooper Basin	Patchawarra Fm	Table 3.8: 2842.9m		64.0	289.3	290	285
Smyth, 1972	Sakmarian-Artinskian, 295.0 to 283.5	Australia, Brolga No. 1 Well Cooper Basin	Patchawarra Fm	Table 3.8: 2844.1m		81.0	289.3	290	285
Smyth, 1972	Sakmarian-Artinskian, 295.0 to 283.5	Australia, Fly Lake No. 1 Well Cooper Basin	Patchawarra Fm	Table 3.5: 2582.1-2584.6m		76.0	289.3	290	285
Smyth, 1972	Sakmarian-Artinskian, 295.0 to 283.5	Australia, Fly Lake No. 1 Well Cooper Basin	Patchawarra Fm	Table 3.5: 2682.7-2684.8m		54.0	289.3	290	285
Smyth, 1972	Sakmarian-Artinskian, 295.0 to 283.5	Australia, Fly Lake No. 1 Well Cooper Basin	Patchawarra Fm	Table 3.5: 2690.8-2692.3m		42.0	289.3	290	285
Smyth, 1972	Sakmarian-Artinskian, 295.0 to 283.5	Australia, Fly Lake No. 1 Well Cooper Basin	Patchawarra Fm	Table 3.5: 2697.5-2702.1m		70.0	289.3	290	285

Smyth, 1972	Sakmarian-Artinskian, 295.0 to 283.5	Australia, Fly Lake No. 1 Well Cooper Basin	Patchawarra Fm	Table 3.5: 2729.8-2744.4m		74.0	289.3	290	285
Smyth, 1972	Sakmarian-Artinskian, 295.0 to 283.5	Australia, Fly Lake No. 1 Well Cooper Basin	Patchawarra Fm	Table 3.5: 2841.3-2842.9m		87.0	289.3	290	285
Smyth, 1972	Sakmarian-Artinskian, 295.0 to 283.5	Australia, Fly Lake No. 2 Well Cooper Basin	Patchawarra Fm	Table 3.6: 2687.7m		21.0	289.3	290	285
Smyth, 1972	Sakmarian-Artinskian, 295.0 to 283.5	Australia, Fly Lake No. 2 Well Cooper Basin	Patchawarra Fm	Table 3.6: 2696.1-2696.3m		17.0	289.3	290	285
Smyth, 1972	Sakmarian-Artinskian, 295.0 to 283.5	Australia, Fly Lake No. 2 Well Cooper Basin	Patchawarra Fm	Table 3.6: 2702.2-2702.7m		47.0	289.3	290	285
Smyth, 1972	Sakmarian-Artinskian, 295.0 to 283.5	Australia, Fly Lake No. 2 Well Cooper Basin	Patchawarra Fm	Table 3.6: 2748.1.2-2749.3m		38.0	289.3	290	285
Smyth, 1972	Sakmarian-Artinskian, 295.0 to 283.5	Australia, Fly Lake No. 2 Well Cooper Basin	Patchawarra Fm	Table 3.6: 2805.1-2806.3m		68.0	289.3	290	285
Smyth, 1972	Sakmarian-Artinskian, 295.0 to 283.5	Australia, Fly Lake No. 2 Well Cooper Basin	Patchawarra Fm	Table 3.6: 2812.2m		77.0	289.3	290	285
Smyth, 1972	Sakmarian-Artinskian, 295.0 to 283.5	Australia, Fly Lake No. 2 Well Cooper Basin	Patchawarra Fm	Table 3.6: 2821.2-2822.1m		79.0	289.3	290	285
Smyth, 1972	Sakmarian-Artinskian, 295.0 to 283.5	Australia, Fly Lake No. 2 Well Cooper Basin	Patchawarra Fm	Table 3.6: 2821.2m		15.0	289.3	290	285
Smyth, 1972	Sakmarian-Artinskian, 295.0 to 283.5	Australia, Fly Lake No. 2 Well Cooper Basin	Patchawarra Fm	Table 3.6: 2831.3m		34.0	289.3	290	285
Smyth, 1972	Sakmarian-Artinskian, 295.0 to 283.5	Australia, Fly Lake No. 2 Well Cooper Basin	Patchawarra Fm	Table 3.6: 2834.5m		61.0	289.3	290	285
Smyth, 1972	Sakmarian-Artinskian, 295.0 to 283.5	Australia, Fly Lake No. 2 Well Cooper Basin	Patchawarra Fm	Table 3.6: 2839.5m		19.0	289.3	290	285
Smyth, 1972	Sakmarian-Artinskian, 295.0 to 283.5	Australia, Fly Lake No. 2 Well Cooper Basin	Patchawarra Fm	Table 3.6: 2890.1m		46.0	289.3	290	285
Smyth, 1972	Sakmarian-Artinskian, 295.0 to 283.5	Australia, Fly Lake No. 2 Well Cooper Basin	Patchawarra Fm	Table 3.6: 2891.3-2892.4m		59.0	289.3	290	285
Smyth, 1972	Sakmarian-Artinskian, 295.0 to 283.5	Australia, Fly Lake No. 2 Well Cooper Basin	Patchawarra Fm	Table 3.6: 2899.9m		83.0	289.3	290	285
Smyth, 1972	Sakmarian-Artinskian, 295.0 to 283.5	Australia, Fly Lake No. 2 Well Cooper Basin	Patchawarra Fm	Table 3.6: 2902.0-2902.3m		86.0	289.3	290	285
Smyth, 1972	Sakmarian-Artinskian, 295.0 to 283.5	Australia, Fly Lake No. 2 Well Cooper Basin	Patchawarra Fm	Table 3.6: 2903.5m		72.0	289.3	290	285
Smyth, 1972	Sakmarian-Artinskian, 295.0 to 283.5	Australia, Fly Lake No. 3 Well Cooper Basin	Patchawarra Fm	Table 3.7: 2659.4-2660.0m		48.0	289.3	290	285
Smyth, 1972	Sakmarian-Artinskian, 295.0 to 283.5	Australia, Fly Lake No. 3 Well Cooper Basin	Patchawarra Fm	Table 3.7: 2669.7-2670.0m		5.0	289.3	290	285
Smyth, 1972	Sakmarian-Artinskian, 295.0 to 283.5	Australia, Fly Lake No. 3 Well Cooper Basin	Patchawarra Fm	Table 3.7: 2676.0m		26.0	289.3	290	285
Smyth, 1972	Sakmarian-Artinskian, 295.0 to 283.5	Australia, Fly Lake No. 3 Well Cooper Basin	Patchawarra Fm	Table 3.7: 2681.0-2682.5m		62.0	289.3	290	285
Smyth, 1972	Sakmarian-Artinskian, 295.0 to 283.5	Australia, Fly Lake No. 3 Well Cooper Basin	Patchawarra Fm	Table 3.7: 2805.4-2806.0m		39.0	289.3	290	285
Smyth, 1972	Sakmarian-Artinskian, 295.0 to 283.5	Australia, Fly Lake No. 3 Well Cooper Basin	Patchawarra Fm	Table 3.7: 2840.4m		39.0	289.3	290	285
Smyth, 1972	Sakmarian-Artinskian, 295.0 to 283.5	Australia, Fly Lake No. 3 Well Cooper Basin	Patchawarra Fm	Table 3.7: 2847.7-2848.7m		58.0	289.3	290	285

Smyth, 1972	Sakmarian-Artinskian, 295.0 to 283.5	Australia, Fly Lake No. 3 Well Cooper Basin	Patchawarra Fm	Table 3.7: 2854.5m		74.0	289.3	290	285
Smyth, 1972	Sakmarian-Artinskian, 295.0 to 283.5	Australia, Fly Lake No. 3 Well Cooper Basin	Patchawarra Fm	Table 3.7: 2856.9m		29.0	289.3	290	285
Smyth, 1972	Sakmarian-Artinskian, 295.0 to 283.5	Australia, Fly Lake No. 3 Well Cooper Basin	Patchawarra Fm	Table 3.7: 2873.3-2874.3m		49.0	289.3	290	285
Smyth, 1972	Sakmarian-Artinskian, 295.0 to 283.5	Australia, Fly Lake No. 3 Well Cooper Basin	Patchawarra Fm	Table 3.7: 2887.3m		84.0	289.3	290	285
Smyth, 1972	Sakmarian-Artinskian, 295.0 to 283.5	Australia, Fly Lake No. 3 Well Cooper Basin	Patchawarra Fm	Table 3.7: 2887.4m		64.0	289.3	290	285
Smyth, 1972	Sakmarian-Artinskian, 295.0 to 283.5	Australia, Fly Lake No. 3 Well Cooper Basin	Patchawarra Fm	Table 3.7: 2897.7-2898.3m		48.0	289.3	290	285
Smyth, 1972	Sakmarian-Artinskian, 295.0 to 283.5	Australia, Fly Lake No. 3 Well Cooper Basin	Patchawarra Fm	Table 3.7: 2901.4m		65.0	289.3	290	285
Smyth, 1972	Sakmarian-Artinskian, 295.0 to 283.5	Australia, Fly Lake No. 3 Well Cooper Basin	Patchawarra Fm	Table 3.7: 2903.5-2904.7m		58.0	289.3	290	285
Smyth, 1972	Sakmarian-Artinskian, 295.0 to 283.5	Australia, Fly Lake No. 3 Well Cooper Basin	Patchawarra Fm	Table 3.7: 2908.7-2909.6m		77.0	289.3	290	285
Smyth, 1972	Sakmarian-Artinskian, 295.0 to 283.5	Australia, Fly Lake No. 3 Well Cooper Basin	Patchawarra Fm	Table 3.7: 2919.1-2920.0m		34.0	289.3	290	285
Smyth, 1972	Sakmarian-Artinskian, 295.0 to 283.5	Australia, Mudrangie No. 1 Well, Cooper Basin, Patchawarra Trough	Patchawarra Fm	Table 4.4: 2813.3-2816.4m		41.0	289.3	290	285
Smyth, 1972	Sakmarian-Artinskian, 295.0 to 283.5	Australia, Mudrangie No. 1 Well, Cooper Basin, Patchawarra Trough	Patchawarra Fm	Table 4.4: 2825.5-2828.5m		33.0	289.3	290	285
Smyth, 1972	Sakmarian-Artinskian, 295.0 to 283.5	Australia, Mudrangie No. 1 Well, Cooper Basin, Patchawarra Trough	Patchawarra Fm	Table 4.4: 2837.7-2840.7m		57.0	289.3	290	285
Smyth, 1972	Sakmarian-Artinskian, 295.0 to 283.5	Australia, Mudrangie No. 1 Well, Cooper Basin, Patchawarra Trough	Patchawarra Fm	Table 4.4: 2856.0-2859.0m		57.0	289.3	290	285
Smyth, 1972	Sakmarian-Artinskian, 295.0 to 283.5	Australia, Mudrangie No. 1 Well, Cooper Basin, Patchawarra Trough	Patchawarra Fm	Table 4.6: 2856.0-2862.1m		58.0	289.3	290	285
Smyth, 1972	Sakmarian-Artinskian, 295.0 to 283.5	Australia, Mudrangie No. 1 Well, Cooper Basin, Patchawarra Trough	Patchawarra Fm	Table 4.4: 2859.0-2862.1m		60.0	289.3	290	285
Smyth, 1972	Sakmarian-Artinskian, 295.0 to 283.5	Australia, Mudrangie No. 1 Well, Cooper Basin, Patchawarra Trough	Patchawarra Fm	Table 4.4: 2862.1-2865.1m		67.0	289.3	290	285
Smyth, 1972	Sakmarian-Artinskian, 295.0 to 283.5	Australia, Mudrangie No. 1 Well, Cooper Basin, Patchawarra Trough	Patchawarra Fm	Table 4.4: 2865.1-2868.2m		75.0	289.3	290	285
Smyth, 1972	Sakmarian-Artinskian, 295.0 to 283.5	Australia, Mudrangie No. 1 Well, Cooper Basin, Patchawarra Trough	Patchawarra Fm	Table 4.4: 2868.2-2871.2m		61.0	289.3	290	285
Smyth, 1972	Sakmarian-Artinskian, 295.0 to 283.5	Australia, Mudrangie No. 1 Well, Cooper Basin, Patchawarra Trough	Patchawarra Fm	Table 4.6: 2868.2-2874.3m		62.0	289.3	290	285
Smyth, 1972	Sakmarian-Artinskian, 295.0 to 283.5	Australia, Mudrangie No. 1 Well, Cooper Basin, Patchawarra Trough	Patchawarra Fm	Table 4.4: 2871.2-2874.3m		63.0	289.3	290	285
Smyth, 1972	Sakmarian-Artinskian, 295.0 to 283.5	Australia, Mudrangie No. 1 Well, Cooper	Patchawarra Fm	Table 4.4: 2889.5-2892.6m		66.0	289.3	290	285

		Basin, Patchawarra Trough							
Smyth, 1972	Sakmarian-Artinskian, 295.0 to 283.5	Australia, Mudrangie No. 1 Well, Cooper Basin, Patchawarra Trough	Patchawarra Fm	Table 4.4: 2892.6-2895.6m		83.0	289.3	290	285
Smyth, 1972	Sakmarian-Artinskian, 295.0 to 283.5	Australia, Mudrangie No. 1 Well, Cooper Basin, Patchawarra Trough	Patchawarra Fm	Table 4.4: 2895.6-2898.6m		62.0	289.3	290	285
Smyth, 1972	Sakmarian-Artinskian, 295.0 to 283.5	Australia, Mudrangie No. 1 Well, Cooper Basin, Patchawarra Trough	Patchawarra Fm	Table 4.6: 2895.6-2898.6m		62.0	289.3	290	285
Smyth, 1972	Sakmarian-Artinskian, 295.0 to 283.5	Australia, Mudrangie No. 1 Well, Cooper Basin, Patchawarra Trough	Patchawarra Fm	Table 4.4: 2913.6-2916.9m		72.0	289.3	290	285
Smyth, 1972	Sakmarian-Artinskian, 295.0 to 283.5	Australia, Mudrangie No. 1 Well, Cooper Basin, Patchawarra Trough	Patchawarra Fm	Table 4.6: 2913.6-2920.0m		72.0	289.3	290	285
Smyth, 1972	Sakmarian-Artinskian, 295.0 to 283.5	Australia, Mudrangie No. 1 Well, Cooper Basin, Patchawarra Trough	Patchawarra Fm	Table 4.4: 2916.9-2920.0m		58.0	289.3	290	285
Smyth, 1972	Sakmarian-Artinskian, 295.0 to 283.5	Australia, Mudrangie No. 1 Well, Cooper Basin, Patchawarra Trough	Patchawarra Fm	Table 4.4: 2920.0-2923.0m		50.0	289.3	290	285
Smyth, 1972	Sakmarian-Artinskian, 295.0 to 283.5	Australia, Mudrangie No. 1 Well, Cooper Basin, Patchawarra Trough	Patchawarra Fm	Table 4.4: 2926.1-2929.1m		71.0	289.3	290	285
Smyth, 1972	Sakmarian-Artinskian, 295.0 to 283.5	Australia, Mudrangie No. 1 Well, Cooper Basin, Patchawarra Trough	Patchawarra Fm	Table 4.6: 2926.1-2941.3m		78.0	289.3	290	285
Smyth, 1972	Sakmarian-Artinskian, 295.0 to 283.5	Australia, Mudrangie No. 1 Well, Cooper Basin, Patchawarra Trough	Patchawarra Fm	Table 4.4: 2929.1-2932.2m		80.0	289.3	290	285
Smyth, 1972	Sakmarian-Artinskian, 295.0 to 283.5	Australia, Mudrangie No. 1 Well, Cooper Basin, Patchawarra Trough	Patchawarra Fm	Table 4.4: 2932.2-2935.2m		79.0	289.3	290	285
Smyth, 1972	Sakmarian-Artinskian, 295.0 to 283.5	Australia, Mudrangie No. 1 Well, Cooper Basin, Patchawarra Trough	Patchawarra Fm	Table 4.4: 2935.2-2938.3m		73.0	289.3	290	285
Smyth, 1972	Sakmarian-Artinskian, 295.0 to 283.5	Australia, Mudrangie No. 1 Well, Cooper Basin, Patchawarra Trough	Patchawarra Fm	Table 4.4: 2938.3-2941.3m		73.0	289.3	290	285
Smyth, 1972	Sakmarian-Artinskian, 295.0 to 283.5	Australia, Mudrangie No. 1 Well, Cooper Basin, Patchawarra Trough	Patchawarra Fm	Table 4.4: 2941.3-2944.4m		81.0	289.3	290	285
Smyth, 1972	Sakmarian-Artinskian, 295.0 to 283.5	Australia, Mudrangie No. 1 Well, Cooper Basin, Patchawarra Trough	Patchawarra Fm	Table 4.4: 2947.4-2596.6m		93.0	289.3	290	285
Smyth, 1972	Sakmarian-Artinskian, 295.0 to 283.5	Australia, Mudrangie No. 1 Well, Cooper Basin, Patchawarra Trough	Patchawarra Fm	Table 4.4: 2959.6-2962.7m		92.0	289.3	290	285
Smyth, 1972	Sakmarian-Artinskian, 295.0 to 283.5	Australia, Mudrangie No. 1 Well, Cooper Basin, Patchawarra Trough	Patchawarra Fm	Table 4.4: 2968.8-2971.8m		60.0	289.3	290	285
Smyth, 1972	Sakmarian-Artinskian, 295.0 to 283.5	Australia, Mudrangie No. 1 Well, Cooper Basin, Patchawarra Trough	Patchawarra Fm	Table 4.4: 2977.9-2984.0m		91.0	289.3	290	285
Smyth, 1972	Sakmarian-Artinskian, 295.0 to 283.5	Australia, Mudrangie No. 1 Well, Cooper	Patchawarra Fm	Table 4.4: 2993.1-2996.2m		57.0	289.3	290	285

		Basin, Patchawarra Trough							
Smyth, 1972	Sakmarian-Artinskian, 295.0 to 283.5	Australia, Mudrangie No. 1 Well, Cooper Basin, Patchawarra Trough	Patchawarra Fm	Table 4.4: 2999.2-3002.3m		100.0	289.3	290	285
Smyth, 1972	Sakmarian-Artinskian, 295.0 to 283.5	Australia, Mudrangie No. 1 Well, Cooper Basin, Patchawarra Trough	Patchawarra Fm	Table 4.4: 3008.4-3011.4m		50.0	289.3	290	285
Smyth, 1972	Sakmarian-Artinskian, 295.0 to 283.5	Australia, Mudrangie No. 1 Well, Cooper Basin, Patchawarra Trough	Patchawarra Fm	Table 4.4: 3017.5-3020.6m		44.0	289.3	290	285
Smyth, 1972	Sakmarian-Artinskian, 295.0 to 283.5	Australia, Mudrangie No. 1 Well, Cooper Basin, Patchawarra Trough	Patchawarra Fm	Table 4.6: 3017.5-3020.6m		44.0	289.3	290	285
Smyth, 1972	Sakmarian-Artinskian, 295.0 to 283.5	Australia, Mudrangie No. 1 Well, Cooper Basin, Patchawarra Trough	Patchawarra Fm	Table 4.4: 3026.7-3029.7m		66.0	289.3	290	285
Smyth, 1972	Sakmarian-Artinskian, 295.0 to 283.5	Australia, Mudrangie No. 1 Well, Cooper Basin, Patchawarra Trough	Patchawarra Fm	Table 4.4: 3038.9-3041.9m		61.0	289.3	290	285
Smyth, 1972	Sakmarian-Artinskian, 295.0 to 283.5	Australia, Mudrangie No. 1 Well, Cooper Basin, Patchawarra Trough	Patchawarra Fm	Table 4.4: 3051.0-3054.1m		80.0	289.3	290	285
Smyth, 1972	Sakmarian-Artinskian, 295.0 to 283.5	Australia, Mudrangie No. 1 Well, Cooper Basin, Patchawarra Trough	Patchawarra Fm	Table 4.4: 3057.1-3060.2m		79.0	289.3	290	285
Smyth, 1972	Sakmarian-Artinskian, 295.0 to 283.5	Australia, Mudrangie No. 1 Well, Cooper Basin, Patchawarra Trough	Patchawarra Fm	Table 4.4: 3069.3-3072.4m		73.0	289.3	290	285
Smyth, 1972	Sakmarian-Artinskian, 295.0 to 283.5	Australia, Mudrangie No. 1 Well, Cooper Basin, Patchawarra Trough	Patchawarra Fm	Table 4.6: 3069.3-3072.4m		73.0	289.3	290	285
Smyth, 1972	Sakmarian-Artinskian, 295.0 to 283.5	Australia, Mudrangie No. 1 Well, Cooper Basin, Patchawarra Trough	Patchawarra Fm	Table 4.6: 2856.0-2862.1m: Patchawarra 1 Coal Seam		58.0	289.3	290	285
Smyth, 1972	Sakmarian-Artinskian, 295.0 to 283.5	Australia, Mudrangie No. 1 Well, Cooper Basin, Patchawarra Trough	Patchawarra Fm	Table 4.6: 2868.2-2874.3m: Patchawarra 2 Coal Seam		62.0	289.3	290	285
Smyth, 1972	Sakmarian-Artinskian, 295.0 to 283.5	Australia, Mudrangie No. 1 Well, Cooper Basin, Patchawarra Trough	Patchawarra Fm	Table 4.6: 2895.6-2898.6m: Patchawarra 3 Coal Seam		62.0	289.3	290	285
Smyth, 1972	Sakmarian-Artinskian, 295.0 to 283.5	Australia, Mudrangie No. 1 Well, Cooper Basin, Patchawarra Trough	Patchawarra Fm	Table 4.6: 2913.6-2920.0m: Patchawarra 4 Coal Seam		72.0	289.3	290	285
Smyth, 1972	Sakmarian-Artinskian, 295.0 to 283.5	Australia, Mudrangie No. 1 Well, Cooper Basin, Patchawarra Trough	Patchawarra Fm	Table 4.6: 2926.1-2941.3m: Patchawarra 5 Coal Seam (Malabine Coal)		78.0	289.3	290	285
Smyth, 1972	Sakmarian-Artinskian, 295.0 to 283.5	Australia, Mudrangie No. 1 Well, Cooper Basin, Patchawarra Trough	Patchawarra Fm	Table 4.6: 3017.5-3020.6m: Patchawarra 6 Coal Seam		44.0	289.3	290	285
Smyth, 1972	Sakmarian-Artinskian, 295.0 to 283.5	Australia, Mudrangie No. 1 Well, Cooper Basin, Patchawarra Trough	Patchawarra Fm	Table 4.6: 3069.3-3072.4m: Patchawarra 7 Coal Seam		73.0	289.3	290	285
Huleatt, 1991	Sakmarian-Artinskian, 295.0 to 283.5	Australia, SW of Capella	Reids Dome Beds, Anakie		1	25.0	289.3	290	285
Huleatt, 1991	Sakmarian-Artinskian, 295.0 to 283.5	Australia, SW of Capella	Reids Dome Beds, Kettle		1	36.0	289.3	290	285

Huleatt, 1991	Sakmarian-Artinskian, 295.0 to 283.5	Australia, SW of Capella	Reids Dome Beds, La Poule		1	49.0	289.3	290	285
Huleatt, 1991	Sakmarian-Artinskian, 295.0 to 283.5	Australia, SW of Emerald, Cullin-la-ringo area, Cullin-la-ringo Prospect	Reids Dome Beds, unnamed seam		3	40.8	289.3	290	285
Huleatt, 1991	Sakmarian-Artinskian, 295.0 to 283.5	Australia, SW of Emerald, Cullin-la-ringo area, Gindie Prospect	Reids Dome Beds, unnamed seam		3	29.8	289.3	290	285
Smyth, 1972	Sakmarian-Artinskian, 295.0 to 283.5	Australia, Tindilpie No. 1 Well, Cooper Basin, Patchawarra Trough	Patchawarra Fm	Table 4.15: 10050ft		80.0	289.3	290	285
Smyth, 1972	Sakmarian-Artinskian, 295.0 to 283.5	Australia, Tindilpie No. 1 Well, Cooper Basin, Patchawarra Trough	Patchawarra Fm	Table 4.15: 10070ft		72.0	289.3	290	285
Smyth, 1972	Sakmarian-Artinskian, 295.0 to 283.5	Australia, Tindilpie No. 1 Well, Cooper Basin, Patchawarra Trough	Patchawarra Fm	Table 4.15: 10090ft		74.0	289.3	290	285
Smyth, 1972	Sakmarian-Artinskian, 295.0 to 283.5	Australia, Tindilpie No. 1 Well, Cooper Basin, Patchawarra Trough	Patchawarra Fm	Table 4.15: 10120ft		62.0	289.3	290	285
Smyth, 1972	Sakmarian-Artinskian, 295.0 to 283.5	Australia, Tindilpie No. 1 Well, Cooper Basin, Patchawarra Trough	Patchawarra Fm	Table 4.15: 10130ft		72.0	289.3	290	285
Smyth, 1972	Sakmarian-Artinskian, 295.0 to 283.5	Australia, Tindilpie No. 1 Well, Cooper Basin, Patchawarra Trough	Patchawarra Fm	Table 4.15: 10140ft		67.0	289.3	290	285
Smyth, 1972	Sakmarian-Artinskian, 295.0 to 283.5	Australia, Tindilpie No. 1 Well, Cooper Basin, Patchawarra Trough	Patchawarra Fm	Table 4.15: 10160ft		69.0	289.3	290	285
Smyth, 1972	Sakmarian-Artinskian, 295.0 to 283.5	Australia, Tindilpie No. 1 Well, Cooper Basin, Patchawarra Trough	Patchawarra Fm	Table 4.15: 10170ft		80.0	289.3	290	285
Smyth, 1972	Sakmarian-Artinskian, 295.0 to 283.5	Australia, Tindilpie No. 1 Well, Cooper Basin, Patchawarra Trough	Patchawarra Fm	Table 4.15: 10200ft		69.0	289.3	290	285
Smyth, 1972	Sakmarian-Artinskian, 295.0 to 283.5	Australia, Tindilpie No. 1 Well, Cooper Basin, Patchawarra Trough	Patchawarra Fm	Table 4.15: 10250ft		61.0	289.3	290	285
Smyth, 1972	Sakmarian-Artinskian, 295.0 to 283.5	Australia, Tindilpie No. 1 Well, Cooper Basin, Patchawarra Trough	Patchawarra Fm	Table 4.15: 10270ft		72.0	289.3	290	285
Smyth, 1972	Sakmarian-Artinskian, 295.0 to 283.5	Australia, Tindilpie No. 1 Well, Cooper Basin, Patchawarra Trough	Patchawarra Fm	Table 4.15: 10320ft		65.0	289.3	290	285
Smyth, 1972	Sakmarian-Artinskian, 295.0 to 283.5	Australia, Tindilpie No. 1 Well, Cooper Basin, Patchawarra Trough	Patchawarra Fm	Table 4.15: 10330ft		66.0	289.3	290	285
Smyth, 1972	Sakmarian-Artinskian, 295.0 to 283.5	Australia, Tindilpie No. 1 Well, Cooper Basin, Patchawarra Trough	Patchawarra Fm	Table 4.15: 10350ft		68.0	289.3	290	285
Smyth, 1972	Sakmarian-Artinskian, 295.0 to 283.5	Australia, Tindilpie No. 1 Well, Cooper Basin, Patchawarra Trough	Patchawarra Fm	Table 4.15: 10390ft		62.0	289.3	290	285
Smyth, 1972	Sakmarian-Artinskian, 295.0 to 283.5	Australia, Tindilpie No. 1 Well, Cooper Basin, Patchawarra Trough	Patchawarra Fm	Table 4.15: 10430ft		54.0	289.3	290	285

Smyth, 1972	Sakmarian-Artinskian, 295.0 to 283.5	Australia, Tindilpie No. 1 Well, Cooper Basin, Patchawarra Trough	Patchawarra Fm	Table 4.15: 10450ft		76.0	289.3	290	285
Smyth, 1972	Sakmarian-Artinskian, 295.0 to 283.5	Australia, Tindilpie No. 1 Well, Cooper Basin, Patchawarra Trough	Patchawarra Fm	Table 4.15: 10470ft		74.0	289.3	290	285
Smyth, 1972	Sakmarian-Artinskian, 295.0 to 283.5	Australia, Tindilpie No. 1 Well, Cooper Basin, Patchawarra Trough	Patchawarra Fm	Table 4.15: 10490ft		66.0	289.3	290	285
Smyth, 1972	Sakmarian-Artinskian, 295.0 to 283.5	Australia, Tindilpie No. 1 Well, Cooper Basin, Patchawarra Trough	Patchawarra Fm	Table 4.15: 10530ft		68.0	289.3	290	285
Smyth, 1972	Sakmarian-Artinskian, 295.0 to 283.5	Australia, Tindilpie No. 1 Well, Cooper Basin, Patchawarra Trough	Patchawarra Fm	Table 4.15: 9030ft		39.0	289.3	290	285
Smyth, 1972	Sakmarian-Artinskian, 295.0 to 283.5	Australia, Tindilpie No. 1 Well, Cooper Basin, Patchawarra Trough	Patchawarra Fm	Table 4.15: 9080ft		53.0	289.3	290	285
Smyth, 1972	Sakmarian-Artinskian, 295.0 to 283.5	Australia, Tindilpie No. 1 Well, Cooper Basin, Patchawarra Trough	Patchawarra Fm	Table 4.15: 9130ft		34.0	289.3	290	285
Smyth, 1972	Sakmarian-Artinskian, 295.0 to 283.5	Australia, Tindilpie No. 1 Well, Cooper Basin, Patchawarra Trough	Patchawarra Fm	Table 4.15: 9150ft		35.0	289.3	290	285
Smyth, 1972	Sakmarian-Artinskian, 295.0 to 283.5	Australia, Tindilpie No. 1 Well, Cooper Basin, Patchawarra Trough	Patchawarra Fm	Table 4.15: 9190ft		39.0	289.3	290	285
Smyth, 1972	Sakmarian-Artinskian, 295.0 to 283.5	Australia, Tindilpie No. 1 Well, Cooper Basin, Patchawarra Trough	Patchawarra Fm	Table 4.15: 9220ft		62.0	289.3	290	285
Smyth, 1972	Sakmarian-Artinskian, 295.0 to 283.5	Australia, Tindilpie No. 1 Well, Cooper Basin, Patchawarra Trough	Patchawarra Fm	Table 4.15: 9230ft		42.0	289.3	290	285
Smyth, 1972	Sakmarian-Artinskian, 295.0 to 283.5	Australia, Tindilpie No. 1 Well, Cooper Basin, Patchawarra Trough	Patchawarra Fm	Table 4.15: 9270ft		35.0	289.3	290	285
Smyth, 1972	Sakmarian-Artinskian, 295.0 to 283.5	Australia, Tindilpie No. 1 Well, Cooper Basin, Patchawarra Trough	Patchawarra Fm	Table 4.15: 9290ft		43.0	289.3	290	285
Smyth, 1972	Sakmarian-Artinskian, 295.0 to 283.5	Australia, Tindilpie No. 1 Well, Cooper Basin, Patchawarra Trough	Patchawarra Fm	Table 4.15: 9300ft		61.0	289.3	290	285
Smyth, 1972	Sakmarian-Artinskian, 295.0 to 283.5	Australia, Tindilpie No. 1 Well, Cooper Basin, Patchawarra Trough	Patchawarra Fm	Table 4.15: 9310ft		46.0	289.3	290	285
Smyth, 1972	Sakmarian-Artinskian, 295.0 to 283.5	Australia, Tindilpie No. 1 Well, Cooper Basin, Patchawarra Trough	Patchawarra Fm	Table 4.15: 9380ft		38.0	289.3	290	285
Smyth, 1972	Sakmarian-Artinskian, 295.0 to 283.5	Australia, Tindilpie No. 1 Well, Cooper Basin, Patchawarra Trough	Patchawarra Fm	Table 4.15: 9420ft		50.0	289.3	290	285
Smyth, 1972	Sakmarian-Artinskian, 295.0 to 283.5	Australia, Tindilpie No. 1 Well, Cooper Basin, Patchawarra Trough	Patchawarra Fm	Table 4.15: 9430ft		55.0	289.3	290	285
Smyth, 1972	Sakmarian-Artinskian, 295.0 to 283.5	Australia, Tindilpie No. 1 Well, Cooper Basin, Patchawarra Trough	Patchawarra Fm	Table 4.15: 9440ft		59.0	289.3	290	285

Smyth, 1972	Sakmarian-Artinskian, 295.0 to 283.5	Australia, Tindilpie No. 1 Well, Cooper Basin, Patchawarra Trough	Patchawarra Fm	Table 4.15: 9450ft		44.0	289.3	290	285
Smyth, 1972	Sakmarian-Artinskian, 295.0 to 283.5	Australia, Tindilpie No. 1 Well, Cooper Basin, Patchawarra Trough	Patchawarra Fm	Table 4.15: 9480ft		54.0	289.3	290	285
Smyth, 1972	Sakmarian-Artinskian, 295.0 to 283.5	Australia, Tindilpie No. 1 Well, Cooper Basin, Patchawarra Trough	Patchawarra Fm	Table 4.15: 9490ft		51.0	289.3	290	285
Smyth, 1972	Sakmarian-Artinskian, 295.0 to 283.5	Australia, Tindilpie No. 1 Well, Cooper Basin, Patchawarra Trough	Patchawarra Fm	Table 4.15: 9510ft		54.0	289.3	290	285
Smyth, 1972	Sakmarian-Artinskian, 295.0 to 283.5	Australia, Tindilpie No. 1 Well, Cooper Basin, Patchawarra Trough	Patchawarra Fm	Table 4.15: 9600ft		65.0	289.3	290	285
Smyth, 1972	Sakmarian-Artinskian, 295.0 to 283.5	Australia, Tindilpie No. 1 Well, Cooper Basin, Patchawarra Trough	Patchawarra Fm	Table 4.15: 9610ft		74.0	289.3	290	285
Smyth, 1972	Sakmarian-Artinskian, 295.0 to 283.5	Australia, Tindilpie No. 1 Well, Cooper Basin, Patchawarra Trough	Patchawarra Fm	Table 4.15: 9620ft		68.0	289.3	290	285
Smyth, 1972	Sakmarian-Artinskian, 295.0 to 283.5	Australia, Tindilpie No. 1 Well, Cooper Basin, Patchawarra Trough	Patchawarra Fm	Table 4.15: 9690ft		65.0	289.3	290	285
Smyth, 1972	Sakmarian-Artinskian, 295.0 to 283.5	Australia, Tindilpie No. 1 Well, Cooper Basin, Patchawarra Trough	Patchawarra Fm	Table 4.15: 9700ft		61.0	289.3	290	285
Smyth, 1972	Sakmarian-Artinskian, 295.0 to 283.5	Australia, Tindilpie No. 1 Well, Cooper Basin, Patchawarra Trough	Patchawarra Fm	Table 4.15: 9710ft		82.0	289.3	290	285
Smyth, 1972	Sakmarian-Artinskian, 295.0 to 283.5	Australia, Tindilpie No. 1 Well, Cooper Basin, Patchawarra Trough	Patchawarra Fm	Table 4.15: 9750ft		59.0	289.3	290	285
Smyth, 1972	Sakmarian-Artinskian, 295.0 to 283.5	Australia, Tindilpie No. 1 Well, Cooper Basin, Patchawarra Trough	Patchawarra Fm	Table 4.15: 9860ft		86.0	289.3	290	285
Smyth, 1972	Sakmarian-Artinskian, 295.0 to 283.5	Australia, Tindilpie No. 1 Well, Cooper Basin, Patchawarra Trough	Patchawarra Fm	Table 4.15: 9870ft		82.0	289.3	290	285
Smyth, 1972	Sakmarian-Artinskian, 295.0 to 283.5	Australia, Tindilpie No. 1 Well, Cooper Basin, Patchawarra Trough	Patchawarra Fm	Table 4.15: 9890ft		60.0	289.3	290	285
Smyth, 1972	Sakmarian-Artinskian, 295.0 to 283.5	Australia, Tindilpie No. 1 Well, Cooper Basin, Patchawarra Trough	Patchawarra Fm	Table 4.15: 9900ft		64.0	289.3	290	285
Smyth, 1972	Sakmarian-Artinskian, 295.0 to 283.5	Australia, Tindilpie No. 1 Well, Cooper Basin, Patchawarra Trough	Patchawarra Fm	Table 4.15: 9920ft		66.0	289.3	290	285
Smyth, 1972	Sakmarian-Artinskian, 295.0 to 283.5	Australia, Tindilpie No. 1 Well, Cooper Basin, Patchawarra Trough	Patchawarra Fm	Table 4.15: 9950ft		75.0	289.3	290	285
Smyth, 1972	Sakmarian-Artinskian, 295.0 to 283.5	Australia, Tindilpie No. 1 Well, Cooper Basin, Patchawarra Trough	Patchawarra Fm	Table 4.15: 9980ft		69.0	289.3	290	300
Data from: Glasspool and Scott, 2010	Middle Sakmarian, 295.0 to 290.1 mya	Brazil	Lower Candiota Seam		1	27.0	292.6	290	300

Data from: Glasspool and Scott, 2010	Middle Sakmarian, 295.0 to 290.1 mya	Brazil	Rio Bonito Fm		1	16.8	292.6	290	300
Data from: Glasspool and Scott, 2010	Middle Sakmarian, 295.0 to 290.1 mya	Brazil	Upper Candiota Seam		1	25.0	292.6	290	300
Data from: Glasspool and Scott, 2010	Middle Sakmarian, 295.0 to 290.1 mya	Brazil, Candiota and Figuiera Coalfields	Rio Bonito Fm	Unclear how many seams	70	30.4	292.6	290	300
Data from: Glasspool and Scott, 2010	Middle Sakmarian, 295.0 to 290.1 mya	Brazil, Candiota Coalfield	Rio Bonito Fm, Camada Banco Louco Seam		1	68.0	292.6	290	300
Data from: Glasspool and Scott, 2010	Middle Sakmarian, 295.0 to 290.1 mya	Brazil, Candiota Coalfield	Rio Bonito Fm, Camada Candiota Inferior Seam		1	46.0	292.6	290	300
Data from: Glasspool and Scott, 2010	Middle Sakmarian, 295.0 to 290.1 mya	Brazil, Candiota Coalfield	Rio Bonito Fm, Camada Candiota Superior Seam		1	38.0	292.6	290	300
Data from: Glasspool and Scott, 2010	Middle Sakmarian, 295.0 to 290.1 mya	Brazil, Candiota Coalfield	Rio Bonito Fm, Camadas Inferiores I1 Seam		1	37.0	292.6	290	300
Data from: Glasspool and Scott, 2010	Middle Sakmarian, 295.0 to 290.1 mya	Brazil, Candiota Coalfield	Rio Bonito Fm, Camadas Inferiores I2 Seam		1	20.0	292.6	290	300
Data from: Glasspool and Scott, 2010	Middle Sakmarian, 295.0 to 290.1 mya	Brazil, Candiota Coalfield	Rio Bonito Fm, Camadas Inferiores I3 Seam		1	40.0	292.6	290	300
Data from: Glasspool and Scott, 2010	Middle Sakmarian, 295.0 to 290.1 mya	Brazil, Candiota Coalfield	Rio Bonito Fm, Camadas Inferiores I4 Seam		1	19.0	292.6	290	300
Data from: Glasspool and Scott, 2010	Middle Sakmarian, 295.0 to 290.1 mya	Brazil, Candiota Coalfield	Rio Bonito Fm, Seam BL	Seam BL	1	56.0	292.6	290	300
Data from: Glasspool and Scott, 2010	Middle Sakmarian, 295.0 to 290.1 mya	Brazil, Candiota Coalfield	Rio Bonito Fm, Seam CCI	Seam CCI	1	34.0	292.6	290	300
Data from: Glasspool and Scott, 2010	Middle Sakmarian, 295.0 to 290.1 mya	Brazil, Candiota Coalfield	Rio Bonito Fm, Seam CCI	Seam CCI	1	28.0	292.6	290	300
Data from: Glasspool and Scott, 2010	Middle Sakmarian, 295.0 to 290.1 mya	Brazil, Candiota Coalfield	Rio Bonito Fm, Seam CCS	Seam CCS	1	24.0	292.6	290	300
Data from: Glasspool and Scott, 2010	Middle Sakmarian, 295.0 to 290.1 mya	Brazil, Candiota Coalfield	Rio Bonito Fm, Seam CCS	Seam CCS	1	31.0	292.6	290	300
Data from: Glasspool and Scott, 2010	Middle Sakmarian, 295.0 to 290.1 mya	Brazil, Candiota Coalfield	Rio Bonito Fm, Seam I1	Seam I1	1	31.0	292.6	290	300
Data from: Glasspool and Scott, 2010	Middle Sakmarian, 295.0 to 290.1 mya	Brazil, Candiota Coalfield	Rio Bonito Fm, Seam I2	Seam I2	1	11.0	292.6	290	300
Data from: Glasspool and Scott, 2010	Middle Sakmarian, 295.0 to 290.1 mya	Brazil, Candiota Coalfield	Rio Bonito Fm, Seam I3	Seam I3	1	27.0	292.6	290	300
Data from: Glasspool and Scott, 2010	Middle Sakmarian, 295.0 to 290.1 mya	Brazil, Candiota Coalfield	Rio Bonito Fm, Seam I4	Seam I4	1	10.0	292.6	290	300
Data from: Glasspool and Scott, 2010	Middle Sakmarian, 295.0 to 290.1 mya	Brazil, Candiota Coalfield	Rio Bonito Fm, Seam I5	Seam I5	1	22.0	292.6	290	300
Data from: Glasspool and Scott, 2010	Middle Sakmarian, 295.0 to 290.1 mya	Brazil, Candiota Coalfield	Rio Bonito Fm, Seam L1	Seam L1	1	51.0	292.6	290	300
Data from: Glasspool and Scott, 2010	Middle Sakmarian, 295.0 to 290.1 mya	Brazil, Candiota Coalfield	Rio Bonito Fm, Seam L2	Seam L2	1	17.0	292.6	290	300
Data from: Glasspool and Scott, 2010	Middle Sakmarian, 295.0 to 290.1 mya	Brazil, Candiota Coalfield	Rio Bonito Fm, Seam S3	Seam S3	1	27.0	292.6	290	300
Data from: Glasspool and Scott, 2010	Middle Sakmarian, 295.0 to 290.1 mya	Brazil, Candiota Coalfield	Rio Bonito Fm, Seam S4	Seam S4	1	47.0	292.6	290	300
Data from: Glasspool and Scott, 2010	Middle Sakmarian, 295.0 to 290.1 mya	Brazil, Candiota Coalfield	Rio Bonito Fm, Seam S5	Seam S5	1	28.0	292.6	290	300

Data from: Glasspool and Scott, 2010	Middle Sakmarian, 295.0 to 290.1 mya	Brazil, Candiota Coalfield	Rio Bonito Fm, Seam S6	Seam S6	1	26.0	292.6	290	300
Data from: Glasspool and Scott, 2010	Middle Sakmarian, 295.0 to 290.1 mya	Brazil, Candiota Coalfield	Rio Bonito Fm, Seam S7	Seam S7	1	26.0	292.6	290	300
Data from: Glasspool and Scott, 2010	Middle Sakmarian, 295.0 to 290.1 mya	Brazil, Candiota Coalfield	Rio Bonito Fm, Seam S8	Seam S8	1	24.0	292.6	290	300
Data from: Glasspool and Scott, 2010	Middle Sakmarian, 295.0 to 290.1 mya	Brazil, Candiota Coalfield	Rio Bonito Fm, Seam S9	Seam S9	1	79.0	292.6	290	300
Data from: Glasspool and Scott, 2010	Middle Sakmarian, 295.0 to 290.1 mya	Brazil, Leão-Butiá Coalfield	Rio Bonito Fm, Camada Inferior		1	33.5	292.6	290	300
Data from: Glasspool and Scott, 2010	Middle Sakmarian, 295.0 to 290.1 mya	Brazil, Leão-Butiá Coalfield	Rio Bonito Fm, Camada Inferior 2		1	46.8	292.6	290	300
Data from: Glasspool and Scott, 2010	Middle Sakmarian, 295.0 to 290.1 mya	Brazil, Leão-Butiá Coalfield	Rio Bonito Fm, Camada Superior 1		1	34.2	292.6	290	300
Data from: Glasspool and Scott, 2010	Middle Sakmarian, 295.0 to 290.1 mya	Brazil, Leão-Butiá Coalfield	Rio Bonito Fm, Camada Superior 2		1	32.5	292.6	290	300
Data from: Glasspool and Scott, 2010	Middle Sakmarian, 295.0 to 290.1 mya	Brazil, Leão-Butiá Coalfield	Rio Bonito Fm, Camada Superior 3		1	34.2	292.6	290	300
Data from: Glasspool and Scott, 2010	Middle Sakmarian, 295.0 to 290.1 mya	Brazil, Leão-Butiá Coalfield	Rio Bonito Fm, Leito 1		1	40.8	292.6	290	300
Data from: Glasspool and Scott, 2010	Middle Sakmarian, 295.0 to 290.1 mya	Brazil, Leão-Butiá Coalfield	Rio Bonito Fm, Leito 2		1	18.0	292.6	290	300
Data from: Glasspool and Scott, 2010	Middle Sakmarian, 295.0 to 290.1 mya	Brazil, Leão-Butiá Coalfield	Rio Bonito Fm, Leito 3		1	33.3	292.6	290	300
Data from: Glasspool and Scott, 2010	Middle Sakmarian, 295.0 to 290.1 mya	Brazil, Leão-Butiá Coalfield	Rio Bonito Fm, Leito 4		1	22.4	292.6	290	300
Data from: Glasspool and Scott, 2010	Middle Sakmarian, 295.0 to 290.1 mya	Brazil, Leão-Butiá Coalfield	Rio Bonito Fm, Seam A1	Seam A1	1	26.0	292.6	290	300
Data from: Glasspool and Scott, 2010	Middle Sakmarian, 295.0 to 290.1 mya	Brazil, Leão-Butiá Coalfield	Rio Bonito Fm, Seam A2	Seam A2	1	10.0	292.6	290	300
Data from: Glasspool and Scott, 2010	Middle Sakmarian, 295.0 to 290.1 mya	Brazil, Leão-Butiá Coalfield	Rio Bonito Fm, Seam B	Seam B	1	10.0	292.6	290	300
Data from: Glasspool and Scott, 2010	Middle Sakmarian, 295.0 to 290.1 mya	Brazil, Leão-Butiá Coalfield	Rio Bonito Fm, Seam C	Seam C	1	9.0	292.6	290	300
Data from: Glasspool and Scott, 2010	Middle Sakmarian, 295.0 to 290.1 mya	Brazil, Leão-Butiá Coalfield	Rio Bonito Fm, Seam CI	Seam CI	1	32.0	292.6	290	300
Data from: Glasspool and Scott, 2010	Middle Sakmarian, 295.0 to 290.1 mya	Brazil, Leão-Butiá Coalfield	Rio Bonito Fm, Seam Leito	Seam Leito	1	32.0	292.6	290	300
Data from: Glasspool and Scott, 2010	Middle Sakmarian, 295.0 to 290.1 mya	Brazil, Leão-Butiá Coalfield	Rio Bonito Fm, Seam M1	Seam M1	1	25.0	292.6	290	300
Data from: Glasspool and Scott, 2010	Middle Sakmarian, 295.0 to 290.1 mya	Brazil, Leão-Butiá Coalfield	Rio Bonito Fm, Seam M2	Seam M2	1	33.0	292.6	290	300
Data from: Glasspool and Scott, 2010	Middle Sakmarian, 295.0 to 290.1 mya	Brazil, Leão-Butiá Coalfield	Rio Bonito Fm, Seam S1	Seam S1	1	24.0	292.6	290	300
Data from: Glasspool and Scott, 2010	Middle Sakmarian, 295.0 to 290.1 mya	Brazil, Leão-Butiá Coalfield	Rio Bonito Fm, Seam S2	Seam S2	1	26.0	292.6	290	300
Data from: Glasspool and Scott, 2010	Middle Sakmarian, 295.0 to 290.1 mya	Brazil, Leão-Butiá Coalfield	Rio Bonito Fm, Seam S3	Seam S3	1	17.0	292.6	290	300
Data from: Glasspool and Scott, 2010	Middle Sakmarian, 295.0 to 290.1 mya	Brazil, Santa Terezinha Coalfield	Rio Bonito Fm, Seam A	Seam A	1	36.0	292.6	290	300

Data from: Glasspool and Scott, 2010	Middle Sakmarian, 295.0 to 290.1 mya	Brazil, Santa Terezinha Coalfield	Rio Bonito Fm, Seam B	Seam B	1	56.0	292.6	290	300
Data from: Glasspool and Scott, 2010	Middle Sakmarian, 295.0 to 290.1 mya	Brazil, Santa Terezinha Coalfield	Rio Bonito Fm, Seam C	Seam C	1	31.0	292.6	290	300
Data from: Glasspool and Scott, 2010	Middle Sakmarian, 295.0 to 290.1 mya	Brazil, Santa Terezinha Coalfield	Rio Bonito Fm, Seam Cl3	Seam Cl3	1	22.0	292.6	290	300
Data from: Glasspool and Scott, 2010	Middle Sakmarian, 295.0 to 290.1 mya	Brazil, Santa Terezinha Coalfield	Rio Bonito Fm, Seam D	Seam D	1	18.0	292.6	290	300
Data from: Glasspool and Scott, 2010	Middle Sakmarian, 295.0 to 290.1 mya	Brazil, Santa Terezinha Coalfield	Rio Bonito Fm, Seam E	Seam E	1	49.0	292.6	290	300
Data from: Glasspool and Scott, 2010	Middle Sakmarian, 295.0 to 290.1 mya	Brazil, Santa Terezinha Coalfield	Rio Bonito Fm, Seam F	Seam F (Av. 3)	3	66.7	292.6	290	300
Data from: Glasspool and Scott, 2010	Middle Sakmarian, 295.0 to 290.1 mya	Brazil, Santa Terezinha Coalfield	Rio Bonito Fm, Seam G	Seam G (Av. 3)	3	39.7	292.6	290	300
Data from: Glasspool and Scott, 2010	Middle Sakmarian, 295.0 to 290.1 mya	Brazil, Santa Terezinha Coalfield	Rio Bonito Fm, Seam I	Seam I	1	30.0	292.6	290	300
Data from: Glasspool and Scott, 2010	Middle Sakmarian, 295.0 to 290.1 mya	Brazil, Santa Terezinha Coalfield	Rio Bonito Fm, Seam Leito	Seam Leito	1	9.0	292.6	290	300
Data from: Glasspool and Scott, 2010	Sakmarian, 295.0 to 290.1 mya	India, Palamau, Bihar, Auranga Coalfield	Lower Kaharbari Fm, Seam I		2	48.7	292.6	290	300
Data from: Glasspool and Scott, 2010	Sakmarian, 295.0 to 290.1 mya	India, Son Valley Coalfield	Kaharbari Fm	Unclear how many seams	4	33.8	292.6	290	300
Data from: Glasspool and Scott, 2010	Sakmarian, 295.0 to 290.1 mya	India, West Bokaro Coalfield	Karharbari Fm, Seam 0		1	70.0	292.6	290	300
Data from: Glasspool and Scott, 2010	Sakmarian, 295.0 to 290.1 mya	Zimbabwe, Wankie Coalfield		Unclear how many seams	4	46.8	292.6	290	300
Smyth, 1972	Asselian-Sakmarian, 298.9 to 290.1	Australia, Brolga No. 1 Well Cooper Basin	Tirrawarra Fm	Table 3.8: 2917.9m	1	50.0	294.5	290	300
Smyth, 1972	Asselian-Sakmarian, 298.9 to 290.1	Australia, Brolga No. 1 Well Cooper Basin	Tirrawarra Fm	Table 3.8: 2925.5-2926.8m	1	71.0	294.5	290	300
Smyth, 1972	Asselian-Sakmarian, 298.9 to 290.1	Australia, Brolga No. 1 Well Cooper Basin	Tirrawarra Fm	Table 3.8: 2932.7m	1	67.0	294.5	290	300
Smyth, 1972	Asselian-Sakmarian, 298.9 to 290.1	Australia, Brolga No. 1 Well Cooper Basin	Tirrawarra Fm	Table 3.8: 2935.2m	1	35.0	294.5	290	300
Smyth, 1972	Asselian-Sakmarian, 298.9 to 290.1	Australia, Fly Lake No. 2 Well Cooper Basin	Tirrawarra Fm	Table 3.6: 2918.0-2918.2m	1	51.0	294.5	290	300
Smyth, 1972	Asselian-Sakmarian, 298.9 to 290.1	Australia, Mudrangie No. 1 Well, Cooper Basin, Patchawarra Trough	Tirrawarra Fm	Table 4.5: 3078.5-3081.5m	1	60.0	294.5	290	300
Smyth, 1972	Asselian-Sakmarian, 298.9 to 290.1	Australia, Tindilpie No. 1 Well, Cooper Basin, Patchawarra Trough	Tirrawarra Fm	Table 4.16: 10580ft	1	60.0	294.5	290	300
Smyth, 1972	Asselian-Sakmarian, 298.9 to 290.1	Australia, Tindilpie No. 1 Well, Cooper Basin, Patchawarra Trough	Tirrawarra Fm	Table 4.16: 10590ft	1	45.0	294.5	290	300
Smyth, 1972	Asselian-Sakmarian, 298.9 to 290.1	Australia, Tindilpie No. 1 Well, Cooper Basin, Patchawarra Trough	Tirrawarra Fm	Table 4.16: 10600ft	1	55.0	294.5	290	300
Smyth, 1972	Asselian-Sakmarian, 298.9 to 290.1	Australia, Tindilpie No. 1 Well, Cooper Basin, Patchawarra Trough	Tirrawarra Fm	Table 4.16: 10630ft	1	62.0	294.5	290	300
Smyth, 1972	Asselian-Sakmarian, 298.9 to 290.1	Australia, Tindilpie No. 1 Well, Cooper Basin, Patchawarra Trough	Tirrawarra Fm	Table 4.16: 10640ft	1	65.0	294.5	290	300
Smyth, 1972	Asselian-Sakmarian, 298.9 to 290.1	Australia, Tindilpie No. 1 Well, Cooper	Tirrawarra Fm	Table 4.16: 10650ft	1	76.0	294.5	290	300

		Basin, Patchawarra Trough							
Smyth, 1972	Asselian-Sakmarian, 298.9 to 290.1	Australia, Tindilpie No. 1 Well, Cooper Basin, Patchawarra Trough	Tirrawarra Fm	Table 4.16: 10700ft	1	64.0	294.5	290	300
Smyth, 1972	Asselian-Sakmarian, 298.9 to 290.1	Australia, Tindilpie No. 1 Well, Cooper Basin, Patchawarra Trough	Tirrawarra Fm	Table 4.16: 10720ft	1	56.0	294.5	290	300
Smyth, 1972	Asselian-Sakmarian, 298.9 to 290.1	Australia, Tindilpie No. 1 Well, Cooper Basin, Patchawarra Trough	Tirrawarra Fm	Table 4.16: 10730ft	1	75.0	294.5	290	300
Smyth, 1972	Asselian-Sakmarian, 298.9 to 290.1	Australia, Tindilpie No. 1 Well, Cooper Basin, Patchawarra Trough	Tirrawarra Fm	Table 4.16: 10740ft	1	54.0	294.5	290	300
Smyth, 1972	Asselian-Sakmarian, 298.9 to 290.1	Australia, Tindilpie No. 1 Well, Cooper Basin, Patchawarra Trough	Tirrawarra Fm	Table 4.16: 10770ft	1	72.0	294.5	290	300
Smyth, 1972	Asselian-Sakmarian, 298.9 to 290.1	Australia, Tindilpie No. 1 Well, Cooper Basin, Patchawarra Trough	Tirrawarra Fm	Table 4.16: 10780ft	1	67.0	294.5	290	300
Smyth, 1972	Asselian-Sakmarian, 298.9 to 290.1	Australia, Tindilpie No. 1 Well, Cooper Basin, Patchawarra Trough	Tirrawarra Fm	Table 4.16: 10820ft	1	53.0	294.5	290	300
Smyth, 1972	Asselian-Sakmarian, 298.9 to 290.1	Australia, Tindilpie No. 1 Well, Cooper Basin, Patchawarra Trough	Tirrawarra Fm	Table 4.16: 10830ft	1	65.0	294.5	290	300
Smyth, 1972	Asselian-Sakmarian, 298.9 to 290.1	Australia, Tindilpie No. 1 Well, Cooper Basin, Patchawarra Trough	Tirrawarra Fm	Table 4.16: 10840ft	1	73.0	294.5	290	300
Data from: Glasspool and Scott, 2010	Gzhelian-Asselian, 303.7 to 295.0 mya	Germany, Dohlen Basin (Rotliegend)			1	5.0	299.4	300	300
Smyth, 1972	Kasimovian-Asselian, 307.0 to 295.0	Australia, Tindilpie No. 1 Well, Cooper Basin, Patchawarra Trough	Merrimelia Fm	Table 4.17: 10890ft	1	72.0	301.0	300	300
Smyth, 1972	Kasimovian-Asselian, 307.0 to 295.0	Australia, Tindilpie No. 1 Well, Cooper Basin, Patchawarra Trough	Merrimelia Fm	Table 4.17: 10900ft	1	57.0	301.0	300	300
Smyth, 1972	Kasimovian-Asselian, 307.0 to 295.0	Australia, Tindilpie No. 1 Well, Cooper Basin, Patchawarra Trough	Merrimelia Fm	Table 4.17: 10930ft	1	71.0	301.0	300	300
Smyth, 1972	Kasimovian-Asselian, 307.0 to 295.0	Australia, Tindilpie No. 1 Well, Cooper Basin, Patchawarra Trough	Merrimelia Fm	Table 4.17: 10940ft	1	89.0	301.0	300	300
Smyth, 1972	Kasimovian-Asselian, 307.0 to 295.0	Australia, Tindilpie No. 1 Well, Cooper Basin, Patchawarra Trough	Merrimelia Fm	Table 4.17: 10950ft	1	60.0	301.0	300	300
Smyth, 1972	Kasimovian-Asselian, 307.0 to 295.0	Australia, Tindilpie No. 1 Well, Cooper Basin, Patchawarra Trough	Merrimelia Fm	Table 4.17: 10960ft	1	28.0	301.0	300	300
Smyth, 1972	Kasimovian-Asselian, 307.0 to 295.0	Australia, Tindilpie No. 1 Well, Cooper Basin, Patchawarra Trough	Merrimelia Fm	Table 4.17: 10990ft	1	75.0	301.0	300	300
Smyth, 1972	Kasimovian-Asselian, 307.0 to 295.0	Australia, Tindilpie No. 1 Well, Cooper Basin, Patchawarra Trough	Merrimelia Fm	Table 4.17: 11000ft	1	86.0	301.0	300	300
Smyth, 1972	Kasimovian-Asselian, 307.0 to 295.0	Australia, Tindilpie No. 1 Well, Cooper Basin, Patchawarra Trough	Merrimelia Fm	Table 4.17: 11010ft	1	88.0	301.0	300	300

Smyth, 1972	Kasimovian-Asselian, 307.0 to 295.0	Australia, Tindilpie No. 1 Well, Cooper Basin, Patchawarra Trough	Merrimelia Fm	Table 4.17: 11020ft	1	44.0	301.0	300	300
Smyth, 1972	Kasimovian-Asselian, 307.0 to 295.0	Australia, Tindilpie No. 1 Well, Cooper Basin, Patchawarra Trough	Merrimelia Fm	Table 4.17: 11030ft	1	82.0	301.0	300	300
Smyth, 1972	Kasimovian-Asselian, 307.0 to 295.0	Australia, Tindilpie No. 1 Well, Cooper Basin, Patchawarra Trough	Merrimelia Fm	Table 4.17: 11050ft	1	54.0	301.0	300	300
Smyth, 1972	Kasimovian-Asselian, 307.0 to 295.0	Australia, Tindilpie No. 1 Well, Cooper Basin, Patchawarra Trough	Merrimelia Fm	Table 4.17: 11060ft	1	70.0	301.0	300	300
Smyth, 1972	Kasimovian-Asselian, 307.0 to 295.0	Australia, Tindilpie No. 1 Well, Cooper Basin, Patchawarra Trough	Merrimelia Fm	Table 4.17: 11080ft	1	71.0	301.0	300	300
Smyth, 1972	Kasimovian-Asselian, 307.0 to 295.0	Australia, Tindilpie No. 1 Well, Cooper Basin, Patchawarra Trough	Merrimelia Fm	Table 4.17: 11090ft	1	57.0	301.0	300	300
Smyth, 1972	Kasimovian-Asselian, 307.0 to 295.0	Australia, Tindilpie No. 1 Well, Cooper Basin, Patchawarra Trough	Merrimelia Fm	Table 4.17: 11100ft	1	67.0	301.0	300	300
Smyth, 1972	Kasimovian-Asselian, 307.0 to 295.0	Australia, Tindilpie No. 1 Well, Cooper Basin, Patchawarra Trough	Merrimelia Fm	Table 4.17: 11110ft	1	70.0	301.0	300	300
Smyth, 1972	Kasimovian-Asselian, 307.0 to 295.0	Australia, Tindilpie No. 1 Well, Cooper Basin, Patchawarra Trough	Merrimelia Fm	Table 4.17: 11130ft	1	62.0	301.0	300	300
Smyth, 1972	Kasimovian-Asselian, 307.0 to 295.0	Australia, Tindilpie No. 1 Well, Cooper Basin, Patchawarra Trough	Merrimelia Fm	Table 4.17: 11140ft	1	77.0	301.0	300	300
Smyth, 1972	Kasimovian-Asselian, 307.0 to 295.0	Australia, Tindilpie No. 1 Well, Cooper Basin, Patchawarra Trough	Merrimelia Fm	Table 4.17: 11160ft	1	78.0	301.0	300	300
Data from: Glasspool and Scott, 2010	Late Pennsylvanian, 307.0 to 298.9 mya	France, Massif Central, Ales Basin	Samples A20-26 (one seam)		7	8.3	303.0	300	300
Data from: Glasspool and Scott, 2010	Late Pennsylvanian, 307.0 to 298.9 mya	France, Massif Central, Ales Basin	Samples A30-36 (one seam)		7	8.3	303.0	300	300
Data from: Glasspool and Scott, 2010	Late Pennsylvanian, 307.0 to 298.9 mya	Germany, Saar Coalfield	Stephanian A Coals		5	14.8	303.0	300	300
Rodrigues et al., 2011	Late Pennsylvanian, 307.0 to 298.9 mya	Portugal, Douro Basin		Unclear how many seams	1	41.4	303.0	300	300
Data from: Glasspool and Scott, 2010	Late Pennsylvanian, 307.0 to 298.9 mya	Ukraine, Donets Basin	N2/2 MC599		1	29.0	303.0	300	300
Data from: Glasspool and Scott, 2010	Late Pennsylvanian, 307.0 to 298.9 mya	Ukraine, Donets Basin	n3/2 MC599		1	16.0	303.0	300	300
Data from: Glasspool and Scott, 2010	Late Pennsylvanian, 307.0 to 298.9 mya	Ukraine, Donets Basin	n6/3 MC599		1	4.0	303.0	300	300
Data from: Glasspool and Scott, 2010	Late Pennsylvanian, 307.0 to 298.9 mya	Ukraine, Donets Basin	o2 Svet		1	7.0	303.0	300	300
Data from: Glasspool and Scott, 2010	Late Pennsylvanian, 307.0 to 298.9 mya	USA, Illinois	Bristol Hill		1	16.6	303.0	300	300
Data from: Glasspool and Scott, 2010	Late Pennsylvanian, 307.0 to 298.9 mya	USA, Illinois	Flannigan		1	29.4	303.0	300	300
Data from: Glasspool and Scott, 2010	Late Pennsylvanian, 307.0 to 298.9 mya	USA, Illinois	New Haven		2	19.7	303.0	300	300

Data from: Glasspool and Scott, 2010	Late Pennsylvanian, 307.0 to 298.9 mya	USA, Illinois	Shelbyville		1	22.4	303.0	300	300
Data from: Glasspool and Scott, 2010	Late Pennsylvanian, 307.0 to 298.9 mya	USA, Illinois	Trowbridge		1	16.4	303.0	300	300
Data from: Glasspool and Scott, 2010	Late Pennsylvanian, 307.0 to 298.9 mya	USA, Illinois	Womac		1	19.9	303.0	300	300
Data from: Glasspool and Scott, 2010	Late Pennsylvanian, 307.0 to 298.9 mya	USA, Illinois, Eastern Coalfield	Calhoun		2	7.1	303.0	300	300
Data from: Glasspool and Scott, 2010	Late Pennsylvanian, 307.0 to 298.9 mya	USA, Illinois, Eastern Coalfield	Mattoon Formation, Opdyke		4	11.3	303.0	300	300
Data from: Glasspool and Scott, 2010	Late Pennsylvanian, 307.0 to 298.9 mya	USA, Maryland, Appalachian Coalfield	Glenshaw Fm., Conemaugh Gp., Brush Creek / Twin		10	11.6	303.0	300	300
Data from: Glasspool and Scott, 2010	Late Pennsylvanian, 307.0 to 298.9 mya	USA, Ohio, Appalachian Coalfield	Monongahela Gp., Meigs Creek / Ohio #9		2	28.1	303.0	300	300
Data from: Glasspool and Scott, 2010	Late Pennsylvanian, 307.0 to 298.9 mya	USA, Ohio, Appalachian Coalfield	Monongahela Gp., Pomeroy / Ohio #8A		1	8.7	303.0	300	300
Data from: Glasspool and Scott, 2010	Late Pennsylvanian, 307.0 to 298.9 mya	USA, Ohio, Appalachian Coalfield	Monongahela Gp., Waynesburg / Ohio #11		9	12.9	303.0	300	300
Data from: Glasspool and Scott, 2010	Late Pennsylvanian, 307.0 to 298.9 mya	USA, Ohio, Appalachian Coalfield	Washington Fm, Dunkard Gp., Ohio #12A		1	10.1	303.0	300	300
Data from: Glasspool and Scott, 2010	Late Pennsylvanian, 307.0 to 298.9 mya	USA, Ohio, Appalachian Coalfield	Washington Fm, Dunkard Gp., Washington / Ohio #12		1	30.6	303.0	300	300
Data from: Glasspool and Scott, 2010	Late Pennsylvanian, 307.0 to 298.9 mya	USA, Pennsylvania	Lower Freeport		1	21.4	303.0	300	300
Data from: Glasspool and Scott, 2010	Late Pennsylvanian, 307.0 to 298.9 mya	USA, Pennsylvania, Appalachian Coalfield	Monongahela Gp., Sewickley		2	8.8	303.0	300	300
Data from: Glasspool and Scott, 2010	Late Pennsylvanian, 307.0 to 298.9 mya	USA, Pennsylvania, Ohio, West Virginia, Appalachian Coalfield	Monongahela Gp., Pittsburgh / Ohio #8		52	12.3	303.0	300	300
Data from: Glasspool and Scott, 2010	Late Pennsylvanian, 307.0 to 298.9 mya	USA, Pennsylvania, West Virginia, Appalachian Coalfield	Monongahela Fm, Redstone		13	6.5	303.0	300	300
Data from: Glasspool and Scott, 2010	Late Pennsylvanian, 307.0 to 298.9 mya	USA, West Virginia, Appalachian Coalfield	Conemaugh Fm., Elk Lick		6	7.1	303.0	300	300
Data from: Glasspool and Scott, 2010	Middle Pennsylvanian, 315.2 to 307 mya	Canada, Nova Scotia	15ft Seam, M-26		1	14.0	311.1	310	315
Data from: Glasspool and Scott, 2010	Middle Pennsylvanian, 315.2 to 307 mya	Canada, Nova Scotia	3 Ft Seam, M-19		1	14.0	311.1	310	315
Data from: Glasspool and Scott, 2010	Middle Pennsylvanian, 315.2 to 307 mya	Canada, Nova Scotia	3ft 10 inch seam, M-11		1	5.0	311.1	310	315
Data from: Glasspool and Scott, 2010	Middle Pennsylvanian, 315.2 to 307 mya	Canada, Nova Scotia	3ft 6 inch Seam, M-17		1	15.0	311.1	310	315
Data from: Glasspool and Scott, 2010	Middle Pennsylvanian, 315.2 to 307 mya	Canada, Nova Scotia	4ft 8inch seam, M-1		1	16.0	311.1	310	315
Data from: Glasspool and Scott, 2010	Middle Pennsylvanian, 315.2 to 307 mya	Canada, Nova Scotia	5 ft 3inch Seam, M-12		1	10.0	311.1	310	315
Data from: Glasspool and Scott, 2010	Middle Pennsylvanian, 315.2 to 307 mya	Canada, Nova Scotia	5 ft Seam, M-2		1	11.0	311.1	310	315
Data from: Glasspool and Scott, 2010	Middle Pennsylvanian, 315.2 to 307 mya	Canada, Nova Scotia	7 ft Seam, M-7		1	14.0	311.1	310	315

Data from: Glasspool and Scott, 2010	Middle Pennsylvanian, 315.2 to 307 mya	Canada, Nova Scotia	8 Ft seam, M-9Upp.		1	10.0	311.1	310	315
Data from: Glasspool and Scott, 2010	Middle Pennsylvanian, 315.2 to 307 mya	Canada, Nova Scotia	Foord Seam	(Table 1)	20	28.0	311.1	310	315
Data from: Glasspool and Scott, 2010	Middle Pennsylvanian, 315.2 to 307 mya	Canada, Nova Scotia	New 11 ft Seam, M-27/28		1	8.0	311.1	310	315
Data from: Glasspool and Scott, 2010	Middle Pennsylvanian, 315.2 to 307 mya	Canada, Nova Scotia, Sydney Coalfield	Harbour Seam	(Table 2 composite)	1	13.0	311.1	310	315
Data from: Glasspool and Scott, 2010	Middle Pennsylvanian, 315.2 to 307 mya	Canada, Nova Scotia, Sydney Coalfield	Hub Seam	(Table 2 composite)	1	11.0	311.1	310	315
Data from: Glasspool and Scott, 2010	Middle Pennsylvanian, 315.2 to 307 mya	Germany, Ibbenburen Coalfield	Westphalian C, Seam 13/14		1	17.0	311.1	310	315
Data from: Glasspool and Scott, 2010	Middle Pennsylvanian, 315.2 to 307 mya	Germany, Ibbenburen Coalfield	Westphalian C, Seam 15		1	69.0	311.1	310	315
Data from: Glasspool and Scott, 2010	Middle Pennsylvanian, 315.2 to 307 mya	Germany, Ibbenburen Coalfield	Westphalian C, Seam 17		1	31.0	311.1	310	315
Data from: Glasspool and Scott, 2010	Middle Pennsylvanian, 315.2 to 307 mya	Germany, Ibbenburen Coalfield	Westphalian C, Seam 19		1	28.0	311.1	310	315
Data from: Glasspool and Scott, 2010	Middle Pennsylvanian, 315.2 to 307 mya	Germany, Ibbenburen Coalfield	Westphalian C, Seam 2		1	22.0	311.1	310	315
Data from: Glasspool and Scott, 2010	Middle Pennsylvanian, 315.2 to 307 mya	Germany, Ibbenburen Coalfield	Westphalian C, Seam 21		1	24.0	311.1	310	315
Data from: Glasspool and Scott, 2010	Middle Pennsylvanian, 315.2 to 307 mya	Germany, Ibbenburen Coalfield	Westphalian C, Seam Bentingsbank		1	27.0	311.1	310	315
Data from: Glasspool and Scott, 2010	Middle Pennsylvanian, 315.2 to 307 mya	Germany, Ibbenburen Coalfield	Westphalian C, Seam Gltícksburg		1	24.0	311.1	310	315
Data from: Glasspool and Scott, 2010	Middle Pennsylvanian, 315.2 to 307 mya	Germany, Ibbenburen Coalfield	Westphalian C, Seam Reden		1	26.0	311.1	310	315
Data from: Glasspool and Scott, 2010	Middle Pennsylvanian, 315.2 to 307 mya	Germany, Ibbenburen Coalfield	Westphalian C, Seam Saar		1	23.0	311.1	310	315
Data from: Glasspool and Scott, 2010	Middle Pennsylvanian, 315.2 to 307 mya	Germany, Ibbenburen Coalfield	Westphalian D, Seam Flottwell		1	24.0	311.1	310	315
Data from: Glasspool and Scott, 2010	Middle Pennsylvanian, 315.2 to 307 mya	Germany, Ruhr Basin		Unclear how many seams	13	13.5	311.1	310	315
Data from: Glasspool and Scott, 2010	Middle Pennsylvanian, 315.2 to 307 mya	Germany, Ruhr Basin	Seam Baldur		2	31.0	311.1	310	315
Data from: Glasspool and Scott, 2010	Middle Pennsylvanian, 315.2 to 307 mya	Germany, Ruhr Basin	Seam Hagen 1-3		2	21.3	311.1	310	315
Data from: Glasspool and Scott, 2010	Middle Pennsylvanian, 315.2 to 307 mya	Germany, Ruhr Basin	Seam Hagen10bk		2	23.3	311.1	310	315
Data from: Glasspool and Scott, 2010	Middle Pennsylvanian, 315.2 to 307 mya	Germany, Ruhr Basin	Seam Iduna		1	12.7	311.1	310	315
Data from: Glasspool and Scott, 2010	Middle Pennsylvanian, 315.2 to 307 mya	Germany, Ruhr Basin	Seam Loki Mbk		1	10.8	311.1	310	315
Data from: Glasspool and Scott, 2010	Middle Pennsylvanian, 315.2 to 307 mya	Germany, Ruhr Basin	Seam Loki Obk		1	16.2	311.1	310	315
Data from: Glasspool and Scott, 2010	Middle Pennsylvanian, 315.2 to 307 mya	Germany, Ruhr Basin	Seam Midgard 2		1	9.7	311.1	310	315
Data from: Glasspool and Scott, 2010	Middle Pennsylvanian, 315.2 to 307 mya	Germany, Ruhr Basin	Westphalian C, Chriemhilt 2/3, Seam 27b		1	11.0	311.1	310	315

Data from: Glasspool and Scott, 2010	Middle Pennsylvanian, 315.2 to 307 mya	Germany, Ruhr Basin	Westphalian C, Hagen l/2, Seam 20		1	16.0	311.1	310	315
Data from: Glasspool and Scott, 2010	Middle Pennsylvanian, 315.2 to 307 mya	Germany, Ruhr Basin	Westphalian C, Iduna Group, Seam 15c		1	18.0	311.1	310	315
Data from: Glasspool and Scott, 2010	Middle Pennsylvanian, 315.2 to 307 mya	Germany, Ruhr Basin	Westphalian C, Loki, Seam 11		1	6.0	311.1	310	315
Data from: Glasspool and Scott, 2010	Middle Pennsylvanian, 315.2 to 307 mya	Germany, Ruhr Basin	Westphalian C, Midgard 2, Seam 10		1	6.0	311.1	310	315
Data from: Glasspool and Scott, 2010	Middle Pennsylvanian, 315.2 to 307 mya	Germany, Ruhr Basin	Westphalian C, Nibelung Group, Seam No. 3		1	17.0	311.1	310	315
Data from: Glasspool and Scott, 2010	Middle Pennsylvanian, 315.2 to 307 mya	Germany, Ruhr Basin	Westphalian C, Nibelung Group, Seam No. 7		1	9.0	311.1	310	315
Data from: Glasspool and Scott, 2010	Middle Pennsylvanian, 315.2 to 307 mya	Germany, Ruhr Coalfield	Westphalian C, Seam Hagen1		2	18.0	311.1	310	315
Data from: Glasspool and Scott, 2010	Middle Pennsylvanian, 315.2 to 307 mya	Germany, Ruhr Coalfield	Westphalian C, Seam Hagen1Ubk		1	20.0	311.1	310	315
Data from: Glasspool and Scott, 2010	Middle Pennsylvanian, 315.2 to 307 mya	Germany, Ruhr Coalfield	Westphalian C, Seam Hagen1Ubk/2/3		3	20.7	311.1	310	315
Data from: Glasspool and Scott, 2010	Middle Pennsylvanian, 315.2 to 307 mya	Germany, Ruhr Coalfield	Westphalian C, Seam Hagen2/3		1	26.0	311.1	310	315
Data from: Glasspool and Scott, 2010	Middle Pennsylvanian, 315.2 to 307 mya	Germany, Ruhr Coalfield	Westphalian C, Seam Hagen3		2	24.0	311.1	310	315
Data from: Glasspool and Scott, 2010	Middle Pennsylvanian, 315.2 to 307 mya	Germany, Ruhr Coalfield	Westphalian C, Seam Hagen4		5	25.6	311.1	310	315
Data from: Glasspool and Scott, 2010	Middle Pennsylvanian, 315.2 to 307 mya	Germany, Ruhr Coalfield	Westphalian C, Seam Nibelung		3	21.3	311.1	310	315
Data from: Glasspool and Scott, 2010	Middle Pennsylvanian, 315.2 to 307 mya	Germany, Ruhr Coalfield	Westphalian C, Seam Odin		4	19.8	311.1	310	315
Data from: Glasspool and Scott, 2010	Middle Pennsylvanian, 315.2 to 307 mya	Germany, Ruhr Coalfield	Westphalian C, Seam Parsifal		1	15.0	311.1	310	315
Data from: Glasspool and Scott, 2010	Middle Pennsylvanian, 315.2 to 307 mya	Germany, Ruhr Coalfield	Westphalian C, Seam Riibezahl		1	27.0	311.1	310	315
Data from: Glasspool and Scott, 2010	Middle Pennsylvanian, 315.2 to 307 mya	Germany, Ruhr Coalfield	Westphalian C, Seam Siegfried		2	25.0	311.1	310	315
Data from: Glasspool and Scott, 2010	Middle Pennsylvanian, 315.2 to 307 mya	Germany, Ruhr Coalfield	Westphalian C, Seam Volker		2	15.5	311.1	310	315
Data from: Glasspool and Scott, 2010	Middle Pennsylvanian, 315.2 to 307 mya	Germany, Saar Coalfield	Westphalian D coals		8	12.2	311.1	310	315
Data from: Glasspool and Scott, 2010	Middle Pennsylvanian, 315.2 to 307 mya	Netherlands, Limbricht 1/1a Well	Seam IX Lower		1	8.2	311.1	310	315
Data from: Glasspool and Scott, 2010	Middle Pennsylvanian, 315.2 to 307 mya	Netherlands, Limbricht 1/1a Well	Seam IX Upper		1	8.8	311.1	310	315
Data from: Glasspool and Scott, 2010	Middle Pennsylvanian, 315.2 to 307 mya	Netherlands, Limbricht 1/1a Well	Seam VIII Lower		1	16.8	311.1	310	315
Data from: Glasspool and Scott, 2010	Middle Pennsylvanian, 315.2 to 307 mya	Netherlands, Limbricht 1/1a Well	Seam VIII Upper		1	13.3	311.1	310	315
Data from: Glasspool and Scott, 2010	Middle Pennsylvanian, 315.2 to 307 mya	Netherlands, Limbricht 1/1a Well	Seam X		1	18.9	311.1	310	315
Data from: Glasspool and Scott, 2010	Middle Pennsylvanian, 315.2 to 307 mya	Netherlands, Limbricht 1/1a Well	Seam XI		1	12.6	311.1	310	315

Data from: Glasspool and Scott, 2010	Middle Pennsylvanian, 315.2 to 307 mya	Netherlands, Limbricht 1/1a Well	Seam XXIV		1	8.9	311.1	310	315
Data from: Glasspool and Scott, 2010	Middle Pennsylvanian, 315.2 to 307 mya	Netherlands, Limbricht 1/1a Well	Seam XXV		1	17.5	311.1	310	315
Data from: Glasspool and Scott, 2010	Middle Pennsylvanian, 315.2 to 307 mya	Poland	Laziska Beds, Coal 207		1	36.0	311.1	310	315
Brownfield et al., 2001	Middle Pennsylvanian, 315.2 to 307 mya	Russia, Rostovskaya, Donetsky, Central`naya Vosto K 2			1	12.0	311.1	310	315
Brownfield et al., 2001	Middle Pennsylvanian, 315.2 to 307 mya	Russia, Rostovskaya, Donetsky, Gukovskya K 6			1	11.0	311.1	310	315
Brownfield et al., 2001	Middle Pennsylvanian, 315.2 to 307 mya	Russia, Rostovskaya, Donetsky, Uglerod K 5			1	9.0	311.1	310	315
Brownfield et al., 2001	Middle Pennsylvanian, 315.2 to 307 mya	Russia, Rostovskaya, Zverevsky, Donetsky, Gukovo-Zverevskoye, Obukhovskaya K2			1	11.0	311.1	310	315
Brownfield et al., 2001	Middle Pennsylvanian, 315.2 to 307 mya	Russia, Rostovskaya, Zverevsky, Donetsky, Gukovo-Zverevskoye, Zamchalovskaya K2H			1	11.4	311.1	310	315
Data from: Glasspool and Scott, 2010	Middle Pennsylvanian, 315.2 to 307 mya	Spain	Canales Pack		3	17.3	311.1	310	315
Data from: Glasspool and Scott, 2010	Middle Pennsylvanian, 315.2 to 307 mya	Spain	Entrerregueras Pack		3	8.6	311.1	310	315
Data from: Glasspool and Scott, 2010	Middle Pennsylvanian, 315.2 to 307 mya	Spain	Generalas Pack		5	15.5	311.1	310	315
Data from: Glasspool and Scott, 2010	Middle Pennsylvanian, 315.2 to 307 mya	Spain	Maria Luisa Pack		10	12.3	311.1	310	315
Data from: Glasspool and Scott, 2010	Middle Pennsylvanian, 315.2 to 307 mya	Spain	Pudingas Pack		1	22.8	311.1	310	315
Data from: Glasspool and Scott, 2010	Middle Pennsylvanian, 315.2 to 307 mya	Spain	Soton Pack		8	13.2	311.1	310	315
Rodrigues et al., 2011	Middle Pennsylvanian, 315.2 to 307 mya	Spain, Peñarroya-Belmez-Espiel Basin		Unclear how many seams	1	10.0	311.1	310	315
Data from: Glasspool and Scott, 2010	Middle Pennsylvanian, 315.2 to 307 mya	Turkey, Zonguldak Basin	Kalin seam		1	17.7	311.1	310	315
Data from: Glasspool and Scott, 2010	Middle Pennsylvanian, 315.2 to 307 mya	Turkey, Zonguldak Basin	Kurudere seam		1	19.4	311.1	310	315
Data from: Glasspool and Scott, 2010	Middle Pennsylvanian, 315.2 to 307 mya	Turkey, Zonguldak Basin	Tasli seam		1	18.9	311.1	310	315
Data from: Glasspool and Scott, 2010	Middle Pennsylvanian, 315.2 to 307 mya	Turkey, Zonguldak Basin	Tavan seam		1	22.3	311.1	310	315
Data from: Glasspool and Scott, 2010	Middle Pennsylvanian, 315.2 to 307 mya	Ukraine, Donets Basin	10l3 Alm		1	18.0	311.1	310	315
Data from: Glasspool and Scott, 2010	Middle Pennsylvanian, 315.2 to 307 mya	Ukraine, Donets Basin	10l3 Bel		1	20.0	311.1	310	315
Data from: Glasspool and Scott, 2010	Middle Pennsylvanian, 315.2 to 307 mya	Ukraine, Donets Basin	10m3 Baz		1	0.0	311.1	310	315
Data from: Glasspool and Scott, 2010	Middle Pennsylvanian, 315.2 to 307 mya	Ukraine, Donets Basin	11l3 Alm		1	14.0	311.1	310	315

Data from: Glasspool and Scott, 2010	Middle Pennsylvanian, 315.2 to 307 mya	Ukraine, Donets Basin	12l3 Bel		1	12.0	311.1	310	315
Data from: Glasspool and Scott, 2010	Middle Pennsylvanian, 315.2 to 307 mya	Ukraine, Donets Basin	1k2/2 Ka		1	58.0	311.1	310	315
Data from: Glasspool and Scott, 2010	Middle Pennsylvanian, 315.2 to 307 mya	Ukraine, Donets Basin	1k7 Cen		1	6.0	311.1	310	315
Data from: Glasspool and Scott, 2010	Middle Pennsylvanian, 315.2 to 307 mya	Ukraine, Donets Basin	1k7 Dim		1	10.0	311.1	310	315
Data from: Glasspool and Scott, 2010	Middle Pennsylvanian, 315.2 to 307 mya	Ukraine, Donets Basin	1K8 Dob		1	12.0	311.1	310	315
Data from: Glasspool and Scott, 2010	Middle Pennsylvanian, 315.2 to 307 mya	Ukraine, Donets Basin	1k8 Nov		1	14.0	311.1	310	315
Data from: Glasspool and Scott, 2010	Middle Pennsylvanian, 315.2 to 307 mya	Ukraine, Donets Basin	1l1 13-bis		1	16.0	311.1	310	315
Data from: Glasspool and Scott, 2010	Middle Pennsylvanian, 315.2 to 307 mya	Ukraine, Donets Basin	1l1 Dim		1	15.0	311.1	310	315
Data from: Glasspool and Scott, 2010	Middle Pennsylvanian, 315.2 to 307 mya	Ukraine, Donets Basin	1l1 Dob		1	8.0	311.1	310	315
Data from: Glasspool and Scott, 2010	Middle Pennsylvanian, 315.2 to 307 mya	Ukraine, Donets Basin	1l1 Nov		1	25.0	311.1	310	315
Data from: Glasspool and Scott, 2010	Middle Pennsylvanian, 315.2 to 307 mya	Ukraine, Donets Basin	1l3 Bel		1	6.0	311.1	310	315
Data from: Glasspool and Scott, 2010	Middle Pennsylvanian, 315.2 to 307 mya	Ukraine, Donets Basin	1l8 Bel		1	5.0	311.1	310	315
Data from: Glasspool and Scott, 2010	Middle Pennsylvanian, 315.2 to 307 mya	Ukraine, Donets Basin	1m2 Bel		1	6.0	311.1	310	315
Data from: Glasspool and Scott, 2010	Middle Pennsylvanian, 315.2 to 307 mya	Ukraine, Donets Basin	1m3 Baz		1	0.0	311.1	310	315
Data from: Glasspool and Scott, 2010	Middle Pennsylvanian, 315.2 to 307 mya	Ukraine, Donets Basin	1m9 MC 598		1	10.0	311.1	310	315
Data from: Glasspool and Scott, 2010	Middle Pennsylvanian, 315.2 to 307 mya	Ukraine, Donets Basin	1n1 But		1	10.0	311.1	310	315
Data from: Glasspool and Scott, 2010	Middle Pennsylvanian, 315.2 to 307 mya	Ukraine, Donets Basin	2k2/2 Ka		1	12.0	311.1	310	315
Data from: Glasspool and Scott, 2010	Middle Pennsylvanian, 315.2 to 307 mya	Ukraine, Donets Basin	2k7 Cen		1	9.0	311.1	310	315
Data from: Glasspool and Scott, 2010	Middle Pennsylvanian, 315.2 to 307 mya	Ukraine, Donets Basin	2l1 13-bis		1	12.0	311.1	310	315
Data from: Glasspool and Scott, 2010	Middle Pennsylvanian, 315.2 to 307 mya	Ukraine, Donets Basin	2l1 Dim		1	8.0	311.1	310	315
Data from: Glasspool and Scott, 2010	Middle Pennsylvanian, 315.2 to 307 mya	Ukraine, Donets Basin	2l1 Nov		1	11.0	311.1	310	315
Data from: Glasspool and Scott, 2010	Middle Pennsylvanian, 315.2 to 307 mya	Ukraine, Donets Basin	2l3 Bel		1	5.0	311.1	310	315
Data from: Glasspool and Scott, 2010	Middle Pennsylvanian, 315.2 to 307 mya	Ukraine, Donets Basin	2m1/5 Alm		1	14.0	311.1	310	315
Data from: Glasspool and Scott, 2010	Middle Pennsylvanian, 315.2 to 307 mya	Ukraine, Donets Basin	2m2 Bel		1	6.0	311.1	310	315
Data from: Glasspool and Scott, 2010	Middle Pennsylvanian, 315.2 to 307 mya	Ukraine, Donets Basin	2m3 Baz		1	22.0	311.1	310	315

Data from: Glasspool and Scott, 2010	Middle Pennsylvanian, 315.2 to 307 mya	Ukraine, Donets Basin	2m9 MC 598		1	15.0	311.1	310	315
Data from: Glasspool and Scott, 2010	Middle Pennsylvanian, 315.2 to 307 mya	Ukraine, Donets Basin	2n1 But		1	12.0	311.1	310	315
Data from: Glasspool and Scott, 2010	Middle Pennsylvanian, 315.2 to 307 mya	Ukraine, Donets Basin	3k5 Krasno		1	9.0	311.1	310	315
Data from: Glasspool and Scott, 2010	Middle Pennsylvanian, 315.2 to 307 mya	Ukraine, Donets Basin	3k7 Cen		1	2.0	311.1	310	315
Data from: Glasspool and Scott, 2010	Middle Pennsylvanian, 315.2 to 307 mya	Ukraine, Donets Basin	3l1 Dim		1	6.0	311.1	310	315
Data from: Glasspool and Scott, 2010	Middle Pennsylvanian, 315.2 to 307 mya	Ukraine, Donets Basin	3l1 Nov		1	3.0	311.1	310	315
Data from: Glasspool and Scott, 2010	Middle Pennsylvanian, 315.2 to 307 mya	Ukraine, Donets Basin	3l3 Bel		1	5.0	311.1	310	315
Data from: Glasspool and Scott, 2010	Middle Pennsylvanian, 315.2 to 307 mya	Ukraine, Donets Basin	3m1/5 Alm		1	19.0	311.1	310	315
Data from: Glasspool and Scott, 2010	Middle Pennsylvanian, 315.2 to 307 mya	Ukraine, Donets Basin	3m2 Bel		1	15.0	311.1	310	315
Data from: Glasspool and Scott, 2010	Middle Pennsylvanian, 315.2 to 307 mya	Ukraine, Donets Basin	3m3 Baz		1	20.0	311.1	310	315
Data from: Glasspool and Scott, 2010	Middle Pennsylvanian, 315.2 to 307 mya	Ukraine, Donets Basin	3n1 But		1	16.0	311.1	310	315
Data from: Glasspool and Scott, 2010	Middle Pennsylvanian, 315.2 to 307 mya	Ukraine, Donets Basin	4k7 Cen		1	4.0	311.1	310	315
Data from: Glasspool and Scott, 2010	Middle Pennsylvanian, 315.2 to 307 mya	Ukraine, Donets Basin	4l1 13-bis		1	21.0	311.1	310	315
Data from: Glasspool and Scott, 2010	Middle Pennsylvanian, 315.2 to 307 mya	Ukraine, Donets Basin	4l1 Dim		1	18.0	311.1	310	315
Data from: Glasspool and Scott, 2010	Middle Pennsylvanian, 315.2 to 307 mya	Ukraine, Donets Basin	4l1 Nov		1	11.0	311.1	310	315
Data from: Glasspool and Scott, 2010	Middle Pennsylvanian, 315.2 to 307 mya	Ukraine, Donets Basin	4l3 Bel		1	6.0	311.1	310	315
Data from: Glasspool and Scott, 2010	Middle Pennsylvanian, 315.2 to 307 mya	Ukraine, Donets Basin	4m1/5 Alm		1	6.0	311.1	310	315
Data from: Glasspool and Scott, 2010	Middle Pennsylvanian, 315.2 to 307 mya	Ukraine, Donets Basin	4m2Bel		1	0.0	311.1	310	315
Data from: Glasspool and Scott, 2010	Middle Pennsylvanian, 315.2 to 307 mya	Ukraine, Donets Basin	4m3 Baz		1	16.0	311.1	310	315
Data from: Glasspool and Scott, 2010	Middle Pennsylvanian, 315.2 to 307 mya	Ukraine, Donets Basin	4m4 Dob		1	11.0	311.1	310	315
Data from: Glasspool and Scott, 2010	Middle Pennsylvanian, 315.2 to 307 mya	Ukraine, Donets Basin	4n1 But		1	32.0	311.1	310	315
Data from: Glasspool and Scott, 2010	Middle Pennsylvanian, 315.2 to 307 mya	Ukraine, Donets Basin	5k7 Cen		1	7.0	311.1	310	315
Data from: Glasspool and Scott, 2010	Middle Pennsylvanian, 315.2 to 307 mya	Ukraine, Donets Basin	5l1 13-bis		1	16.0	311.1	310	315
Data from: Glasspool and Scott, 2010	Middle Pennsylvanian, 315.2 to 307 mya	Ukraine, Donets Basin	5l1 Dim		1	18.0	311.1	310	315
Data from: Glasspool and Scott, 2010	Middle Pennsylvanian, 315.2 to 307 mya	Ukraine, Donets Basin	5l1 Nov		1	1.0	311.1	310	315

Data from: Glasspool and Scott, 2010	Middle Pennsylvanian, 315.2 to 307 mya	Ukraine, Donets Basin	5l3 Bel		1	13.0	311.1	310	315
Data from: Glasspool and Scott, 2010	Middle Pennsylvanian, 315.2 to 307 mya	Ukraine, Donets Basin	5m1/5 Alm		1	17.0	311.1	310	315
Data from: Glasspool and Scott, 2010	Middle Pennsylvanian, 315.2 to 307 mya	Ukraine, Donets Basin	5m2 Bel		1	10.0	311.1	310	315
Data from: Glasspool and Scott, 2010	Middle Pennsylvanian, 315.2 to 307 mya	Ukraine, Donets Basin	5m3 Baz		1	15.0	311.1	310	315
Data from: Glasspool and Scott, 2010	Middle Pennsylvanian, 315.2 to 307 mya	Ukraine, Donets Basin	5n1 But		1	15.0	311.1	310	315
Data from: Glasspool and Scott, 2010	Middle Pennsylvanian, 315.2 to 307 mya	Ukraine, Donets Basin	6l1 13-bis		1	13.0	311.1	310	315
Data from: Glasspool and Scott, 2010	Middle Pennsylvanian, 315.2 to 307 mya	Ukraine, Donets Basin	6l1 Dim		1	15.0	311.1	310	315
Data from: Glasspool and Scott, 2010	Middle Pennsylvanian, 315.2 to 307 mya	Ukraine, Donets Basin	6l1 Nov		1	8.0	311.1	310	315
Data from: Glasspool and Scott, 2010	Middle Pennsylvanian, 315.2 to 307 mya	Ukraine, Donets Basin	6l3 Alm		1	7.0	311.1	310	315
Data from: Glasspool and Scott, 2010	Middle Pennsylvanian, 315.2 to 307 mya	Ukraine, Donets Basin	6l3Bel		1	16.0	311.1	310	315
Data from: Glasspool and Scott, 2010	Middle Pennsylvanian, 315.2 to 307 mya	Ukraine, Donets Basin	6m2 Bel		1	11.0	311.1	310	315
Data from: Glasspool and Scott, 2010	Middle Pennsylvanian, 315.2 to 307 mya	Ukraine, Donets Basin	6m3 Baz		1	9.0	311.1	310	315
Data from: Glasspool and Scott, 2010	Middle Pennsylvanian, 315.2 to 307 mya	Ukraine, Donets Basin	7l1 13-bis		1	0.0	311.1	310	315
Data from: Glasspool and Scott, 2010	Middle Pennsylvanian, 315.2 to 307 mya	Ukraine, Donets Basin	7l1 Dim		1	0.0	311.1	310	315
Data from: Glasspool and Scott, 2010	Middle Pennsylvanian, 315.2 to 307 mya	Ukraine, Donets Basin	7l3 Alm		1	5.0	311.1	310	315
Data from: Glasspool and Scott, 2010	Middle Pennsylvanian, 315.2 to 307 mya	Ukraine, Donets Basin	7l3 Bel		1	12.0	311.1	310	315
Data from: Glasspool and Scott, 2010	Middle Pennsylvanian, 315.2 to 307 mya	Ukraine, Donets Basin	7m2 Bel		1	11.0	311.1	310	315
Data from: Glasspool and Scott, 2010	Middle Pennsylvanian, 315.2 to 307 mya	Ukraine, Donets Basin	7m3 Baz		1	20.0	311.1	310	315
Data from: Glasspool and Scott, 2010	Middle Pennsylvanian, 315.2 to 307 mya	Ukraine, Donets Basin	8l1 Dim		1	22.0	311.1	310	315
Data from: Glasspool and Scott, 2010	Middle Pennsylvanian, 315.2 to 307 mya	Ukraine, Donets Basin	8l3 Alm		1	6.0	311.1	310	315
Data from: Glasspool and Scott, 2010	Middle Pennsylvanian, 315.2 to 307 mya	Ukraine, Donets Basin	8l3 Bel		1	11.0	311.1	310	315
Data from: Glasspool and Scott, 2010	Middle Pennsylvanian, 315.2 to 307 mya	Ukraine, Donets Basin	8m3 Baz		1	11.0	311.1	310	315
Data from: Glasspool and Scott, 2010	Middle Pennsylvanian, 315.2 to 307 mya	Ukraine, Donets Basin	9l1 Dim		1	24.0	311.1	310	315
Data from: Glasspool and Scott, 2010	Middle Pennsylvanian, 315.2 to 307 mya	Ukraine, Donets Basin	9l3 Alm		1	13.0	311.1	310	315
Data from: Glasspool and Scott, 2010	Middle Pennsylvanian, 315.2 to 307 mya	Ukraine, Donets Basin	9l3 Bel		1	24.0	311.1	310	315

Data from: Glasspool and Scott, 2010	Middle Pennsylvanian, 315.2 to 307 mya	Ukraine, Donets Basin	k2u M 1859		1	16.0	311.1	310	315
Data from: Glasspool and Scott, 2010	Middle Pennsylvanian, 315.2 to 307 mya	Ukraine, Donets Basin	l4 Trudo		1	10.0	311.1	310	315
Data from: Glasspool and Scott, 2010	Middle Pennsylvanian, 315.2 to 307 mya	Ukraine, Donets Basin	m1/8 Sch 1355		1	12.0	311.1	310	315
Data from: Glasspool and Scott, 2010	Middle Pennsylvanian, 315.2 to 307 mya	Ukraine, Donets Basin	m3 Trudo		1	4.0	311.1	310	315
Data from: Glasspool and Scott, 2010	Middle Pennsylvanian, 315.2 to 307 mya	Ukraine, Donets Basin	m3 Za		1	25.0	311.1	310	315
Data from: Glasspool and Scott, 2010	Middle Pennsylvanian, 315.2 to 307 mya	Ukraine, Donets Basin	m3/6 Sch 1355		1	13.0	311.1	310	315
Data from: Glasspool and Scott, 2010	Middle Pennsylvanian, 315.2 to 307 mya	Ukraine, Donets Basin	m7 MC 598		1	7.0	311.1	310	315
Brownfield et al., 2001	Middle Pennsylvanian, 315.2 to 307 mya	Ukraine, Donetskaya, Donetsky, Tsentral`ny, Karla Marksa Derezovka			1	10.0	311.1	310	315
Brownfield et al., 2001	Middle Pennsylvanian, 315.2 to 307 mya	Ukraine, Donetskaya, Donetsky, Tzentral`ny, Ol`khovatskaya Derezovka			1	7.0	311.1	310	315
Data from: Glasspool and Scott, 2010	Middle Pennsylvanian, 315.2 to 307 mya	United Kingdom, England, Cumbria, Keekle	Sample 10-E48388, Depth 32m		1	13.5	311.1	310	315
Data from: Glasspool and Scott, 2010	Middle Pennsylvanian, 315.2 to 307 mya	United Kingdom, England, Cumbria, Keekle	Sample 11-E48389, Depth 40m		1	5.5	311.1	310	315
Data from: Glasspool and Scott, 2010	Middle Pennsylvanian, 315.2 to 307 mya	United Kingdom, England, Cumbria, Keekle	Sample 12-E48390, Depth 64m		1	10.5	311.1	310	315
Data from: Glasspool and Scott, 2010	Middle Pennsylvanian, 315.2 to 307 mya	USA	Block Coal (Lower Member)		1	14.9	311.1	310	315
Data from: Glasspool and Scott, 2010	Middle Pennsylvanian, 315.2 to 307 mya	USA, Arkansas, Oklahoma, Western Coalfield	Hartshorne Sandstone, Hartshorne (Lower Hartshorne)		5	16.1	311.1	310	315
Data from: Glasspool and Scott, 2010	Middle Pennsylvanian, 315.2 to 307 mya	USA, Arkansas, Western Coalfield	Philpott		6	13.1	311.1	310	315
Data from: Glasspool and Scott, 2010	Middle Pennsylvanian, 315.2 to 307 mya	USA, Arkansas, Western Coalfield	Philpott - (Lower Philpott)		4	19.0	311.1	310	315
Data from: Glasspool and Scott, 2010	Middle Pennsylvanian, 315.2 to 307 mya	USA, Arkansas, Western Coalfield	Savanna Fm, Paris		6	10.1	311.1	310	315
Data from: Glasspool and Scott, 2010	Middle Pennsylvanian, 315.2 to 307 mya	USA, Arkansas, Western Coalfield	Spadra - (Lower Spadra)		1	12.3	311.1	310	315
Data from: Glasspool and Scott, 2010	Middle Pennsylvanian, 315.2 to 307 mya	USA, Arkansas, Western Coalfield	Spadra - (Upper Spadra)		1	12.3	311.1	310	315
Data from: Glasspool and Scott, 2010	Middle Pennsylvanian, 315.2 to 307 mya	USA, Illinois	Athensville		1	12.3	311.1	310	315
Data from: Glasspool and Scott, 2010	Middle Pennsylvanian, 315.2 to 307 mya	USA, Illinois	Briar Hill		3	8.3	311.1	310	315
Data from: Glasspool and Scott, 2010	Middle Pennsylvanian, 315.2 to 307 mya	USA, Illinois	Houchin Creek		4	8.4	311.1	310	315
Data from: Glasspool and Scott, 2010	Middle Pennsylvanian, 315.2 to 307 mya	USA, Illinois	Jamestown		1	18.4	311.1	310	315
Data from: Glasspool and Scott, 2010	Middle Pennsylvanian, 315.2 to 307 mya	USA, Illinois	Murphysboro		2	7.1	311.1	310	315

Data from: Glasspool and Scott, 2010	Middle Pennsylvanian, 315.2 to 307 mya	USA, Illinois	New Burnside		1	9.0	311.1	310	315
Data from: Glasspool and Scott, 2010	Middle Pennsylvanian, 315.2 to 307 mya	USA, Illinois	Reynoldsburg		2	6.4	311.1	310	315
Data from: Glasspool and Scott, 2010	Middle Pennsylvanian, 315.2 to 307 mya	USA, Illinois	Rock Branch		1	4.8	311.1	310	315
Data from: Glasspool and Scott, 2010	Middle Pennsylvanian, 315.2 to 307 mya	USA, Illinois	Seelyville		1	11.9	311.1	310	315
Data from: Glasspool and Scott, 2010	Middle Pennsylvanian, 315.2 to 307 mya	USA, Illinois	Survant		1	15.6	311.1	310	315
Data from: Glasspool and Scott, 2010	Middle Pennsylvanian, 315.2 to 307 mya	USA, Illinois, Eastern Coalfield	Carbondale Fm, Dekoven - (Lower Dekoven)		1	10.1	311.1	310	315
Data from: Glasspool and Scott, 2010	Middle Pennsylvanian, 315.2 to 307 mya	USA, Illinois, Eastern Coalfield	Carbondale Fm, Dekoven - (Top Dekoven)		1	24.6	311.1	310	315
Data from: Glasspool and Scott, 2010	Middle Pennsylvanian, 315.2 to 307 mya	USA, Illinois, Eastern Coalfield	Carbondale Fm, Summum / Houchin Creek / Illinois #4		2	15.2	311.1	310	315
Data from: Glasspool and Scott, 2010	Middle Pennsylvanian, 315.2 to 307 mya	USA, Illinois, Eastern Coalfield	Tradewater Fm, Illinois #1 (Rock Island)		2	13.5	311.1	310	315
Data from: Glasspool and Scott, 2010	Middle Pennsylvanian, 315.2 to 307 mya	USA, Illinois, Indiana, Eastern Coalfield	Carbondale Fm, Colchester / Illinois #2 / Indiana #3		20	13.1	311.1	310	315
Data from: Glasspool and Scott, 2010	Middle Pennsylvanian, 315.2 to 307 mya	USA, Illinois, Indiana, Kentucky, Eastern Coalfield	Carbondale Fm, Herrin / Illinois #6 / Indiana # 6 / Kentucky #11		86	18.0	311.1	310	315
Data from: Glasspool and Scott, 2010	Middle Pennsylvanian, 315.2 to 307 mya	USA, Illinois, Indiana, Kentucky, Eastern Coalfield	Carbondale Fm, Springfield / Illinois #5 / Indiana #5 /Kentucky #9		30	11.0	311.1	310	315
Data from: Glasspool and Scott, 2010	Middle Pennsylvanian, 315.2 to 307 mya	USA, Illinois, Kentucky, Eastern Coalfield	Carbondale Fm, Davis/Dekoven/Kentucky #6		11	15.5	311.1	310	315
Data from: Glasspool and Scott, 2010	Middle Pennsylvanian, 315.2 to 307 mya	USA, Illlinois, Indiana, Eastern Coalfield	Shelburn Fm, Danville / Illinois #7 / Indiana #7		16	8.8	311.1	310	315
Data from: Glasspool and Scott, 2010	Middle Pennsylvanian, 315.2 to 307 mya	USA, Indiana, Eastern Coalfield	Brazil Fm, Block - (Brazil Block Upper and Lower)		14	23.3	311.1	310	315
Data from: Glasspool and Scott, 2010	Middle Pennsylvanian, 315.2 to 307 mya	USA, Kansas, Oklahoma, Western Coalfield	Cabaniss Fm., Cherokee Gp., Mineral		4	8.9	311.1	310	315
Data from: Glasspool and Scott, 2010	Middle Pennsylvanian, 315.2 to 307 mya	USA, Kentucky	Breathitt Fm, Skyline Lower Split		1	34.4	311.1	310	315
Data from: Glasspool and Scott, 2010	Middle Pennsylvanian, 315.2 to 307 mya	USA, Kentucky	Leatherwood		1	25.3	311.1	310	315
Data from: Glasspool and Scott, 2010	Middle Pennsylvanian, 315.2 to 307 mya	USA, Kentucky	Lower Broas-Stockton Seam		1	26.8	311.1	310	315
Data from: Glasspool and Scott, 2010	Middle Pennsylvanian, 315.2 to 307 mya	USA, Kentucky	Stockton		2	21.9	311.1	310	315
Data from: Glasspool and Scott, 2010	Middle Pennsylvanian, 315.2 to 307 mya	USA, Kentucky, Appalachian Coalfield	Breathitt Fm, Hazard #5A		4	22.5	311.1	310	315
Data from: Glasspool and Scott, 2010	Middle Pennsylvanian, 315.2 to 307 mya	USA, Kentucky, Appalachian Coalfield	Breathitt Fm, Hazard #7		4	20.3	311.1	310	315
Data from: Glasspool and Scott, 2010	Middle Pennsylvanian, 315.2 to 307 mya	USA, Kentucky, Appalachian Coalfield	Breathitt Fm, Hazard #9		4	13.6	311.1	310	315
Data from: Glasspool and Scott, 2010	Middle Pennsylvanian, 315.2 to 307 mya	USA, Kentucky, Eastern Coalfield	Mining City / No. 4 Coal		1	13.7	311.1	310	315

Data from: Glasspool and Scott, 2010	Middle Pennsylvanian, 315.2 to 307 mya	USA, Kentucky, Eastern Coalfield	Sturgis Fm, Paradise / Kentucky #12		12	7.6	311.1	310	315
Data from: Glasspool and Scott, 2010	Middle Pennsylvanian, 315.2 to 307 mya	USA, Kentucky, Eastern Coalfield	Tradewater Fm, Coiltown / Kentucky #14		1	10.9	311.1	310	315
Data from: Glasspool and Scott, 2010	Middle Pennsylvanian, 315.2 to 307 mya	USA, Kentucky, Eastern Coalfield	Tradewater Fm, Mannington / Kentucky #4		2	12.2	311.1	310	315
Data from: Glasspool and Scott, 2010	Middle Pennsylvanian, 315.2 to 307 mya	USA, Montana, Krebs Fm, Krebs Gp	Western, Rowe		2	13.9	311.1	310	315
Data from: Glasspool and Scott, 2010	Middle Pennsylvanian, 315.2 to 307 mya	USA, Montana, Western Coalfield	Appanoose Gp, Mulberry		3	10.9	311.1	310	315
Data from: Glasspool and Scott, 2010	Middle Pennsylvanian, 315.2 to 307 mya	USA, Montana, Western Coalfield	Cabaniss Gp, Bevier-Wheeler		4	15.3	311.1	310	315
Data from: Glasspool and Scott, 2010	Middle Pennsylvanian, 315.2 to 307 mya	USA, Montana, Western Coalfield	Cabaniss Gp, Tebo		4	14.7	311.1	310	315
Data from: Glasspool and Scott, 2010	Middle Pennsylvanian, 315.2 to 307 mya	USA, Montana, Western Coalfield	Cabaniss Gp, Weir-Pittsburg		1	13.4	311.1	310	315
Data from: Glasspool and Scott, 2010	Middle Pennsylvanian, 315.2 to 307 mya	USA, Montana, Western Coalfield	Knifeton		1	13.5	311.1	310	315
Data from: Glasspool and Scott, 2010	Middle Pennsylvanian, 315.2 to 307 mya	USA, Montana, Western Coalfield	Krebs Fm, Krebs Gp, Drywood		1	9.5	311.1	310	315
Data from: Glasspool and Scott, 2010	Middle Pennsylvanian, 315.2 to 307 mya	USA, Montana, Western Coalfield	Rich Hill - (Upper Rich Hill)		1	16.9	311.1	310	315
Data from: Glasspool and Scott, 2010	Middle Pennsylvanian, 315.2 to 307 mya	USA, Montana, Western Coalfield	Summit		1	11.7	311.1	310	315
Data from: Glasspool and Scott, 2010	Middle Pennsylvanian, 315.2 to 307 mya	USA, Montana, Western Coalfield	Wheeler		1	6.8	311.1	310	315
Data from: Glasspool and Scott, 2010	Middle Pennsylvanian, 315.2 to 307 mya	USA, Ohio, Appalachian Coalfield	Pottsville Gp, Mercer - (Lower Mercer) / Ohio #3		1	14.6	311.1	310	315
Data from: Glasspool and Scott, 2010	Middle Pennsylvanian, 315.2 to 307 mya	USA, Ohio, Appalachian Coalfield	Pottsville Gp, Quakertown / Ohio #2		1	17.2	311.1	310	315
Data from: Glasspool and Scott, 2010	Middle Pennsylvanian, 315.2 to 307 mya	USA, Oklahoma, Western Coalfield	Cabaniss Fm., Croweburg		12	4.9	311.1	310	315
Data from: Glasspool and Scott, 2010	Middle Pennsylvanian, 315.2 to 307 mya	USA, Oklahoma, Western Coalfield	Hartshorne Sandstone, Hartshorne		3	13.2	311.1	310	315
Data from: Glasspool and Scott, 2010	Middle Pennsylvanian, 315.2 to 307 mya	USA, Oklahoma, Western Coalfield	Hartshorne Sandstone, Hartshorne - (Upper Hartshorne)		4	10.0	311.1	310	315
Data from: Glasspool and Scott, 2010	Middle Pennsylvanian, 315.2 to 307 mya	USA, Oklahoma, Western Coalfield	Hartshorne Sandstone, Hartshorne Rider		1	15.3	311.1	310	315
Data from: Glasspool and Scott, 2010	Middle Pennsylvanian, 315.2 to 307 mya	USA, Oklahoma, Western Coalfield	McAlester Fm, Stigler		6	12.0	311.1	310	315
Data from: Glasspool and Scott, 2010	Middle Pennsylvanian, 315.2 to 307 mya	USA, Pennsylvania	Allegheny Fm, Kittaning Fm, P&M seam B		6	17.9	311.1	310	315
Data from: Glasspool and Scott, 2010	Middle Pennsylvanian, 315.2 to 307 mya	USA, Pennsylvania	Llewellyn Formation, Mammoth		1	10.5	311.1	310	315
Data from: Glasspool and Scott, 2010	Middle Pennsylvanian, 315.2 to 307 mya	USA, Pennsylvania	Llewellyn Formation, Parlor		1	12.9	311.1	310	315
Data from: Glasspool and Scott, 2010	Middle Pennsylvanian, 315.2 to 307 mya	USA, Pennsylvania	Mercer		1	11.7	311.1	310	315
Data from: Glasspool and Scott, 2010	Middle Pennsylvanian, 315.2 to 307 mya	USA, Pennsylvania, Anthracite Coalfield	Allegheny Fm, Kittaning Fm,		1	9.5	311.1	310	315

			Penna. Semianthracite C							
Data from: Glasspool and Scott, 2010	Middle Pennsylvanian, 315.2 to 307 mya	USA, Pennsylvania, Anthracite Coalfield	Llewellyn Formation, Pennsylvania #2		4	9.1	311.1	310	315
Data from: Glasspool and Scott, 2010	Middle Pennsylvanian, 315.2 to 307 mya	USA, Pennsylvania, Anthracite Coalfield	Llewellyn Formation, Pennsylvania #8 - (All splits)		3	17.1	311.1	310	315
Data from: Glasspool and Scott, 2010	Middle Pennsylvanian, 315.2 to 307 mya	USA, Pennsylvania, Anthracite Coalfield	Llewellyn Formation, Primrose		4	18.1	311.1	310	315
Data from: Glasspool and Scott, 2010	Middle Pennsylvanian, 315.2 to 307 mya	USA, Pennsylvania, Anthracite Coalfield	Llewellyn Formation, Skidmore - (Lower Skidmore) / Ross		1	7.7	311.1	310	315
Data from: Glasspool and Scott, 2010	Middle Pennsylvanian, 315.2 to 307 mya	USA, Pennsylvania, Anthracite Coalfield	Llewellyn Formation, Skidmore / Wharton		1	4.3	311.1	310	315
Data from: Glasspool and Scott, 2010	Middle Pennsylvanian, 315.2 to 307 mya	USA, Pennsylvania, Appalachian Coalfield	Allegheny Fm, Clarion - (Upper Clarion / Scrubrass		2	13.9	311.1	310	315
Data from: Glasspool and Scott, 2010	Middle Pennsylvanian, 315.2 to 307 mya	USA, Pennsylvania, Appalachian Coalfield	Allegheny Gp, Bear Creek		3	28.2	311.1	310	315
Data from: Glasspool and Scott, 2010	Middle Pennsylvanian, 315.2 to 307 mya	USA, Pennsylvania, Appalachian Coalfield	Clarion Fm., Allegheny Gp, Clarion - (Lower Clarion)		5	10.4	311.1	310	315
Data from: Glasspool and Scott, 2010	Middle Pennsylvanian, 315.2 to 307 mya	USA, Pennsylvania, Appalachian Coalfield	Clarion Fm., Allegheny Gp, Fulton		1	15.6	311.1	310	315
Data from: Glasspool and Scott, 2010	Middle Pennsylvanian, 315.2 to 307 mya	USA, Pennsylvania, Appalachian Coalfield	Kittanning Fm., Allegheny Gp, Barnett		1	7.3	311.1	310	315
Data from: Glasspool and Scott, 2010	Middle Pennsylvanian, 315.2 to 307 mya	USA, Pennsylvania, Appalachian Coalfield	Kittanning Fm., Allegheny Gp, Bloss		5	10.0	311.1	310	315
Data from: Glasspool and Scott, 2010	Middle Pennsylvanian, 315.2 to 307 mya	USA, Pennsylvania, Appalachian Coalfield	Llewellyn Formation, Buck Mountain		5	14.6	311.1	310	315
Data from: Glasspool and Scott, 2010	Middle Pennsylvanian, 315.2 to 307 mya	USA, Pennsylvania, Ohio, Appalachian Coalfield	Clarion Fm., Allegheny Gp, Brookville / Ohio #4		10	19.6	311.1	310	315
Data from: Glasspool and Scott, 2010	Middle Pennsylvanian, 315.2 to 307 mya	USA, Pennsylvania, Ohio, Appalachian Coalfield	Clarion Fm., Allegheny Gp, Clarion / Ohio #4A		9	17.3	311.1	310	315
Data from: Glasspool and Scott, 2010	Middle Pennsylvanian, 315.2 to 307 mya	USA, Pennsylvania, Ohio, Appalachian Coalfield	Freeport Fm., Allegheny Gp, Freeport - (Lower Freeport) / Ohio #6A		10	23.3	311.1	310	315
Data from: Glasspool and Scott, 2010	Middle Pennsylvanian, 315.2 to 307 mya	USA, Pennsylvania, Ohio, West Virginia, Appalachian Coalfield	Kittanning Fm., Allegheny Gp, Kittanning - (Lower Kittanning) / Ohio #5		204	12.1	311.1	310	315
Data from: Glasspool and Scott, 2010	Middle Pennsylvanian, 315.2 to 307 mya	USA, Pennsylvania, Ohio, West Virginia, Appalachian Coalfield	Kittanning Fm., Allegheny Gp, Kittanning - (Middle Kittanning) / Ohio #6		22	14.3	311.1	310	315
Data from: Glasspool and Scott, 2010	Middle Pennsylvanian, 315.2 to 307 mya	USA, Pennsylvania, West Virginia, Appalachian Coalfield	Allegheny Fm, Kittanning - (Upper Kittanning)		28	24.1	311.1	310	315
Data from: Glasspool and Scott, 2010	Middle Pennsylvanian, 315.2 to 307 mya	USA, Pennsylvania,Kentucky, Ohio, Appalachian Coalfield	Allegheny Fm, Freeport - (Upper Freeport) / Ohio #7)		13	10.5	311.1	310	315
Data from: Glasspool and Scott, 2010	Middle Pennsylvanian, 315.2 to 307 mya	USA, Tennessee, Appalachian Coalfield	Pee Wee		2	16.1	311.1	310	315

Data from: Glasspool and Scott, 2010	Middle Pennsylvanian, 315.2 to 307 mya	USA, West Virginia, Appalachian Coalfield	Allegheny Fm, West Virginia #5 Block		1	27.3	311.1	310	315
Data from: Glasspool and Scott, 2010	Middle Pennsylvanian, 315.2 to 307 mya	USA, West Virginia, Appalachian Coalfield	Kanawha Fm, Stockton-Lewiston		7	26.1	311.1	310	315
Data from: Glasspool and Scott, 2010	Early Pennsylvanian, 323.2 to 315.2 mya	Canada, Nova Scotia, Cape Breton, Chimney Corner	No. 1		1	17.0	319.2	320	315
Data from: Glasspool and Scott, 2010	Early Pennsylvanian, 323.2 to 315.2 mya	Canada, Nova Scotia, Cape Breton, Chimney Corner	No. 1A		1	20.0	319.2	320	315
Data from: Glasspool and Scott, 2010	Early Pennsylvanian, 323.2 to 315.2 mya	Canada, Nova Scotia, Cape Breton, Chimney Corner	No. 2		1	24.0	319.2	320	315
Data from: Glasspool and Scott, 2010	Early Pennsylvanian, 323.2 to 315.2 mya	Canada, Nova Scotia, Cape Breton, Chimney Corner	No. 3		1	7.0	319.2	320	315
Data from: Glasspool and Scott, 2010	Early Pennsylvanian, 323.2 to 315.2 mya	Canada, Nova Scotia, Cape Breton, Chimney Corner	No. 4		1	20.0	319.2	320	315
Data from: Glasspool and Scott, 2010	Early Pennsylvanian, 323.2 to 315.2 mya	Canada, Nova Scotia, Cape Breton, Chimney Corner	No. 5		3	22.3	319.2	320	315
Data from: Glasspool and Scott, 2010	Early Pennsylvanian, 323.2 to 315.2 mya	Canada, Nova Scotia, Cape Breton, St. Roses	No. 5		5	31.8	319.2	320	315
Data from: Glasspool and Scott, 2010	Early Pennsylvanian, 323.2 to 315.2 mya	Canada, Nova Scotia, Joggins	Clam Coal, Coal 19		1	4.2	319.2	320	315
Data from: Glasspool and Scott, 2010	Early Pennsylvanian, 323.2 to 315.2 mya	Canada, Nova Scotia, Joggins	Fundy Coal, Coal 28		4	6.8	319.2	320	315
Data from: Glasspool and Scott, 2010	Early Pennsylvanian, 323.2 to 315.2 mya	Canada, Nova Scotia, Joggins	Kimberly Lower Split Coal 16		1	14.2	319.2	320	315
Data from: Glasspool and Scott, 2010	Early Pennsylvanian, 323.2 to 315.2 mya	Canada, Nova Scotia, Joggins	Kimberly Middle Split Coal 16		3	10.9	319.2	320	315
Data from: Glasspool and Scott, 2010	Early Pennsylvanian, 323.2 to 315.2 mya	Czech republic, Ostrava-Karvin Coalfield	Doubrava s.s.		1	22.1	319.2	320	315
Data from: Glasspool and Scott, 2010	Early Pennsylvanian, 323.2 to 315.2 mya	Czech republic, Ostrava-Karvin Coalfield	Doubrava Upper		1	18.5	319.2	320	315
Data from: Glasspool and Scott, 2010	Early Pennsylvanian, 323.2 to 315.2 mya	Czech republic, Ostrava-Karvin Coalfield	Saddle M		1	42.6	319.2	320	315
Data from: Glasspool and Scott, 2010	Early Pennsylvanian, 323.2 to 315.2 mya	Czech republic, Ostrava-Karvin Coalfield	Sucha Lower		1	30.1	319.2	320	315
Data from: Glasspool and Scott, 2010	Early Pennsylvanian, 323.2 to 315.2 mya	Czech republic, Ostrava-Karvin Coalfield	Sucha M Upper		1	26.8	319.2	320	315
Data from: Glasspool and Scott, 2010	Early Pennsylvanian, 323.2 to 315.2 mya	Germany, Ibbenburen Coalfield	Westphalian B, Seam 34		1	28.0	319.2	320	315
Data from: Glasspool and Scott, 2010	Early Pennsylvanian, 323.2 to 315.2 mya	Germany, Ibbenburen Coalfield	Westphalian B, Seam 35		1	27.0	319.2	320	315
Data from: Glasspool and Scott, 2010	Early Pennsylvanian, 323.2 to 315.2 mya	Germany, Ibbenburen Coalfield	Westphalian B, Seam 41		1	38.0	319.2	320	315
Data from: Glasspool and Scott, 2010	Early Pennsylvanian, 323.2 to 315.2 mya	Germany, Ruhr		Unclear how many seams	3	19.8	319.2	320	315
Data from: Glasspool and Scott, 2010	Early Pennsylvanian, 323.2 to 315.2 mya	Germany, Ruhr Basin	Seam A		1	57.5	319.2	320	315
Data from: Glasspool and Scott, 2010	Early Pennsylvanian, 323.2 to 315.2 mya	Germany, Ruhr Basin	Seam C1		1	12.8	319.2	320	315
Data from: Glasspool and Scott, 2010	Early Pennsylvanian, 323.2 to 315.2 mya	Germany, Ruhr Basin	Seam C2		1	21.2	319.2	320	315

Data from: Glasspool and Scott, 2010	Early Pennsylvanian, 323.2 to 315.2 mya	Germany, Ruhr Basin	Seam H2/G1		1	26.4	319.2	320	315
Data from: Glasspool and Scott, 2010	Early Pennsylvanian, 323.2 to 315.2 mya	Germany, Ruhr Basin	Seam K2/J1		1	19.5	319.2	320	315
Data from: Glasspool and Scott, 2010	Early Pennsylvanian, 323.2 to 315.2 mya	Germany, Ruhr Basin	Seam N		1	16.8	319.2	320	315
Data from: Glasspool and Scott, 2010	Early Pennsylvanian, 323.2 to 315.2 mya	Germany, Ruhr Basin	Seam O		1	19.8	319.2	320	315
Data from: Glasspool and Scott, 2010	Early Pennsylvanian, 323.2 to 315.2 mya	Germany, Ruhr Basin	Seam P 4 0bk		1	14.5	319.2	320	315
Data from: Glasspool and Scott, 2010	Early Pennsylvanian, 323.2 to 315.2 mya	Germany, Ruhr Basin	Seam P 4 Ubk		1	24.6	319.2	320	315
Data from: Glasspool and Scott, 2010	Early Pennsylvanian, 323.2 to 315.2 mya	Germany, Ruhr Basin	Seam X1		1	14.5	319.2	320	315
Data from: Glasspool and Scott, 2010	Early Pennsylvanian, 323.2 to 315.2 mya	Germany, Ruhr Basin	Seam X2		1	13.7	319.2	320	315
Data from: Glasspool and Scott, 2010	Early Pennsylvanian, 323.2 to 315.2 mya	Germany, Ruhr Basin	Seam Zollv. 3		1	28.3	319.2	320	315
Data from: Glasspool and Scott, 2010	Early Pennsylvanian, 323.2 to 315.2 mya	Germany, Ruhr Basin	Westphalian A, Anna, Seam 4		1	14.0	319.2	320	315
Data from: Glasspool and Scott, 2010	Early Pennsylvanian, 323.2 to 315.2 mya	Germany, Ruhr Basin	Westphalian A, Boghead-Cannel (bone cole)		1	26.0	319.2	320	315
Data from: Glasspool and Scott, 2010	Early Pennsylvanian, 323.2 to 315.2 mya	Germany, Ruhr Basin	Westphalian A, Dickebank, Seam 44		1	20.0	319.2	320	315
Data from: Glasspool and Scott, 2010	Early Pennsylvanian, 323.2 to 315.2 mya	Germany, Ruhr Basin	Westphalian A, Ernestine 2, Seam 33		1	12.0	319.2	320	315
Data from: Glasspool and Scott, 2010	Early Pennsylvanian, 323.2 to 315.2 mya	Germany, Ruhr Basin	Westphalian A, Girondelle 5, Seam 51a		1	14.0	319.2	320	315
Data from: Glasspool and Scott, 2010	Early Pennsylvanian, 323.2 to 315.2 mya	Germany, Ruhr Basin	Westphalian A, Hugo 1, Seam 12		1	18.0	319.2	320	315
Data from: Glasspool and Scott, 2010	Early Pennsylvanian, 323.2 to 315.2 mya	Germany, Ruhr Basin	Westphalian A, Jacob 2/Pras/Helene, Seam 37		1	56.0	319.2	320	315
Data from: Glasspool and Scott, 2010	Early Pennsylvanian, 323.2 to 315.2 mya	Germany, Ruhr Basin	Westphalian A, Karl 2, Seam 25		1	43.0	319.2	320	315
Data from: Glasspool and Scott, 2010	Early Pennsylvanian, 323.2 to 315.2 mya	Germany, Ruhr Basin	Westphalian A, Matthias 1 (bone coal), Seam 6b		1	5.0	319.2	320	315
Data from: Glasspool and Scott, 2010	Early Pennsylvanian, 323.2 to 315.2 mya	Germany, Ruhr Basin	Westphalian A, Matthias 2, Seam 7		1	32.0	319.2	320	315
Data from: Glasspool and Scott, 2010	Early Pennsylvanian, 323.2 to 315.2 mya	Germany, Ruhr Basin	Westphalian A, Wasserfall/Sonnenschein, Seam 47		1	33.0	319.2	320	315
Data from: Glasspool and Scott, 2010	Early Pennsylvanian, 323.2 to 315.2 mya	Germany, Ruhr Basin	Westphalian B, C Seam 68		1	30.0	319.2	320	315
Data from: Glasspool and Scott, 2010	Early Pennsylvanian, 323.2 to 315.2 mya	Germany, Ruhr Basin	Westphalian B, K2, Seam 58		1	18.0	319.2	320	315
Data from: Glasspool and Scott, 2010	Early Pennsylvanian, 323.2 to 315.2 mya	Germany, Ruhr Basin	Westphalian B, L/K1, Seam 57		1	6.0	319.2	320	315
Data from: Glasspool and Scott, 2010	Early Pennsylvanian, 323.2 to 315.2 mya	Germany, Ruhr Basin	Westphalian B, N, Seam 56		1	9.0	319.2	320	315
Data from: Glasspool and Scott, 2010	Early Pennsylvanian, 323.2 to 315.2 mya	Germany, Ruhr Basin	Westphalian B, Q1 Ubk/2, Seam 46		1	11.0	319.2	320	315

Data from: Glasspool and Scott, 2010	Early Pennsylvanian, 323.2 to 315.2 mya	Germany, Ruhr Basin	Westphalian B, S Group, Seam 39b		1	12.0	319.2	320	315
Data from: Glasspool and Scott, 2010	Early Pennsylvanian, 323.2 to 315.2 mya	Germany, Ruhr Basin	Westphalian B, Z, Seam 30		1	4.0	319.2	320	315
Data from: Glasspool and Scott, 2010	Early Pennsylvanian, 323.2 to 315.2 mya	Poland	Coal 504	(Table 4)	1	37.0	319.2	320	315
Data from: Glasspool and Scott, 2010	Early Pennsylvanian, 323.2 to 315.2 mya	Poland	Lower Silesian Coal Basin, Pi-1 Piast 415/2		1	36.6	319.2	320	315
Data from: Glasspool and Scott, 2010	Early Pennsylvanian, 323.2 to 315.2 mya	Poland	Lower Silesian Coal Basin, Supiec 301/2		2	34.6	319.2	320	315
Data from: Glasspool and Scott, 2010	Early Pennsylvanian, 323.2 to 315.2 mya	Poland	Lower Silesian Coal Basin, Supiec 412		1	43.2	319.2	320	315
Data from: Glasspool and Scott, 2010	Early Pennsylvanian, 323.2 to 315.2 mya	Poland	Lower Silesian Coal Basin, Victoria 430		1	26.5	319.2	320	315
Data from: Glasspool and Scott, 2010	Early Pennsylvanian, 323.2 to 315.2 mya	Poland	Lower Silesian Coal Basin, Wabrzych 430		1	37.8	319.2	320	315
Data from: Glasspool and Scott, 2010	Early Pennsylvanian, 323.2 to 315.2 mya	Poland	Lower Silesian Coal Basin, Wabrzych 431/2		1	23.5	319.2	320	315
Data from: Glasspool and Scott, 2010	Early Pennsylvanian, 323.2 to 315.2 mya	Poland	Lublin Coal Basin, Bogdanka-382		2	36.6	319.2	320	315
Data from: Glasspool and Scott, 2010	Early Pennsylvanian, 323.2 to 315.2 mya	Poland	Upper Silesian Coal Basin, B-5 Brzeszcze 510		1	28.3	319.2	320	315
Data from: Glasspool and Scott, 2010	Early Pennsylvanian, 323.2 to 315.2 mya	Poland	Upper Silesian Coal Basin, B-5c Brzeszcze 510		1	42.9	319.2	320	315
Data from: Glasspool and Scott, 2010	Early Pennsylvanian, 323.2 to 315.2 mya	Poland	Upper Silesian Coal Basin, B-7 Brzeszcze 356		1	36.7	319.2	320	315
Data from: Glasspool and Scott, 2010	Early Pennsylvanian, 323.2 to 315.2 mya	Poland	Upper Silesian Coal Basin, B-7c Brzeszcze 356		1	44.9	319.2	320	315
Data from: Glasspool and Scott, 2010	Early Pennsylvanian, 323.2 to 315.2 mya	Poland	Upper Silesian Coal Basin, Brzeszcze-356		1	44.8	319.2	320	315
Data from: Glasspool and Scott, 2010	Early Pennsylvanian, 323.2 to 315.2 mya	Poland	Upper Silesian Coal Basin, Brzeszcze-510		1	42.9	319.2	320	315
Data from: Glasspool and Scott, 2010	Early Pennsylvanian, 323.2 to 315.2 mya	Poland	Upper Silesian Coal Basin, H-1 Halemba 506		1	40.3	319.2	320	315
Data from: Glasspool and Scott, 2010	Early Pennsylvanian, 323.2 to 315.2 mya	Poland	Upper Silesian Coal Basin, Jastrzebie 502/1		3	41.3	319.2	320	315
Data from: Glasspool and Scott, 2010	Early Pennsylvanian, 323.2 to 315.2 mya	Poland	Upper Silesian Coal Basin, Krupinski 348		2	31.4	319.2	320	315
Data from: Glasspool and Scott, 2010	Early Pennsylvanian, 323.2 to 315.2 mya	Poland	Upper Silesian Coal Basin, Miechowice 509		2	4.0	319.2	320	315
Data from: Glasspool and Scott, 2010	Early Pennsylvanian, 323.2 to 315.2 mya	Poland	Upper Silesian Coal Basin, Morcinek 404/2		4	20.5	319.2	320	315
Data from: Glasspool and Scott, 2010	Early Pennsylvanian, 323.2 to 315.2 mya	Poland	Upper Silesian Coal Basin, Morcinek 406/2		2	24.1	319.2	320	315
Data from: Glasspool and Scott, 2010	Early Pennsylvanian, 323.2 to 315.2 mya	Poland	Upper Silesian Coal Basin, Moszczenica 506/3		1	30.1	319.2	320	315
Data from: Glasspool and Scott, 2010	Early Pennsylvanian, 323.2 to 315.2 mya	Poland	Upper Silesian Coal Basin, Moszczenica 510/1		1	39.9	319.2	320	315
Data from: Glasspool and Scott, 2010	Early Pennsylvanian, 323.2 to 315.2 mya	Poland	Upper Silesian Coal Basin, Niwka-Modrzejow 510		1	45.4	319.2	320	315

Data from: Glasspool and Scott, 2010	Early Pennsylvanian, 323.2 to 315.2 mya	Poland	Upper Silesian Coal Basin, Pn-1 Pniowek 363		1	35.1	319.2	320	315
Data from: Glasspool and Scott, 2010	Early Pennsylvanian, 323.2 to 315.2 mya	Poland	Upper Silesian Coal Basin, S-11 Silesia 214/1		1	28.6	319.2	320	315
Data from: Glasspool and Scott, 2010	Early Pennsylvanian, 323.2 to 315.2 mya	Poland	Upper Silesian Coal Basin, Silesia 308		4	16.6	319.2	320	315
Data from: Glasspool and Scott, 2010	Early Pennsylvanian, 323.2 to 315.2 mya	Poland	Upper Silesian Coal Basin, Silesia-214/1		1	28.5	319.2	320	315
Data from: Glasspool and Scott, 2010	Early Pennsylvanian, 323.2 to 315.2 mya	Poland	Upper Silesian Coal Basin, Wesoa 501		1	51.4	319.2	320	315
Data from: Glasspool and Scott, 2010	Early Pennsylvanian, 323.2 to 315.2 mya	Poland	Upper Silesian Coal Basin, Zofiowka 404/4		1	32.5	319.2	320	315
Data from: Glasspool and Scott, 2010	Early Pennsylvanian, 323.2 to 315.2 mya	Poland, Lower Silesian Basin	Coal 409		1	23.4	319.2	320	315
Data from: Glasspool and Scott, 2010	Early Pennsylvanian, 323.2 to 315.2 mya	Poland, Lower Silesian Basin	Coal 412-413		1	47.7	319.2	320	315
Data from: Glasspool and Scott, 2010	Early Pennsylvanian, 323.2 to 315.2 mya	Poland, Lower Silesian Basin	Coal 430		10	20.5	319.2	320	315
Data from: Glasspool and Scott, 2010	Early Pennsylvanian, 323.2 to 315.2 mya	Poland, Lublin Basin	Deblin Fm, Bug Mbr		1	15.5	319.2	320	315
Data from: Glasspool and Scott, 2010	Early Pennsylvanian, 323.2 to 315.2 mya	Poland, Lublin Basin	Deblin Fm, Kumow Mbr		1	14.2	319.2	320	315
Data from: Glasspool and Scott, 2010	Early Pennsylvanian, 323.2 to 315.2 mya	Poland, Lublin Basin	Lublin Fm		1	12.6	319.2	320	315
Data from: Glasspool and Scott, 2010	Early Pennsylvanian, 323.2 to 315.2 mya	Poland, Lublin Basin	Magnuszew Fm		1	14.0	319.2	320	315
Data from: Glasspool and Scott, 2010	Early Pennsylvanian, 323.2 to 315.2 mya	Poland, Upper Silesia Coal Basin	Coal 207		1	24.4	319.2	320	315
Data from: Glasspool and Scott, 2010	Early Pennsylvanian, 323.2 to 315.2 mya	Poland, Upper Silesia Coal Basin	Coal 209/210		1	24.6	319.2	320	315
Brownfield et al., 2001	Early Pennsylvanian, 323.2 to 315.2 mya	Russia, Rostovskaya, Donetsky, Central`naya i 2			1	10.0	319.2	320	315
Brownfield et al., 2001	Early Pennsylvanian, 323.2 to 315.2 mya	Russia, Rostovskaya, Donetsky, Krasnodonetskaya i 3			1	11.0	319.2	320	315
Brownfield et al., 2001	Early Pennsylvanian, 323.2 to 315.2 mya	Russia, Rostovskaya, Donetsky, Sulinskaya i 2			1	8.0	319.2	320	315
Data from: Glasspool and Scott, 2010	Early Pennsylvanian, 323.2 to 315.2 mya	Spain	Cabeza da vaca Unit, Seam 5	(Table 3)	4	8.9	319.2	320	315
Data from: Glasspool and Scott, 2010	Early Pennsylvanian, 323.2 to 315.2 mya	Spain	Aurora Unit, Candelaria Seam	(Table 3)	1	12.0	319.2	320	315
Data from: Glasspool and Scott, 2010	Early Pennsylvanian, 323.2 to 315.2 mya	Spain	Aurora Unit, Seam 6	(Table 3)	1	4.9	319.2	320	315
Data from: Glasspool and Scott, 2010	Early Pennsylvanian, 323.2 to 315.2 mya	Spain	Aurora Unit, Sucia Seam	(Table 3)	1	10.6	319.2	320	315
Data from: Glasspool and Scott, 2010	Early Pennsylvanian, 323.2 to 315.2 mya	Turkey, Zonguldak Basin	Cinarli Seam		1	14.9	319.2	320	315
Data from: Glasspool and Scott, 2010	Early Pennsylvanian, 323.2 to 315.2 mya	Ukraine, Donets Basin	1h8 Pe		1	21.0	319.2	320	315
Data from: Glasspool and Scott, 2010	Early Pennsylvanian, 323.2 to 315.2 mya	Ukraine, Donets Basin	2h10 Pe		1	15.0	319.2	320	315

Data from: Glasspool and Scott, 2010	Early Pennsylvanian, 323.2 to 315.2 mya	Ukraine, Donets Basin	3h10 Pe		1	28.0	319.2	320	315
Data from: Glasspool and Scott, 2010	Early Pennsylvanian, 323.2 to 315.2 mya	Ukraine, Donets Basin	h8 Glub		1	8.0	319.2	320	315
Data from: Glasspool and Scott, 2010	Early Pennsylvanian, 323.2 to 315.2 mya	Ukraine, Donets Basin	h8 Sha Glub		1	0.0	319.2	320	315
Data from: Glasspool and Scott, 2010	Early Pennsylvanian, 323.2 to 315.2 mya	United Kingdom, England, Disington 1 Colliery			1	28.0	319.2	320	315
Data from: Glasspool and Scott, 2010	Early Pennsylvanian, 323.2 to 315.2 mya	United Kingdom, England, Northumberland and Durham Coalfields	Plessey (M) Coal		1	12.3	319.2	320	315
Data from: Glasspool and Scott, 2010	Early Pennsylvanian, 323.2 to 315.2 mya	United Kingdom, England, Potato Pot Borehole - Westphalian A		Unclear how many seams	4	16.1	319.2	320	315
Data from: Glasspool and Scott, 2010	Early Pennsylvanian, 323.2 to 315.2 mya	United Kingdom, England, Potato Pot Borehole - Westphalian B		Unclear how many seams	4	35.4	319.2	320	315
Data from: Glasspool and Scott, 2010	Early Pennsylvanian, 323.2 to 315.2 mya	United Kingdom, England, Rowland Gill Colliery			1	3.0	319.2	320	315
Data from: Glasspool and Scott, 2010	Early Pennsylvanian, 323.2 to 315.2 mya	United Kingdom, Wales	Amman Rider Seam		1	25.8	319.2	320	315
Data from: Glasspool and Scott, 2010	Early Pennsylvanian, 323.2 to 315.2 mya	United Kingdom, Wales	Bute Seam		13	22.2	319.2	320	315
Data from: Glasspool and Scott, 2010	Early Pennsylvanian, 323.2 to 315.2 mya	USA	Amburgury Seam	(Collated from Table 6)	1	22.0	319.2	320	315
Data from: Glasspool and Scott, 2010	Early Pennsylvanian, 323.2 to 315.2 mya	USA	Elswick		1	33.6	319.2	320	315
Data from: Glasspool and Scott, 2010	Early Pennsylvanian, 323.2 to 315.2 mya	USA	River Gem		2	8.2	319.2	320	315
Data from: Glasspool and Scott, 2010	Early Pennsylvanian, 323.2 to 315.2 mya	USA, Alabama, Appalachian Coalfield	Pottsville Fm, Mary Lee		2	18.4	319.2	320	315
Data from: Glasspool and Scott, 2010	Early Pennsylvanian, 323.2 to 315.2 mya	USA, Alabama, Appalachian Coalfield	Pottsville Fm, Pratt		5	18.8	319.2	320	315
Data from: Glasspool and Scott, 2010	Early Pennsylvanian, 323.2 to 315.2 mya	USA, Illinois	Gentry		1	12.0	319.2	320	315
Data from: Glasspool and Scott, 2010	Early Pennsylvanian, 323.2 to 315.2 mya	USA, Kentucky	Blue Gem		7	8.7	319.2	320	315
Data from: Glasspool and Scott, 2010	Early Pennsylvanian, 323.2 to 315.2 mya	USA, Kentucky	Cannel City		1	66.9	319.2	320	315
Data from: Glasspool and Scott, 2010	Early Pennsylvanian, 323.2 to 315.2 mya	USA, Kentucky	Hance Fm, Upper Hance		1	19.0	319.2	320	315
Data from: Glasspool and Scott, 2010	Early Pennsylvanian, 323.2 to 315.2 mya	USA, Kentucky	Lower Breathitt Fm	Unclear how many seams	4	6.5	319.2	320	315
Data from: Glasspool and Scott, 2010	Early Pennsylvanian, 323.2 to 315.2 mya	USA, Kentucky	Manchester		1	10.5	319.2	320	315
Data from: Glasspool and Scott, 2010	Early Pennsylvanian, 323.2 to 315.2 mya	USA, Kentucky, Appalachian Coalfield	Breathitt Fm, Elkhorn - (Lower Elkhorn)		6	15.9	319.2	320	315
Data from: Glasspool and Scott, 2010	Early Pennsylvanian, 323.2 to 315.2 mya	USA, Kentucky, Appalachian Coalfield	Breathitt Fm, Elkhorn #3		2	11.2	319.2	320	315
Data from: Glasspool and Scott, 2010	Early Pennsylvanian, 323.2 to 315.2 mya	USA, Kentucky, Appalachian Coalfield	Breathitt Fm, Elkhorn #3 (Upper Elkhorn #3) / Kentucky C		19	26.7	319.2	320	315

Data from: Glasspool and Scott, 2010	Early Pennsylvanian, 323.2 to 315.2 mya	USA, Kentucky, Appalachian Coalfield	Breathitt Fm, Pond Creek		1	19.0	319.2	320	315
Data from: Glasspool and Scott, 2010	Early Pennsylvanian, 323.2 to 315.2 mya	USA, Kentucky, West Virginia, Appalachian Coalfield	Wise and Breathitt Fms, Imboden		4	12.3	319.2	320	315
Data from: Glasspool and Scott, 2010	Early Pennsylvanian, 323.2 to 315.2 mya	USA, Ohio, Appalachian Coalfield	Pottsville Gp, Sharon Coal / Ohio #1		5	13.1	319.2	320	315
Data from: Glasspool and Scott, 2010	Early Pennsylvanian, 323.2 to 315.2 mya	USA, Pennsylvania	Lykens Valley #2		1	12.9	319.2	320	315
Data from: Glasspool and Scott, 2010	Early Pennsylvanian, 323.2 to 315.2 mya	USA, Pennsylvania, Appalachian Coalfield	Freeport Fm., Allegheny Gp, Kelly		6	7.8	319.2	320	315
Data from: Glasspool and Scott, 2010	Early Pennsylvanian, 323.2 to 315.2 mya	USA, Tennessee	Bon Air Coal		16	14.7	319.2	320	315
Data from: Glasspool and Scott, 2010	Early Pennsylvanian, 323.2 to 315.2 mya	USA, Tennessee, Appalachian Coalfield	Sewanee		1	10.9	319.2	320	315
Data from: Glasspool and Scott, 2010	Early Pennsylvanian, 323.2 to 315.2 mya	USA, Virgina, Appalachian Coalfield	Norton Fm, Jawbone		1	36.1	319.2	320	315
Data from: Glasspool and Scott, 2010	Early Pennsylvanian, 323.2 to 315.2 mya	USA, Virginia, Appalachian Coalfield	Norton Fm, Banner		1	17.8	319.2	320	315
Data from: Glasspool and Scott, 2010	Early Pennsylvanian, 323.2 to 315.2 mya	USA, Virginia, Appalachian Coalfield	Norton Fm, Banner - (Lower Banner) / Gilbert		5	21.1	319.2	320	315
Data from: Glasspool and Scott, 2010	Early Pennsylvanian, 323.2 to 315.2 mya	USA, Virginia, Appalachian Coalfield	Norton Fm, Banner - (Upper Banner)		2	28.4	319.2	320	315
Data from: Glasspool and Scott, 2010	Early Pennsylvanian, 323.2 to 315.2 mya	USA, Virginia, Appalachian Coalfield	Norton Fm, Banner #3 - (Upper Banner #3)		1	22.0	319.2	320	315
Data from: Glasspool and Scott, 2010	Early Pennsylvanian, 323.2 to 315.2 mya	USA, Virginia, Appalachian Coalfield	Norton Fm, Splash Dam		3	19.7	319.2	320	315
Data from: Glasspool and Scott, 2010	Early Pennsylvanian, 323.2 to 315.2 mya	USA, Virginia, Appalachian Coalfield	Wise Fm, Lyons		1	19.1	319.2	320	315
Data from: Glasspool and Scott, 2010	Early Pennsylvanian, 323.2 to 315.2 mya	USA, West Virginia, Appalachian Coalfield	Kanawha Fm., Pottsvile Gp., Cedar Grove		4	19.7	319.2	320	315
Data from: Glasspool and Scott, 2010	Early Pennsylvanian, 323.2 to 315.2 mya	USA, West Virginia, Appalachian Coalfield	New River Fm., Pottsville Gp, Beckley		4	7.9	319.2	320	315
Data from: Glasspool and Scott, 2010	Early Pennsylvanian, 323.2 to 315.2 mya	USA, West Virginia, Appalachian Coalfield	New River Fm., Pottsville Gp, Fire Creek		4	12.7	319.2	320	315
Data from: Glasspool and Scott, 2010	Early Pennsylvanian, 323.2 to 315.2 mya	USA, West Virginia, Appalachian Coalfield	New River Fm., Pottsville Gp, Sewell		10	23.2	319.2	320	315
Data from: Glasspool and Scott, 2010	Early Pennsylvanian, 323.2 to 315.2 mya	USA, West Virginia, Appalachian Coalfield	New River Fm., Pottsville Gp, Sewell B		6	27.3	319.2	320	315
Data from: Glasspool and Scott, 2010	Early Pennsylvanian, 323.2 to 315.2 mya	USA, West Virginia, Appalachian Coalfield	Pocahontas Fm., Pottsvile Gp., Pocahontas #3		6	20.9	319.2	320	315
Data from: Glasspool and Scott, 2010	Early Pennsylvanian, 323.2 to 315.2 mya	USA, West Virginia, Appalachian Coalfield	Wise Fm, Clintwood		1	29.1	319.2	320	315
Data from: Glasspool and Scott, 2010	Serpukhovian, 330.9 to 323.2 mya	Canada, Mackenzie District	Mattson Fm, Sample C74309		1	67.0	327.1	330	330
Data from: Glasspool and Scott, 2010	Serpukhovian, 330.9 to 323.2 mya	Czech republic, Ostrava-Karvin Coalfield	Hrusov Lower		1	19.4	327.1	330	330
Data from: Glasspool and Scott, 2010	Serpukhovian, 330.9 to 323.2 mya	Czech republic, Ostrava-Karvin Coalfield	Hrusov M Upper		1	14.8	327.1	330	330
Data from: Glasspool and Scott, 2010	Serpukhovian, 330.9 to 323.2 mya	Czech republic, Ostrava-Karvin Coalfield	Jaklovec M		1	23.2	327.1	330	330

Data from: Glasspool and Scott, 2010	Serpukhovian, 330.9 to 323.2 mya	Czech republic, Ostrava-Karvin Coalfield	Petrkovice M		1	18.1	327.1	330	330
Data from: Glasspool and Scott, 2010	Serpukhovian, 330.9 to 323.2 mya	Czech republic, Ostrava-Karvin Coalfield	Poruba M		1	25.6	327.1	330	330
Data from: Glasspool and Scott, 2010	Serpukhovian, 330.9 to 323.2 mya	Poland	Lower Silesian Coal Basin, Thorez 672		1	19.8	327.1	330	330
Data from: Glasspool and Scott, 2010	Serpukhovian, 330.9 to 323.2 mya	Poland	Lower Silesian Coal Basin, Victoria 672		1	10.5	327.1	330	330
Data from: Glasspool and Scott, 2010	Serpukhovian, 330.9 to 323.2 mya	Poland	Lower Silesian Coal Basin, Victoria 673		1	16.6	327.1	330	330
Data from: Glasspool and Scott, 2010	Serpukhovian, 330.9 to 323.2 mya	Poland	Lower Silesian Coal Basin, Wabrzych 672		1	40.0	327.1	330	330
Data from: Glasspool and Scott, 2010	Serpukhovian, 330.9 to 323.2 mya	Poland	Upper Silesian Coal Basin, A-5 Anna 718		1	34.0	327.1	330	330
Data from: Glasspool and Scott, 2010	Serpukhovian, 330.9 to 323.2 mya	Poland	Upper Silesian Coal Basin, Mo-13 Moszczenica 605		1	39.9	327.1	330	330
Data from: Glasspool and Scott, 2010	Serpukhovian, 330.9 to 323.2 mya	Poland	Upper Silesian Coal Basin, Pa-1 Paskov 906		1	32.3	327.1	330	330
Data from: Glasspool and Scott, 2010	Serpukhovian, 330.9 to 323.2 mya	Poland	Walbrzych Fm, Coal 664/665		2	16.8	327.1	330	330
Data from: Glasspool and Scott, 2010	Serpukhovian, 330.9 to 323.2 mya	Poland	Walbrzych Fm, Coal 672		1	18.9	327.1	330	330
Data from: Glasspool and Scott, 2010	Serpukhovian, 330.9 to 323.2 mya	Poland	Walbrzych Fm, Coal 678		2	22.7	327.1	330	330
Data from: Glasspool and Scott, 2010	Serpukhovian, 330.9 to 323.2 mya	Poland	Walbrzych Fm, Coal 680		1	8.0	327.1	330	330
Data from: Glasspool and Scott, 2010	Serpukhovian, 330.9 to 323.2 mya	Poland, Lublin Basin	Terebin Formation		1	21.7	327.1	330	330
Data from: Glasspool and Scott, 2010	Serpukhovian, 330.9 to 323.2 mya	Ukraine, Dnepropetrovskaya	Seam c5		2	18.5	327.1	330	330
Data from: Glasspool and Scott, 2010	Serpukhovian, 330.9 to 323.2 mya	Ukraine, Dnepropetrovskaya, Donets Basin	Seam c6		3	19.7	327.1	330	330
Brownfield et al., 2001	Serpukhovian, 330.9 to 323.2 mya	Ukraine, Dnepropetrovskaya, Donetsky, Novo-Moscovskoye, Dneprovskaya c8H			1	31.0	327.1	330	330
Brownfield et al., 2001	Serpukhovian, 330.9 to 323.2 mya	Ukraine, Dnepropetrovskaya, Donetsky, Novo-Moscovskoye, Jubileinaya c6			1	18.0	327.1	330	330
Brownfield et al., 2001	Serpukhovian, 330.9 to 323.2 mya	Ukraine, Dnepropetrovskaya, Donetsky, Pavlogradskaya c5			1	18.0	327.1	330	330
Brownfield et al., 2001	Serpukhovian, 330.9 to 323.2 mya	Ukraine, Dnepropetrovskaya, Donetsky, Petrikovskoye, Geroyev Kosmosa c10B			1	28.0	327.1	330	330
Brownfield et al., 2001	Serpukhovian, 330.9 to 323.2 mya	Ukraine, Dnepropetrovskaya, Donetsky, Petrikovskoye, Lenkoma Ukrainy c8B			1	20.0	327.1	330	330
Brownfield et al., 2001	Serpukhovian, 330.9 to 323.2 mya	Ukraine, Dnepropetrovskaya, Donetsky,			1	19.0	327.1	330	330

		Petropavlovskoye, Stashkova c5							
Brownfield et al., 2001	Serpukhovian, 330.9 to 323.2 mya	Ukraine, Dnepropetrovskaya, Donetsky, Petropavlovsky, 26th syezda KPSS c6			1	18.0	327.1	330	330
Brownfield et al., 2001	Serpukhovian, 330.9 to 323.2 mya	Ukraine, Dnepropetrovskaya, Donetsky, Vostochno-Pavlogradskaya, Ternovskaya c6			1	23.0	327.1	330	330
Brownfield et al., 2001	Serpukhovian, 330.9 to 323.2 mya	Ukraine, Dnepropetrovskaya, Donetsky, Zapadno-Pavlogradskoye, Blagodatnaya c1			1	19.0	327.1	330	330
Data from: Glasspool and Scott, 2010	Serpukhovian, 330.9 to 323.2 mya	Ukraine, Dnepropetrovskaya, Novo-Moscovskoye, Donets Basin	Seam c8H		1	31.0	327.1	330	330
Brownfield et al., 2001	Serpukhovian, 330.9 to 323.2 mya	Ukraine, Dnepropetrovskaya, Pavlogradsky, Donetsky, Petropavlovsko-Mezhevoye, Pershatravneva c21			1	11.0	327.1	330	330
Brownfield et al., 2001	Serpukhovian, 330.9 to 323.2 mya	Ukraine, Dnepropetrovskaya, Pavlogradsky, Donetsky, Verkhne-Pavlogradskoye, Samarskaya c1			1	23.0	327.1	330	330
Data from: Glasspool and Scott, 2010	Serpukhovian, 330.9 to 323.2 mya	Ukraine, Dnepropetrovskaya, Petrikovskoye	Seam c10B		1	28.0	327.1	330	330
Data from: Glasspool and Scott, 2010	Serpukhovian, 330.9 to 323.2 mya	Ukraine, Dnepropetrovskaya, Petrikovskoye, Donets Basin	Seam c8B		1	20.0	327.1	330	330
Data from: Glasspool and Scott, 2010	Serpukhovian, 330.9 to 323.2 mya	Ukraine, Dnepropetrovskaya, Petropavlovsko-Mezhevoye, Donets Basin	Seam c21		1	11.0	327.1	330	330
Data from: Glasspool and Scott, 2010	Serpukhovian, 330.9 to 323.2 mya	Ukraine, Dnepropetrovskaya, Zapadno-Pavlogradskoye/Verkhne-Pavlogradskoye, Donets Basin	Seam c1		2	21.0	327.1	330	330
Data from: Glasspool and Scott, 2010	Serpukhovian, 330.9 to 323.2 mya	Ukraine, Donets Basin	1c10 YD		1	16.0	327.1	330	330
Data from: Glasspool and Scott, 2010	Serpukhovian, 330.9 to 323.2 mya	Ukraine, Donets Basin	2c10 Yd		1	21.0	327.1	330	330
Data from: Glasspool and Scott, 2010	Serpukhovian, 330.9 to 323.2 mya	Ukraine, Donets Basin	3c10 YD		1	26.0	327.1	330	330
Data from: Glasspool and Scott, 2010	Serpukhovian, 330.9 to 323.2 mya	Ukraine, Donets Basin	c11 YD		1	29.0	327.1	330	330
Data from: Glasspool and Scott, 2010	Serpukhovian, 330.9 to 323.2 mya	Ukraine, Donets Basin	d4 Krw		1	2.0	327.1	330	330
Data from: Glasspool and Scott, 2010	Early Viséan, 346.7 to 330.9 mya	Australia, Hunter Valley	Conger Fm	Unclear how many seams	2	25.4	338.8	340	345
Data from: Glasspool and Scott, 2010	Late Visean, 346.7 to 330.9 mya	Canada, Mackenzie District	Mattson Fm, Sample C58730		1	57.0	338.8	340	345
Data from: Glasspool and Scott, 2010	Late Visean, 346.7 to 330.9 mya	Canada, Mackenzie District	Mattson Fm, Sample C58737		1	67.0	338.8	340	345

Data from: Glasspool and Scott, 2010	Late Visean, 346.7 to 330.9 mya	Canada, Mackenzie District	Mattson Fm, Sample C58764		1	57.0	338.8	340	345
Data from: Glasspool and Scott, 2010	Viséan , 346.7 to 330.9 mya	Canada, Northern Yukon Territory	Kayak Fm	Unclear how many seams	7	38.7	338.8	340	345
Data from: Glasspool and Scott, 2010	Late Visean, 346.7 to 330.9 mya	China, Middle Hunan Province	Tseshui Fm	Unclear how many seams	7	12.7	338.8	340	345
Data from: Glasspool and Scott, 2010	Late Visean, 346.7 to 330.9 mya	Germany, Borna Hainichen			1	19.5	338.8	340	345
New data from: ProNina, this paper	Early Viséan, 346.7 to 330.9 mya	Russia, Kama Basin			1	40.0	338.8	340	345
New data from: ProNina, this paper	Late Visean, 346.7 to 330.9 mya	Kazakhstan, Karagandinskaya, Karaganda Basin - 1st gp of seams (seams chiefly at top of the Karaganda Series, not those in the middle = second gp)			1	29.0	338.8	340	345
Brownfield et al., 2001	Viséan , 346.7 to 330.9 mya	Kazakhstan, Karagandinskaya, Molodiozhny, Karagandinsky, Borly, Borlinnsky Nizhny	Mississippian, Visean, Visean 2-3		1	29.0	338.8	340	345
Brownfield et al., 2001	Viséan , 346.7 to 330.9 mya	Russia, Kaluzhskaya, Podmoscovny, Seredeyskoye, Seredeyskaya 2 II	Mississippian, Visean, Early-Middle Visean (Bobrikovian-Tulian)		1	26.0	338.8	340	345
Data from: Glasspool and Scott, 2010	Late Visean, 346.7 to 330.9 mya	Russia, Moscow Basin		Unclear how many seams	5	9.7	338.8	340	345
Brownfield et al., 2001	Viséan , 346.7 to 330.9 mya	Russia, Smolenskaya, Podmoscovny, Bol`shoye Nelidovskoye, Nelidovskaya II	Mississippian, Visean, Early-Middle Visean (Bobrikovian-Tulian)		1	25.0	338.8	340	345
New data from: ProNina, this paper	Early Viséan, 346.7 to 330.9 mya	Russia, Smolenskaya, Podmoscovny, Bol`shoye Nelidovskoye, Nelidovskaya II			1	25.0	338.8	340	345
Brownfield et al., 2001	Viséan , 346.7 to 330.9 mya	Russia, Smolenskaya, Podmoscovny, Safonovskoye, Safonovskaya 7 II	Mississippian, Visean, Early-Middle Visean (Bobrikovian-Tulian)		1	27.0	338.8	340	345
New data from: ProNina, this paper	Early Viséan, 346.7 to 330.9 mya	Russia, Smolenskaya, Podmoscovny, Safonovskoye, Safonovskaya 7 II			1	27.0	338.8	340	345
Brownfield et al., 2001	Viséan , 346.7 to 330.9 mya	Russia, Tul`skaya, Podmoscovny, L`vovskoye, L`vovskaya IV	Mississippian, Visean, Early-Middle Visean (Bobrikovian-Tulian)		1	26.0	338.8	340	345
New data from: ProNina, this paper	Early Viséan, 346.7 to 330.9 mya	Russia, Tul`skaya, Podmoscovny, L`vovskoye, L`vovskaya IV			1	26.0	338.8	340	345
Brownfield et al., 2001	Viséan , 346.7 to 330.9 mya	Russia, Tul`skaya, Podmoscovny, Lipkovskoye, Lipkovskaya 9 III	Mississippian, Visean, Early-Middle Visean (Bobrikovian-Tulian)		1	25.0	338.8	340	345
New data from: ProNina, this paper	Early Viséan, 346.7 to 330.9 mya	Russia, Tul`skaya, Podmoscovny, Lipkovskoye, Lipkovskaya 9 III			1	25.0	338.8	340	345
Brownfield et al., 2001	Viséan , 346.7 to 330.9 mya	Russia, Tul`skaya, Podmoscovny,	Mississippian, Visean, Early-Middle Visean		1	28.0	338.8	340	345

		Smorodinskoye, Mayskaya III v	(Bobrikovian-Tulian)						
New data from: ProNina, this paper	Early Viséan, 346.7 to 330.9 mya	Russia, Tul`skaya, Podmoscovny, Smorodinskoye, Mayskaya III v			1	28.0	338.8	340	345
Data from: Glasspool and Scott, 2010	Late Visean, 346.7 to 330.9 mya	Svalbard, Spitsbergen	Svenbreen Fm, Birger Johnsonfjellet Mbr, S6	excludes micrinite	1	10.0	338.8	340	345
Data from: Glasspool and Scott, 2010	Late Visean, 346.7 to 330.9 mya	Svalbard, Spitsbergen	Svenbreen Fm, Birger Johnsonfjellet Mbr, S7	excludes micrinite	1	3.0	338.8	340	345
Data from: Glasspool and Scott, 2010	Early Viséan, 346.7 to 330.9 mya	Svalbard, Spitsbergen, Mumien	Mumien Fm		1	29.2	338.8	340	345
Romero-Sarmiento et al., 2011	Late Visean, 346.7 to 330.9 mya	Scotland, East Lothian, Dunbar	Lower Limestone Fm., Horizon SC-2 below Chapel Point Limestone		1	20.0	338.8	340	345
Romero-Sarmiento et al., 2011	Late Visean, 346.7 to 330.9 mya	Scotland, East Lothian, Dunbar	Lower Limestone Fm., Longcraig Coal		1	18.0	338.8	340	345
Data from: Glasspool and Scott, 2010	Tournaisian, 358.9 to 346.7 mya	Russia, Permskaya, Kizelovsky	Seam 13		2	25.0	352.8	350	360
Data from: Glasspool and Scott, 2010	Tournaisian, 358.9 to 346.7 mya	Russia, Permskaya, Kizelovsky	Seam 5		2	21.0	352.8	350	360
Data from: Glasspool and Scott, 2010	Tournaisian, 358.9 to 346.7 mya	Russia, Permskaya, Kizelovsky, Kospashskoye	Seam 9		1	22.0	352.8	350	360
Data from: Glasspool and Scott, 2010	Tournaisian, 358.9 to 346.7 mya	Russia, Permskaya, Kizelovsky, Shumikhinskoye	Seam 11		1	28.0	352.8	350	360
Data from: Glasspool and Scott, 2010	Tournaisian, 358.9 to 346.7 mya	Norway, Svalbard, Spitsbergen	Horbybreen Fm, Hoelbreen Mbr, BJ1	excludes micrinite	1	49.0	352.8	350	360
Data from: Glasspool and Scott, 2010	Tournaisian, 358.9 to 346.7 mya	Norway, Svalbard, Spitsbergen	Horbybreen Fm, Hoelbreen Mbr, S3	excludes micrinite	1	24.0	352.8	350	360
Data from: Glasspool and Scott, 2010	Tournaisian, 358.9 to 346.7 mya	Norway, Svalbard, Spitsbergen	Horbybreen Fm, Hoelbreen Mbr, S4	excludes micrinite	1	51.0	352.8	350	360
Michelsen and Khorasani, 1991	Late Famennian, 372.2 to 358.9 mya	Norway, Bjornoya (Bear Island)	Roedvika Fm., Tunheim Mbr Seam A		11	36.7	365.6	370	360
Michelsen and Khorasani, 1991	Late Famennian, 372.2 to 358.9 mya	Norway, Bjornoya (Bear Island)	Roedvika Fm., Tunheim Mbr Seam B		7	61.9	365.6	370	360
Data from: Glasspool and Scott, 2010	Early to Middle Frasnian, 382.7 to 372.2 mya	Canada, W. Melville Island	Beverley Inlet Fm	Unclear how many seams	6	0.7	377.5	380	360
Data from: Glasspool and Scott, 2010	Late Givetian to Middle Frasnian, 387.7 to 372.2 mya	Canada, W. Melville Island	Hecla Bay Fm	Unclear how many seams	13	2.3	380.0	380	360
Data from: Glasspool and Scott, 2010	Late Givetian to Middle Frasnian, 387.7 to 372.2 mya	Canada, W. Melville Island	Weatherall, Fm	Sample 14 excluded due to excesive mineral matter; Unclear how many seams	7	0.9	380.0	380	360
Data from: Glasspool and Scott, 2010	Givetian to Frasnian boundary, 382.7 to 382.7 mya	Norway, Svalbard, Spitsbergen, Mimerdalen			1	0.0	382.7	380	375
Ghori, 1999	Givetian, 387.7 to 382.7 mya	Australia, Carnarvon Basin, CRAE GBH 1 Well	Nannyarra Sandstone, 272.6-272.2m depth, unnamed coal		1	2.0	385.2	390	375
Ghori, 1999	Givetian, 387.7 to 382.7 mya	Australia, Carnarvon Basin, CRAE GBH 1 Well	Nannyarra Sandstone, 344.1-344.3m depth, unnamed coal		1	0.0	385.2	390	375
Data from: Glasspool and Scott, 2010	Givetian, 387.7 to 382.7 mya	China			1	0.0	385.2	390	375

Data from: Glasspool and Scott, 2010	Givetian, 387.7 to 382.7 mya	China			1	0.0	385.2	390	375
Data from: Glasspool and Scott, 2010	Givetian, 387.7 to 382.7 mya	China, Luquan	Luquan	(Table 2)	1	0.1	385.2	390	375
Xu et al., 2012	Givetian, 387.7 to 382.7 mya	China, North Xinjiang, 251 Hill ca. 500m west of Highway G217, ca. 20km north of Hoxtolgay Town, Hoboksar Mongol Autonomous County (GPS: 46◦36’55”N, 86◦1’5”E) Sample B24/6	Givetian, Hujiersite Formation		1	0.0	385.2	390	375
Data from: Glasspool and Scott, 2010	Eifelian-Givetian, 393.3 to 382.7 mya	Estonia, Barzas	Barzas Coal	Unclear how many seams	4	1.5	388.0	390	375
Peppers and Damberger, 1969 (Age: Nelson and Marshak, 1996)	Eifelian, 393.3 to 387.7 mya	USA, Illinois, McLean and DeWitt Counties	Wapsipinicon Formation, Davenport Limestone Member		3	0.0	390.5	390	375
Data from: Glasspool and Scott, 2010	Early Emsian, 407.6 to 393.3 mya	Canada, Gaspe, Tar Point	L'Anse-a-Brillant Coal	Vit-287, Inert-1, Lip-139, Min-73 (Count 500); MMF - Vit 67.2%, Inert 0.2%, Lip 32.6%	1	0.2	400.5	400	390
