# Supplementary material for: The impact of fire on the Late Paleozoic Earth system
Source: Front Plant Sci. 2015 Sep 23;6:756. doi: 10.3389/fpls.2015.00756 (PMC4585212; doi:10.3389/fpls.2015.00756)
Supplement: Supplementary file 2 [file Table_2.PDF]

10 Million year binned data					
**Bin**	**Mean**	**Std. err.**	**p(O2)Best est.**	**p(O2)min -1sd**	**p(O2)min +1sd**
240	2.2	0.9	20.3	19.2	21.3
250	39.1	2.1	26.6	25.7	28.0
260	27.3	1.7	25.3	24.4	26.5
270	22.3	1.8	24.7	23.8	25.9
280	50.4	1.9	27.8	26.8	29.2
290	52.2	1.2	27.9	27.0	29.3
300	37.3	4.1	26.5	25.3	28.0
310	14.5	0.5	23.6	22.8	24.4
320	22.5	1.0	24.7	23.9	25.8
330	22.5	1.7	24.7	23.8	25.9
340	27.9	2.7	25.4	24.4	26.7
350	31.4	4.9	25.8	24.5	27.4
360					
370	49.3	12.6	27.7	25.6	30.3
380	1.0	0.5	19.4	18.4	20.2
390	0.5	0.3	18.7	17.7	19.5
400	0.2	0.0	18.2	17.9	18.4
**Formula**			((35-16)*ACOS((1-2*((Mean Inert%/100)^(1/1.8))))/PI())+16	((34-16)*ACOS((1-2*(((Mean Inert%-Std. Error)/100)^(1/1.7))))/PI())+16	((37-16)*ACOS((1-2*(((Mean Inert%+Std. Error)/100)^(1/1.8))))/PI())+16
**15 Million year binned data**					
**Bin**	**Mean**	**Std. err.**	**p(O2)Best est.**	**p(O2)min -1sd**	**p(O2)min +1sd**
240	2.4	0.9	20.4	19.4	21.3
255	32.2	1.4	25.9	25.0	27.1
270	22.3	1.8	24.7	23.8	25.9
285	54.7	1.1	28.2	27.3	29.6
300	38.8	2.0	26.6	25.6	27.9
315	17.4	0.5	24.0	23.3	24.9
330	22.5	1.7	24.7	23.8	25.9
345	27.9	2.7	25.4	24.4	26.7
360	26.9	5.8	25.3	23.8	27.0
375	0.4	0.3	18.6	17.7	19.3
390	0.2	0.0	18.2	17.9	18.4
**Formula**			((35-16)*ACOS((1-2*((Mean Inert%/100)^(1/1.8))))/PI())+16	((34-16)*ACOS((1-2*(((Mean Inert%-Std. Error)/100)^(1/1.7))))/PI())+16	((37-16)*ACOS((1-2*(((Mean Inert%+Std. Error)/100)^(1/1.8))))/PI())+16
