# Supplementary material for: The impact of fire on the Late Paleozoic Earth system
Source: Front Plant Sci. 2015 Sep 23;6:756. doi: 10.3389/fpls.2015.00756 (PMC4585212; doi:10.3389/fpls.2015.00756)
Supplement: Supplementary file 3 [file Table_3.PDF]

Reference	Age	Locality	Stratigraphy	Notes	No. samples	Average inertinite mmf %	MYA	10 myr bin	15myr bin
Data from: Glasspool and Scott, 2010	Holocene, 0.0117 to 0 mya	Canada, Ontario	Albion Road Swamp Peat	measured from fig. 3 incl. funginite	8	8.1	0.01	0	0
Data from: Glasspool and Scott, 2010	Holocene, 0.0117 to 0 mya	Canada, Ontario	Elice Bog Peat	measured from fig. 3 incl. funginite	8	2.8	0.01	0	0
Data from: Glasspool and Scott, 2010	Holocene, 0.0117 to 0 mya	Canada, Ontario	Interior Fen Peat	measured from fig. 3 incl. funginite	10	6.1	0.01	0	0
Data from: Glasspool and Scott, 2010	Holocene, 0.0117 to 0 mya	Canada, Ontario	Kenoje Lake Bog Peat	measured from fig. 3 incl. funginite	15	11.1	0.01	0	0
Data from: Glasspool and Scott, 2010	Holocene, 0.0117 to 0 mya	Czech Republic, Hajek	Modern, Sedge and diatoms		1	5.4	0.01	0	0
Data from: Glasspool and Scott, 2010	Holocene, 0.0117 to 0 mya	Finland	Modern, Carex		1	4.2	0.01	0	0
Data from: Glasspool and Scott, 2010	Holocene, 0.0117 to 0 mya	Finland	Modern, Carex		1	4.8	0.01	0	0
Data from: Glasspool and Scott, 2010	Holocene, 0.0117 to 0 mya	Indonesia, Kalimantan		Eight Samples - data indivisible	8	8.0	0.01	0	0
Data from: Glasspool and Scott, 2010	Holocene, 0.0117 to 0 mya	Indonesia, Kalimantan	Modern, Combretocarpus		1	4.4	0.01	0	0
Data from: Glasspool and Scott, 2010	Holocene, 0.0117 to 0 mya	Indonesia, Kalimantan	Modern, Mixed Swamp		1	7.8	0.01	0	0
Data from: Glasspool and Scott, 2010	Holocene, 0.0117 to 0 mya	Netherlands	Modern, Alnus		1	4.8	0.01	0	0
Data from: Glasspool and Scott, 2010	Holocene, 0.0117 to 0 mya	Netherlands	Modern, Phramites		1	0.2	0.01	0	0
Data from: Glasspool and Scott, 2010	Holocene, 0.0117 to 0 mya	Netherlands	Modern, Phramites		1	1.4	0.01	0	0
Data from: Glasspool and Scott, 2010	Holocene, 0.0117 to 0 mya	USA, Florida	Modern, Mariscus		1	4.0	0.01	0	0
Data from: Glasspool and Scott, 2010	Holocene, 0.0117 to 0 mya	USA, Florida	Modern, Rhizophora		1	0.0	0.01	0	0
Data from: Glasspool and Scott, 2010	Holocene, 0.0117 to 0 mya	USA, Georgia, Okeefenokee	Modern, Nymphaea		1	4.6	0.01	0	0
Data from: Glasspool and Scott, 2010	Holocene, 0.0117 to 0 mya	USA, Georgia, Okeefenokee	Modern, Taxodium		1	6.0	0.01	0	0
Data from: Glasspool and Scott, 2010	Pleistocene, 2.588 to 0.0117 mya	China, Western Yunnan, Tenchong Basin	Peat in upper cycle	May represent more than one seam, but this is unclear from the text	11	1.0	1.30	0	0
Data from: Glasspool and Scott, 2010	Pleistocene, 2.588 to 0.0117 mya	China, Western Yunnan, Tenchong Basin	Soft brown coal in lower cycle		1	2.0	1.30	0	0
Data from: Glasspool and Scott, 2010	Pleistocene, 2.588 to 0.0117 mya	Greece, Megalopolis			1	2.0	1.30	0	0
Data from: Glasspool and Scott, 2010	Pleistocene, 2.588 to 0.0117 mya	Greece, Megalopolis Basin	Marathousa Member, Seam 1		9	0.9	1.30	0	0
Data from: Glasspool and Scott, 2010	Pliocene, 5.333 to 2.588 mya	China	Pliocene		1	2.8	3.96	0	0
Data from: Glasspool and Scott, 2010	Pliocene, 5.333 to 2.588 mya	China	Pliocene		1	4.5	3.96	0	0

Data from: Glasspool and Scott, 2010	Pliocene, 5.333 to 2.588 mya	China	Pliocene		1	4.3	3.96	0	0
Data from: Glasspool and Scott, 2010	Pliocene, 5.333 to 2.588 mya	China, Yunnan, Liahou Mine		(One Seam)	9	1.1	3.96	0	0
Data from: Glasspool and Scott, 2010	Pliocene, 5.333 to 2.588 mya	Greece	Amynteon-Ptolemaida Lignite		1	6.2	3.96	0	0
Data from: Glasspool and Scott, 2010	Pliocene, 5.333 to 2.588 mya	Greece, Apofysis mine, Amynteo Basin		(One Seam)	1	11.0	3.96	0	0
Tewalt et al., 2010	Pliocene, 5.333 to 2.588 mya	Greece, Choremi Mine	Pliocene, Sample # GR-1		1	11.9	3.96	0	0
Tewalt et al., 2010	Pliocene, 5.333 to 2.588 mya	Greece, Choremi Mine	Pliocene, Sample # GR-2		1	7.2	3.96	0	0
Data from: Glasspool and Scott, 2010	Pliocene, 5.333 to 2.588 mya	Greece, Florina Coalfield		May represent more than one seam, but this is unclear from the text	9	3.2	3.96	0	0
Data from: Glasspool and Scott, 2010	Pliocene, 5.333 to 2.588 mya	Greece, Kalavryta Coalfield		May represent more than one seam, but this is unclear from the text	2	2.0	3.96	0	0
Data from: Glasspool and Scott, 2010	Pliocene, 5.333 to 2.588 mya	Greece, Ptolemais/Amynteon Coalfield			1	17.0	3.96	0	0
Sia and Abdullah, 2012	Upper Pliocene, 5.333 to 2.588 mya	Malaysia, Sarawak	Balingian Coal		1	2.5	3.96	0	0
Data from: Glasspool and Scott, 2010	Pliocene, 5.333 to 2.588 mya	Portugal	Rio Maior Lignite		24	9.2	3.96	0	0
Chiriac et al., 2007	Pliocene, 5.333 to 2.588 mya	Romania, Rosia de Jiu	Pliocene Lignite	1%inert 36% Min	1	1.6	3.96	0	0
