# Supplementary material for: The impact of fire on the Late Paleozoic Earth system
Source: Front Plant Sci. 2015 Sep 23;6:756. doi: 10.3389/fpls.2015.00756 (PMC4585212; doi:10.3389/fpls.2015.00756)
Supplement: Supplementary file 4 [file Table_4.PDF]

	10 and 15 myr bins (Holocene only)	10 and 15 myr bins (Holocene to Pleistocene)	10 and 15 myr bins (Holocene to Pliocene)
**Mean**	4.92	4.27	4.97
**Standard error**	0.70	0.64	0.64
**Standard deviation**	2.89	2.95	3.79
**Count**	17	21	35
